# Concentration Recognition‐Based Auto‐Dynamic Regulation System (CRUISE) Enabling Efficient Production of Higher Alcohols

**DOI:** 10.1002/advs.202310215

**Published:** 2024-04-16

**Authors:** Zhenya Chen, Shengzhu Yu, Jing Liu, Liwei Guo, Tong Wu, Peifeng Duan, Dongli Yan, Chaoyong Huang, Yi‐Xin Huo

**Affiliations:** ^1^ Key Laboratory of Molecular Medicine and Biotherapy Aerospace Center Hospital School of Life Science Beijing Institute of Technology Haidian District No. 5 South Zhongguancun Street Beijing 100081 China; ^2^ Tangshan Research Institute Beijing Institute of Technology, No. 57, South Jianshe Road, Lubei District Tangshan Hebei 063000 China

**Keywords:** amino acids, continue production, dynamic regulation, higher alcohols, self‐assembly

## Abstract

Microbial factories lacking the ability of dynamically regulating the pathway enzymes overexpression, according to in situ metabolite concentrations, are suboptimal, especially when the metabolic intermediates are competed by growth and chemical production. The production of higher alcohols (HAs), which hijacks the amino acids (AAs) from protein biosynthesis, minimizes the intracellular concentration of AAs and thus inhibits the host growth. To balance the resource allocation and maintain stable AA flux, this work utilizes AA‐responsive transcriptional attenuator *ivbL* and HA‐responsive transcriptional activator BmoR to establish a concentration recognition‐based auto‐dynamic regulation system (CRUISE). This system ultimately maintains the intracellular homeostasis of AA and maximizes the production of HA. It is demonstrated that *ivbL*‐driven enzymes overexpression can dynamically regulate the AA‐to‐HA conversion while BmoR‐driven enzymes overexpression can accelerate the AA biosynthesis during the HA production in a feedback activation mode. The AA flux in biosynthesis and conversion pathways is balanced via the intracellular AA concentration, which is vice versa stabilized by the competition between AA biosynthesis and conversion. The CRUISE, further aided by scaffold‐based self‐assembly, enables 40.4 g L^−1^ of isobutanol production in a bioreactor. Taken together, CRUISE realizes robust HA production and sheds new light on the dynamic flux control during the process of chemical production.

## Graphical abstract

This work develops a CRUISE that can sustain the long‐term production of biofuel higher alcohols (HAs), which hijacks the intermediates of amino acids (AAs) for protein biosynthesis, through balancing the AA flux in biosynthesis and conversion pathways via the intracellular AA concentration, which is vice versa stabilized by the competition between AA biosynthesis and conversion.

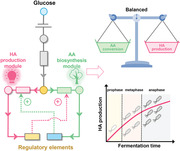

## 1 Introduction

Microbial‐based metabolic engineering is a powerful approach for green and economical production of value‐added compounds including bulk and fine chemicals,^[^ ^1^, ^2^ ^]^ biodegradable materials,^[^ ^3^, ^4^ ^]^ pharmaceuticals,^[^ ^5^, ^6^ ^]^ and functional healthcare products.^[^ ^7^, ^8^ ^]^ The incompatibility of microbes with exogenous production pathways leads to an uneven allocation of resources between growth and production, especially in cases where growth and production compete for the crucial intermediates. Microbes are blind to the concentration changes of key intermediates involved in exogenous production pathway, and thus unable to dynamically regulate the growth‐ or production‐targeted metabolic network.^[^ ^9^ ^]^

To maximize the titer, yield, and productivity of the bioconversion process under the inherent production mode, in which growth and chemicals production are independent of each other, non‐dynamic strategies such as engineering and overexpression of the key pathway enzymes,^[^ ^10^, ^11^, ^12^ ^]^ knockout of the competing pathways,^[^ ^13^, ^14^ ^]^ or redirection of the carbon flux^[^ ^15^, ^16^ ^]^ were developed. However, these non‐dynamic strategies are suboptimal because the carbon flux could not be modified according to the real‐time resource status of the microbes.^[^ ^17^, ^18^, ^19^ ^]^ The occasional imbalance of the resource allocation results in the difficulties for sustaining the long‐term growth and production and thus restricts the microbes from optimal chemical production.^[^ ^20^, ^21^ ^]^ Therefore, dynamic regulation strategies aiming to regulate the enzymes overexpression according to the corresponding concentration changes of one or several intermediates, have emerged as an attractive way to optimize the intracellular resource allocation to satisfy the real‐time demands of microbes.^[^ ^9^ ^]^

**Figure 1:**
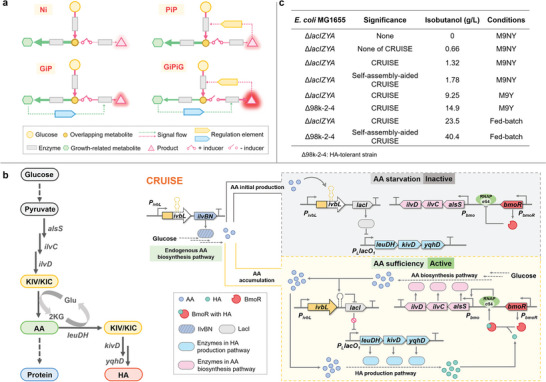
Design of the CRUISE and the corresponding isobutanol production. a) The production modes. Ni: non‐induced; GiP: growth induced production; PiP: production induced production; GiPiG: growth induced production, production induced re‐growth, re‐growth induced re‐production. b) The pathways for AA biosynthesis and HA production. AlsS, acetolactate synthase; IlvC, acetohydroxy acid isomeroreductase; IlvD, dihydroxy‐acid dehydratase; LeuDH, leucine dehydrogenase; KivD, ketoisovalerate decarboxylase; YqhD, alcohol dehydrogenase; KIV, 2‐ketoisovalerate; KIC: 2‐ketoisocaproate. The CRUISE coupled AA‐concentration‐regulated *ivbL* transcriptional attenuation system and HA‐concentration‐regulated BmoR transcriptional activation system. For AA starvation, microbes closed the AA‐to‐HA conversion and biosynthesized AA via the endogenous AA biosynthesis pathway. For AA sufficiency, microbes turned on the AA‐to‐HA conversion. The generated HA bound to BmoR to enhance AA biosynthesis in a feedback activation mode, thereby enabling the continuous AA‐to‐HA conversion. c) The corresponding isobutanol production.

Dynamic regulation was commonly realized by the biosensors composed of transcription factors (TFs),^[^ ^22^, ^23^ ^]^ which are transcriptional regulatory proteins undergoing a conformational change after binding signal molecules (SMs) to activate or suppress the TF‐regulated transcription initiation in a dose‐of‐SM dependent manner.^[^ ^24^ ^]^ For example, acetyl‐CoA,^[^ ^25^, ^26^, ^27^ ^]^ malonyl‐CoA,^[^ ^28^, ^29^, ^30^ ^]^ pyruvate,^[^ ^31^, ^32^ ^]^ and fructose‐1,6‐bisphosphate^[^ ^33^ ^]^ are growth intermediates (GIs) that accumulate during growth. They could either be converted to biomass by growth pathways, or be converted to chemical products (CPs) such as resveratrol,^[^ ^34^ ^]^ vanillic acid,^[^ ^35^ ^]^ and naringenin^[^ ^36^ ^]^ by production pathways. Biosensors using GIs or CPs as SMs have been used to construct the gene circuits, enabling the unidirectional dynamic regulations of key enzyme overexpression.^[^ ^37^, ^38^, ^39^ ^]^ For example, GIs could be used to activate GI‐responsive biosensors to initiate the GIs‐to‐CPs conversion,^[^ ^40^ ^]^ forming a mode of “growth induced production” (GiP) (**Figure** **1**a). On the other hand, CPs could be used to activate CP‐responsive biosensors to activate the carbon‐source‐to‐GIs or GIs‐to‐CPs conversion,^[^ ^28^ ^]^ or to inhibit GIs‐to‐byproducts conversion, forming a mode of “production induced production” (PiP). The GiP or PiP mode aims to maximize the CPs accumulation, but could not dynamically respond to the concentrations of GIs. The conversion of GIs to CPs or biomass could hardly be balanced during the whole production process.

Higher alcohols (HAs) isobutanol and isopentanol are derivatives of amino acids (AAs) L‐Val and L‐Leu, respectively (Figure 1b), and have been expected to be the next generation transportation fuels because of the low vapor pressure and high energy density.^[^ ^41^ ^]^ Our lab has developed *Pseudomonas butanovora* activator BmoR,^[^ ^42^ ^]^ a TF of C2‐C5 linear or branched‐chain alcohols (Figure S1, Supporting Information), into a biosensor family containing BmoR mutants with expanded ligand profile, tightened ligand specificity, widened responsive concentration, increased detection sensitivity, and amplified output strength.^[^ ^43^, ^44^, ^45^ ^]^

The production of HAs competed the AAs with the protein biosynthesis and minimized the intracellular AA concentration, inhibiting the host growth.^[^ ^46^ ^]^ This prevented the microbes from intelligently balancing resource allocation to maintain the continuous AA‐to‐HA conversion. In response to this challenge, this study developed a concentration recognition‐based auto‐dynamic regulation system (CRUISE) (Figure 1b) to form a circular feedback mode of “growth induced production, production induced re‐growth, re‐growth induced re‐production” (GiPiG). In this system, the AA‐dependent transcriptional attenuator *ivbL* could dynamically regulate the overexpression of the enzymes that catalyzed the AA‐to‐HA conversion, while the HA‐dependent transcriptional activator BmoR could dynamically regulate the overexpression of the enzymes that accelerated the AA biosynthesis during the HA production in a feedback activation mode. The presence of CRUISE promoted the microbes to break their inherent production mode and to start the AA‐to‐HA conversion in the initial stage of fermentation, forming a GiPiG mode to enable the continuous AA‐to‐HA conversion throughout the fermentation process, improving the isobutanol production titer to 23.5 g L^−1^ in a bioreactor. Furthermore, 40.4 g L^−1^ isobutanol was produced by a HA‐tolerant strain in a bioreactor via adding the scaffold‐based self‐assembly into the CRUISE (Figure 1c; Table S1, Supporting Information). Taken together, the CRUISE realized the spiral mutual response between AA and HA through AA‐ and HA‐responsive biosensors, enabling dynamic flux control and robust HA production during the process of chemical production.

## 2 Results

### 2.1 AA‐ and HA‐Responsive Genetic Circuit of the CRUISE

Here, we established a CRUISE to couple AA‐concentration‐regulated *ivbL* transcriptional attenuation system and HA‐concentration‐regulated BmoR transcriptional activation system (Figure 1b), allowing the real‐time coordination of the AA biosynthesis and the HA production. In this system, the *ivbL* system was engineered to regulate the enzymes overexpression in HA production pathways, while the BmoR system was engineered to regulate the enzymes expression in AA biosynthesis pathways. *Escherichia coli ivbL* is an attenuator that is responsible for regulating the enzymes expression in the biosynthetic pathways of branched‐chain AAs L‐Val, L‐Leu and L‐Ile (Figure S2, Supporting Information).

**Figure 2:**
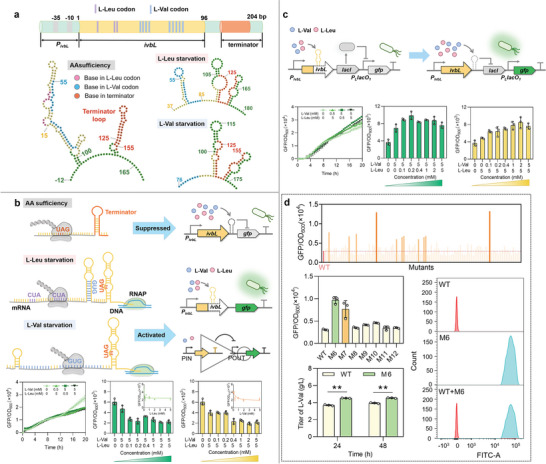
AA‐concentration‐regulated *ivbL* transcriptional attenuation system. a) The sequence of the *ivbL* transcriptional attenuation system. The *ivbL* transcriptional attenuation system is responsible for regulating the expression of acetylhydroxyacid synthase IlvBN. The polypeptide *ivbL* (96 bp) is located downstream of the promoter *P_ivbL_*. b) Validation of the transcriptional attenuation of *ivbL* system toward L‐Val or L‐Leu. In the presence of L‐Val or L‐Leu feeding could inhibit the expression of GFP. The logic gate of “NOT GATE” was formed with L‐Val or L‐Leu as input and GFP as output. c) *IvbL*‐dependent inducible cascade activation system. In the presence of L‐Val or L‐Leu feeding could induce the expression of GFP. d) Screening of L‐Val overproducers using the inducible cascade activation system, and testing of the screening efficiency via FACS technique. Values and error bars represent mean and SD (*n* = 3), respectively. ^∗^ *P* < 0.1, ^∗∗^ *P* < 0.01, as determined by two‐tailed *t*‐test.

The turn on or off of the *ivbL* system relies on the adjustable translation of the *ivbL* mRNA, which has 32 codons containing twelve L‐Val and L‐Leu codons (**Figure** **2**a). When intracellular concentrations of L‐Val and L‐Leu are high, amount of aminoacyl tRNA carrying L‐Val and L‐Leu are abundant to complete the *ivbL* translation and release the ribosome, leading the palindromic G‐C rich sequence downstream of UAG to form terminator and stop the transcription of the downstream *ilvBN* genes, thus in turn inhibits the biosynthesis of L‐Val or L‐Leu. When intracellular L‐Val or L‐Leu is deficient, the shortage of aminoacyl L‐Val or L‐Leu leads to the stall of ribosome in the middle of *ivbL* mRNA, effectively avoiding the formation of terminator and allowing the transcription of *ilvBN* genes to initiate the biosynthesis of L‐Val or L‐Leu (Figure S2, Supporting Information). We simulated the secondary structures of *ivbL* by RNAfold (http://rna.tbi.univie.ac.at//cgi‐bin/RNAWebSuite/RNAfold.cgi), showing that the *ivbL* mRNA could form a terminator loop only when L‐Val or L‐Leu was sufficient (Figure 2a).

In the CRUISE, repressor *lacI* was placed downstream of *ivbL* gene to form a co‐transcribed operon, while the LacI‐regulated promoter *P_L_lacO_1_* drove the expression of LeuDH, KivD and YqhD, which could sequentially convert AAs, e.g. L‐Val and L‐Leu, to HAs, e.g. isobutanol and isopentanol, respectively. On the other hand, the BmoR‐regulated *P_bmo_* drove the overexpression of AlsS, IlvC and IlvD, which are responsible for the AA biosynthesis (Figure 1b). These designs established an AA concentration‐responsive system to regulate HA production. Using this system in *E. coli*, the AA‐to‐HA conversion was turned off in the presence of low concentration of AA, which was gradually produced through the endogenous biosynthesis pathway in the initial stage of fermentation. When sufficient amount of AA accumulated, the AA‐to‐HA conversion was activated by the initiation of the expression of LeuDH, KivD, and YqhD. The generated HA bound to BmoR to initiate the expression of AlsS, IlvC, and IlvD under the control of *P*_bmo_, which increased the AA biosynthesis in a feedback activation mode, facilitating the AA‐to‐HA conversion and ultimately achieving the continuous production of HA.

### 2.2 AA‐Concentration‐Regulated *ivbL* Transcriptional Attenuation System

To explore the dose effect of AA on *ivbL* system in the CRUISE, the *gfp* reporter was placed downstream of *ivbL* gene to form a co‐transcribed operon in plasmid pS‐iG (Figure 2b). Overexpression of GFP reporter should be turned on or off in the absence or presence of AA feeding, respectively, forming a “NOT GATE” logic gate using AA as input and GFP as output. The transcriptional attenuation effect was verified by *E. coli* MG1655Δ*lacIZYA* strain harboring pS‐iG to eliminate the endogenous expression of LacI and to assure that the *P_L_lacO_1_* was strictly controlled by the LacI expressed under the control of *ivbL* system (Figure 2b). In the absence of L‐Val and L‐Leu feeding, the GFP/OD_600_ value increased with time, reaching 2.81 × 10^4^ after 20‐hour cultivation in 96‐well plate. The GFP/OD_600_ values decreased after feeding various concentrations of L‐Val and L‐Leu mixture. A mixture of 5 mM L‐Val and 5 mm L‐Leu reduced the GFP/OD_600_ value to 1.90 × 10^4^, a 32.3% decrease compared to that in the absence of AA feeding.

The cells growing in test tubes could reach higher OD than in 96‐well plate, and are generally believed to be healthier. To investigate the dose effect of a single AA on *ivbL* system, we added a saturated amount of one AA (5 mM) while gradientally feeding another AA (0–5 mm) to the culture in test tubes. The AA concentration corresponding to a drop of 25% of the GFP/OD_600_ value in the absence of AA feeding was defined as the lower response limit of *ivbL* system. When L‐Val concentration was fixed to 5 mm, 0.1 mm L‐Leu feeding almost saturated the inhibition effect and the GFP/OD_600_ value dropped to 2.71 × 10^3^, 42.5% of that in the absence of L‐Leu feeding. Dose‐response correlation between L‐Leu concentration and GFP/OD_600_ value was simulated by the GraphPad Prism 8.0, showing that the lower response limit of *ivbL* system to L‐Leu was 0.018 mm while the response range was 0–0.100 mm of L‐Leu (Figure 2b). These suggested that significant *ivbL*‐dependent transcriptional attenuation could initiate at concentration of higher than or equal to 0.018 mm L‐Leu, and the L‐Leu concentration of *ivbL* system with saturated regulation effect was higher than or equal to 0.100 mm. Similarly, when L‐Leu concentration was fixed to 5, 0.4 mm of L‐Val feeding reduced GFP/OD_600_ value to 2.32 × 10^3^, 41.5% of that in the absence of L‐Val feeding. The lower response limit of *ivbL* system to L‐Val was calculated as 0.298 mm and the response range was 0–1.46 mm of L‐Val (Figure 2b). These suggested that significant *ivbL*‐dependent transcriptional attenuation could initiate at concentration of higher than or equal to 0.298 mm L‐Val, and the L‐Val concentration of *ivbL* system with saturated regulation effect was higher than or equal to 1.46 mm.

### 2.3 Design of *ivbL*‐Dependent Inducible Cascade Activation System

To adjust the repressive *ivbL* system into an inducible one, we combined *ivbL* system with LacI‐inhibited *P_L_lacO_1_* promoter to construct an AA‐concentration‐regulated inducible one‐layer cascade activation system (Figure 2c). The *gfp* reporter was placed in plasmid pS‐iL‐G under the control of *P_L_lac*O_1_, which could be inhibited by LacI being under the control of *ivbL* system. Overexpression of GFP reporter should be turned on or off in the presence or absence of AA feeding, respectively, forming a cascade system using AA as input and GFP as output. A control plasmid pS‐L‐G was a pS‐iL‐G derivative, placing the expression of LacI under the control of its native constitutive promoter *P_lacI_* instead of *ivbL* system. The regulation effect of this cascade system was verified by *E. coli* MG1655Δ*lacIZYA* strain harboring pS‐iL‐G or pS‐L‐G. In the absence of AA feeding, the background GFP/OD_600_ value from pS‐iL‐G was significantly lower than that from pS‐L‐G (Figure S3, Supporting Information), suggesting that the LacI expression under the control of *ivbL* system was higher than that under the control of *P_lacI_*. In a preliminary 96‐well plate test using *E. coli* MG1655Δ*lacIZYA* harboring pS‐iL‐G, the feeding of 5 mm L‐Val and 5 mm L‐Leu increased the 20‐hour GFP/OD_600_ value from 2.55 × 10^4^ to 3.05 × 10^4^ (Figure 2c), showing a 19.6% increase.

Feeding of 5 mm L‐Val increased the 20‐hour GFP/OD_600_ value of *E. coli* MG1655Δ*lacIZYA* harboring pS‐iL‐G in test tube from 3.67 × 10^3^ to 6.94 × 10^3^, showing an 89.1% increase (Figure 2c). This value was gradually increased to 9.87 × 10^3^ by gradually adding additional L‐Leu until 0.2 mm, which was the saturation concentration point for the response curve. On the other hand, feeding of 5 mm L‐Leu increased the 20‐hour GFP/OD_600_ value of *E. coli* MG1655Δ*lacIZYA* strain harboring pS‐iL‐G in test tube from 3.67 × 10^3^ to 4.85 × 10^3^, showing a 32.1% increase (Figure 2c). This value was gradually increased to 8.50 × 10^3^, 175% as high as the starting 4.85 × 10^3^, by gradually adding additional L‐Val until 2 mm, which was the peak point of the response curve. Taken together, our inducible cascade system could positively correlate the GFP/OD_600_ value with L‐Leu and L‐Val concentration. Besides, based on the transcriptional attenuation mechanism of *ivbL* system, we also tested the response of this inducible cascade system to other six AAs (L‐Ser, L‐Ala, L‐Thr, L‐Gly, L‐Arg, and L‐Pro) whose codons are contained in *ivbL* mRNA. Noticeably, the codon number of these six AAs are no more than five. As shown in Figure S4 (Supporting Information), this system did not have significant transcriptional attenuation effect toward any of these six AAs. These suggested the limited codon number of AA in *ivbL* mRNA could not induce the transcriptional attenuation.

We further validated the potential of this cascade system to screen out overproducers of L‐Val, the intermediate of HA production. First, plasmid MP6 was transformed into a L‐Val‐producing strain stored in our lab to establish a mutagenesis library. Addition of arabinose could induce MP6 to express special proteins which could disrupt typical repair pathways to trigger the random mutations in strain genome.^[^ ^47^ ^]^ Subsequently, the system‐associated plasmid pS‐iL‐G was transformed into the strains in the library, and the resulting GFP/OD_600_ value would correlate to the L‐Val concentration as previously described. Through initial screening and re‐screening, we obtained M6 and M7 mutants with GFP/OD_600_ values 314% and 250% as high as the one of wild type, respectively (Figure 2d). Shake flask fermentation verified that M6 strain could produce 4.53 g L^−1^ L‐Val within 24 h, 122% as high as the one of wild type (Figure 2d; Figure S5, Supporting Information). To evaluate the screening efficiency of this system, we tested whether fluorescent activated cell sorting (FACS) technique could distinguish M6 containing pS‐iL‐G from a wild type control strain containing a pS‐iL‐G derivative which has a single synonymous mutation inside the Cm marker. We mixed the two strains in 1:1 ratio, and the strains with top 1% fluorescence intensity were sorted, collected (Figure 2d), and plated. Ten single colonies were randomly collected and the Cm marker of the strain plasmids were sequenced, demonstrating that all were M6 mutants (Figure 2d).

### 2.4 AA‐ or HA‐Concentration‐Dependent Unidirectional Regulation Achieved in the CRUISE

**Figure 3:**
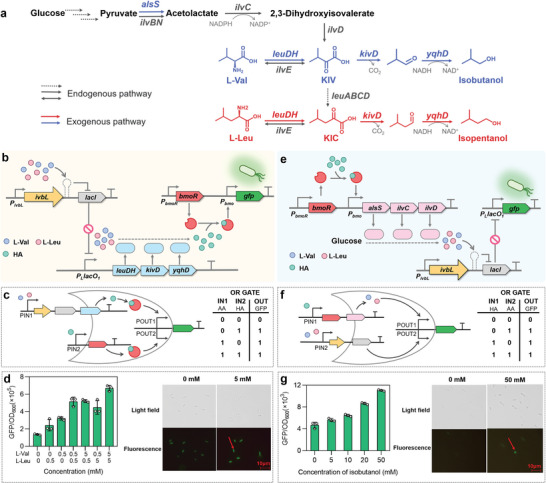
Concentration‐dependent unidirectional regulation in the CRUISE. a) The detailed biosynthetic pathways of isobutanol and isopentanol. IlvE, branched‑chain‑amino‑acid transaminase. b) Construction of the two‐layer cascade system to enable the precise inducible regulation of HA production by AA availability. c) The logic gate of “OR GATE” and the truth table of the system in (b). The input was AA or HA, and the output was GFP. d) The response effect of the system in b and the fluorescence intensity under fluorescence microscopy. e) Construction of the two‐layer cascade system to enable the precise inducible regulation of AA biosynthesis by HA availability. f) The logic gate of “OR GATE” and the truth table of the system in (e). The input was HA or AA, and the output was GFP. g) The response effect of the system in (e) and the fluorescence intensity under fluorescence microscopy. Values and error bars represent mean and SD (*n* = 3), respectively.

The detailed pathways for AA and HA biosynthesis were displayed in **Figure** **3**a. To construct the HA production module of the CRUISE, we modified the pS‐iL‐G plasmid containing the AA‐concentration‐regulated *ivbL* system by replacing the *gfp* with *leuDH*, *kivD*, and *yqhD*, forming the HA production plasmid pS‐iL‐LKY. Overexpression of LeuDH, KivD, and YqhD should be turned on or off in the presence or absence of AA feeding, respectively, forming a one‐layer cascade system using AA as input and HA production enzymes as output. Meanwhile, the *gfp* was placed under the control of HA‐concentration‐regulated BmoR system to form the indicator plasmid pS‐B‐G. Overexpression of GFP should be turned on or off in the presence or absence of HA production or feeding (Figure 3b). Introduction of plasmids pS‐iL‐LKY and pS‐B‐G into *E. coli* MG1655Δ*lacIZYA* could form a two‐layer cascade system using AA or HA as input and GFP as output, forming a logic gate of “OR GATE” (Figure 3c). Feeding of 0.5 mm L‐Val or 0.5 mm L‐Leu increased the GFP/OD_600_ values from 1.37 × 10^5^ to 3.21 × 10^5^ or 2.42 × 10^5^, respectively (Figure 3d), indicating that L‐Val had stronger regulatory effect on this system than L‐Leu. Further, the GFP/OD_600_ value continued to increase with AA concentration, reaching 6.69 × 10^5^ in the presence of 5 mm L‐Val and 5 mm L‐Leu feeding, a 389% increase compared to that in the absence of AA feeding (Figure 3d; Figure S6, Supporting Information). Fluorescence microscopy displayed that the strain could emit stronger fluorescence in the presence of AA feeding compared to that in the absence of AA feeding (Figure 3d). These results suggested that suitable concentration of AA could activate this two‐layer cascade system to achieve the precise inducible regulation of HA production by AA availability.

Next, to construct the AA biosynthesis module, we modified the pS‐B‐G plasmid containing HA‐concentration‐regulated BmoR system by replacing the *gfp* with *alsS*, *ilvC*, and *ilvD*, forming the AA biosynthesis plasmid pS‐B‐AII. Overexpression of AlsS, IlvC, and IlvD should be turned on or off in the presence or absence of HA feeding, respectively, forming a one‐layer cascade system using HA as input and AA biosynthesis enzymes as output. Plasmid pS‐iL‐G containing AA‐concentration‐regulated *ivbL* system served as an indicator plasmid. Overexpression of GFP should be turned on or off in the presence or absence of AA biosynthesis or feeding (Figure 3e). Introduction of plasmids pS‐B‐AII and pS‐iL‐G into *E. coli* MG1655Δ*lacIZYA* could form a two‐layer cascade system using HA or AA as input and GFP as output, forming a logic gate of “OR GATE” (Figure 3f). The feeding of 50 mm isobutanol increased the GFP/OD_600_ value from 4.67 × 10^3^ to 11.1 × 10^3^ (Figure 3g), showing a 137% increase. The AA concentration gradually increased with the increase of isobutanol concentration, and 0.083 g L^−1^ AA accumulated in the culture in the presence of 50 mm isobutanol feeding (Figure S7, Supporting Information). Fluorescence microscopy showed that the strain could emit distinct green fluorescence in the presence of isobutanol feeding (Figure 3g). These results suggested that suitable concentration of HA could activate this two‐layer cascade system to enable the precise inducible regulation of AA biosynthesis by HA availability, further indicating that the CRUISE could enable the mutual regulation of AA biosynthesis and HA production.

### 2.5 CRUISE‐Driven Continuous AA Biosynthesis and AA‐to‐HA Conversion

**Figure 4:**
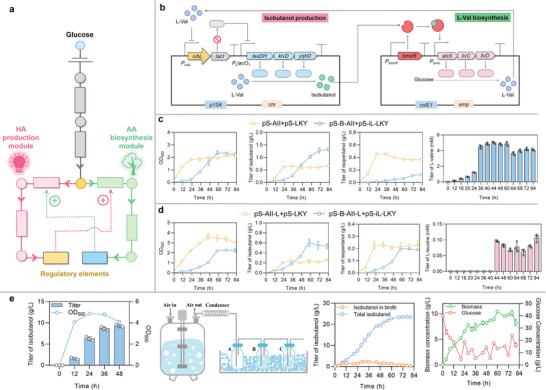
CRUISE‐driven continuous AA‐to‐HA conversion. a) The diagram of production and growth module mutually drove using the regulatory elements. b) The isobutanol production plasmid and the L‐Val biosynthesis plasmid. c) For isobutanol production, *E. coli* MG1655Δ*lacIZYA* strain harboring plasmids pS‐B‐AII and pS‐iL‐LKY was used as the experimental strain, while *E. coli* MG1655Δ*lacIZYA* strain harboring plasmids pS‐AII and pS‐LKY was used as the control strain. d) For isopentanol production, *E. coli* MG1655Δ*lacIZYA* strain harboring plasmids pS‐B‐AII‐L and pS‐iL‐LKY was used as the experimental strain, while *E. coli* MG1655Δ*lacIZYA* strain harboring plasmids pS‐AII‐L and pS‐LKY was used as the control strain. e) Fermentation of *E. coli* MG1655Δ*lacIZYA* strain harboring plasmids pS‐B‐AII and pS‐iL‐LKY in nutrient‐rich M9Y medium. Scale‐up production in a 3‐L bioreactor. Values and error bars represent mean and SD (*n* = 3), respectively.

To facilitate the CRUISE‐driven continuous AA biosynthesis and AA‐to‐HA conversion (**Figure** **4**a), we co‐transformed the AA biosynthesis plasmid pS‐B‐AII and the HA production plasmid pS‐iL‐LKY (Figure 4b) into *E. coli* MG1655Δ*lacIZYA*, creating the experimental strain. The control strain was *E. coli* MG1655Δ*lacIZYA*, which harbored constitutively expressed pS‐AII and pS‐LKY. The fermentation process was conducted in a shake flask containing yeast‐extract‐free M9NY medium. The control strain exhibited rapid growth in the first 24 h, reaching a maximum OD_600_ value of 1.85 at 24 h (Figure 4c), and maintained HA production only for the first 36 h, accumulating 0.66 g L^−1^ isobutanol at 36 h. In contrast, the experimental strain demonstrated continuous growth within 60 h, reaching a maximum OD_600_ value of 2.37 at 60 h. Correspondingly, the isobutanol titer continued to increase throughout the entire 84‐hour fermentation, reaching 1.32 g L^−1^ at 84 h, which was 97.6% higher than that of the control strain. Notably, the concentration of L‐Val reached the lower response limit of *ivbL* system between 12 h (0.151 mm) and 16 h (0.411 mm), suggesting that isobutanol production was significantly initiated during this time period. The L‐Val concentration continued to increase in the first 44 h, and maintained a stable value of 5.00 mm in the subsequent fermentation (Figure 4c), indicating that the abundant supply of generated L‐Val was rapidly converted into isobutanol via the regulation of the CRUISE, ultimately enabling the continuous AA‐to‐HA conversion.

To enhance isopentanol production, we incorporated *leuABCD* into the CRUISE by placing them under the control of *P*_bmo_ in the AA biosynthesis plasmid pS‐B‐AII, creating the plasmid pS‐B‐AII‐L. Overexpression of LeuABCD could enhance the biosynthesis of L‐Leu, a precursor for isopentanol production.^[^ ^48^ ^]^ *E. coli* MG1655Δ*lacIZYA* strain harboring pS‐B‐AII‐L and pS‐iL‐LKY was used as the experimental strain, while *E. coli* MG1655Δ*lacIZYA* strain harboring pS‐AII‐L and pS‐LKY was used as the control strain. In Figure 4d, the control strain exhibited rapid growth in the first 48 h, reaching a maximum OD_600_ value of 3.62 at 48 h. The isopentanol titer showed a similar increase trend and reached a maximum of 0.228 g L^−1^ at 36 h. In contrast, the growth of experimental strain was synchronized with the production, showing a maximum OD_600_ value of 2.20 and an isopentanol titer of 0.200 g L^−1^ at 60 h. Notably, the concentration of L‐Leu continued to increase in the first 44 h and maintained a stable value of 0.096 mm in the subsequent fermentation. However, the narrow response range of *ivbL* system to L‐Leu resulted in less L‐Leu accumulation within cells, which in turn limited the precursor supply for isopentanol production. Additionally, the production of isopentanol was also slowed down by the limited activities of LeuABCD.

Next, we tested the isobutanol production capacity of the experimental strain *E. coli* MG1655Δ*lacIZYA* harboring pS‐B‐AII and pS‐iL‐LKY in nutrient‐rich M9Y medium containing 40 g L^−1^ glucose and 4 g L^−1^ yeast extract. Within 48 h, 9.25 g L^−1^ isobutanol accumulated in the culture (Figure 4e). The potential of this engineered strain to scale up isobutanol production was verified in a 3‐L bioreactor. The schematic diagram of the fed‐batch fermentation equipment showed that the HA yielded in broth was stripped out and condensed by a condenser, and then collected into bottles A, B and C (Figure 4e). The experimental strain produced 23.5 g L^−1^ isobutanol within 80 h, meanwhile the isobutanol titer in broth was 0.208 g L^−1^. The experimental strain continued to grow until 60 h and reached a maximum biomass of 10.4 g L^−1^. The glucose concentration in the broth was maintained at ≈20 g L^−1^ to ensure sufficient carbon sources for isobutanol production. Our previous study has validated that BmoR transcriptional activation system had a linear response to 0–40 mm of isobutanol or isopentanol.^[^ ^43^ ^]^ We attempted to replace the wild‐type BmoR in the CRUISE with a mutant BmoR^T12N^, which had a response range of 0–200 mm, in order to increase isobutanol production. Fermentation results showed the isobutanol titer was not significantly enhanced (Figure S8, Supporting Information), suggesting that T12N did not obviously influence the regulatory effect of the CRUISE. This might be due to the close response intensity of wild type and T12N.^[^ ^45^ ^]^ Future work could focus on engineering BmoR with both a high output and a wide range in order to optimize the regulatory performance of the CRUISE.

### 2.6 Continuous HA Production Reflected in Transcription and Expression Levels

**Figure 5:**
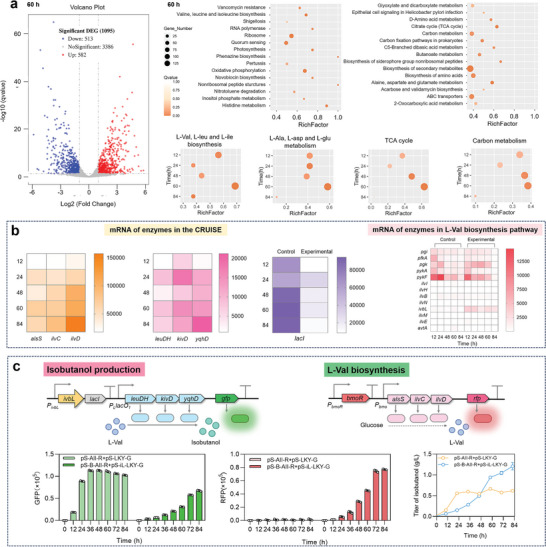
Continuous HA production reflected in transcription and expression levels. a) Differential gene analysis between the experimental strain and the control strain. *E. coli* MG1655Δ*lacIZYA* strain harboring plasmids pS‐B‐AII and pS‐iL‐LKY was used as the experimental strain, while *E. coli* MG1655Δ*lacIZYA* strain harboring plasmids pS‐AII and pS‐LKY was used as the control strain. The strains were cultured in yeast‐extract‐free M9NY medium. Volcano plot showed the number of differential genes between the experimental strain and the control strain at 60 h. The red and blue dots represented up‐ and down‐regulation, respectively. The horizontal coordinate represented the change of gene expression multiple in different samples, and the vertical coordinate represented the statistical significance of the difference in gene expression. Statistical map displayed the pathway distribution of differential genes. Rich factor refers to the ratio of the number of differential genes annotated in the pathway to the total number of annotated genes in the pathway. b) Heat map represented the differences in transcription levels of enzymes in the CRUISE and the L‐Val biosynthesis pathway. c) The amounts of GFP and RFP and the titer of isobutanol. Plasmid pS‐iL‐LKY‐G was used to characterize the expression of the enzymes in isobutanol production pathway. Plasmid pS‐B‐AII‐L was used to characterize the expression of the enzymes in L‐Val biosynthesis pathway. *E. coli* MG1655Δ*lacIZYA* strain harboring plasmids pS‐B‐AII‐R and pS‐iL‐LKY‐G was used as the experimental strain, while *E. coli* MG1655Δ*lacIZYA* harboring plasmids pS‐AII‐R and pS‐LKY‐G was used as the control strain. Values and error bars represent mean and SD (*n* = 3), respectively.

The transcription levels of many genes were significantly different between the experimental strain *E. coli* MG1655Δ*lacIZYA* harboring pS‐B‐AII and pS‐iL‐LKY and the control strain *E. coli* MG1655Δ*lacIZYA* harboring constitutively expressed pS‐AII and pS‐LKY throughout the fermentation process (**Figure** **5**a; Figure S9, Supporting Information). The differential genes were mainly concentrated in the growth‐related pathways that included TCA cycle, AA biosynthetic pathways and carbon metabolism (Figure 5a; Figure S10, Supporting Information). In the CRUISE, the transcription levels of the gens in AA biosynthesis pathway (*alsS*, *ilvC*, and *ilvD*) and the genes in HA production pathway (*leuDH*, *kivD*, and *yqhD*) increased with fermentation duration (Figure 5b), in agreement with the continuous production of isobutanol during the fermentation process. In addition, in the experimental strain the lower‐level transcription of *lacI* under the control of *ivbL* system was observed, agreeing with the higher transcription level of the polypeptide *ivbL*, as compared to the control strain (Figure 5b).

Further, a fluorescence response system was established to visualize the enzymes expression in the CRUISE (Figure 5c). GFP and RFP were selected characterize the enzymes expression in HA production pathway and AA biosynthesis pathway, respectively. *E. coli* MG1655Δ*lacIZYA* strain harboring pS‐B‐AII‐R and pS‐iL‐LKY‐G was used as the experimental strain, while *E. coli* MG1655Δ*lacIZYA* strain harboring pS‐AII‐R and pS‐LKY‐G was used as the control strain. The amounts of GFP and RFP in the experimental strain increased with fermentation duration (Figure 5c), reaching 6.75 × 10^4^ and 7.71 × 10^4^ at 84 h, respectively, indicating that the enzymes in the CRUISE were continuously expressed throughout the fermentation process. The amount of GFP in the control strain decreased slightly after reaching a maximum at 36 h, while the amount of RFP was relatively low throughout the fermentation process. Fluorescence microscopy showed that the experimental strain could emit gradually increasing red fluorescence throughout the fermentation process (Figure S11, Supporting Information).

### 2.7 Self‐Assembly‐Aided CRUISE to Enable Dominant HA Production

The biosynthetic pathways of L‐Leu and L‐Val shared some comment enzymes and pathways. The shared precursor KIV could be catalyzed by IlvE to produce L‐Val, or be subsequently catalyzed by LeuABCD and IlvE to produce L‐Leu (Figure 3a). L‐Val and L‐Leu could be sequentially catalyzed by LeuDH, KivD, and YqhD to generate isobutanol and isopentanol, respectively. The overlapping biosynthetic pathways of L‐Val and L‐Leu led to the mixed production of isobutanol and isopentanol. Isobutanol and isopentanol differ in structure by only one carbon atom, resulting in the azeotropic phenomenon during separation and purification. The dominant production of target HA could efficiently reduce the formation of azeotrope and thus could decrease the cost of separation and purification. Modification of KivD to specifically recognize KIV or KIC as substrate could avoid the mixed production, but the high structural similarity of KIV and KIC causes this modification to generate undesirable or opposite results. Base on this, the CRUISE was aided with scaffold CipA to accelerate the corresponding reactions for target precursor biosynthesis, enabling the dominant production of target HA. Scaffold‐based self‐assembly refers to a technology in which the enzyme units could aggregate into soluble or insoluble macromolecules.^[^ ^49^ ^]^ Scaffold CipA could induce the aggregation of CipA‐fused enzymes to form insoluble macromolecules.^[^ ^50^ ^]^ The generation of enzyme macromolecules could increase the local enzyme concentration within the cell. The high enzyme concentration could increase the probability of collision between the enzyme and its substrate, which in turn accelerated the efficiency of the specific reactions.^[^ ^51^ ^]^ Besides, self‐assembly of multiple enzymes which catalyzed the cascade reactions could not only reduce the probability of collision between the enzymes and the non‐target precursors, but also reduce the probability of collision between the precursors and the non‐target enzymes, which in turn decreased the by‐products biosynthesis and accelerated the target cascade reactions.^[^ ^51^ ^]^

**Figure 6:**
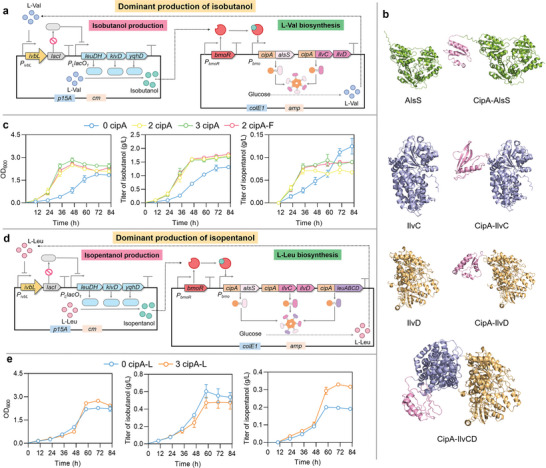
Self‐assembly‐aided CRUISE to enable the dominant AA‐to‐HA conversion. a) The dominant production of isobutanol. The isobutanol production plasmid and the L‐Val biosynthesis plasmid. b) The structures of AlsS, CipA‐AlsS, IlvC, CipA‐IlvC, IlvD, CipA‐IlvD, and CipA‐IlvC‐IlvD were simulated by AlphaFold2. Pink represented CipA. Purple represented IlvC. Orange represented IlvD. c) The dominant production of isobutanol. 0 cipA represented *E. coli* MG1655Δ*lacIZYA* strain harbored plasmids pS‐B‐AII and pS‐iL‐LKY. 2 cipA represented *E. coli* MG1655Δ*lacIZYA* strain harbored plasmids pS‐B‐CA‐CIC‐ID and pS‐iL‐LKY. CipA was individually fused to AlsS and IlvC. 3 cipA represented *E. coli* MG1655Δ*lacIZYA* strain harbored plasmids pS‐B‐CA‐CIC‐CID and pS‐iL‐LKY. CipA was individually fused to AlsS, IlvC, and IlvD. 2 cipA‐F represented *E. coli* MG1655Δ*lacIZYA* strain harbored plasmids pS‐B‐CA‐CICD and pS‐iL‐LKY. CipA was individually fused to AlsS and the fusion IlvC‐IlvD. d) The dominant production of isopentanol. The isopentanol production plasmid and the L‐Leu biosynthesis plasmid. e) The dominant production of isopentanol. 0 cipA represented *E. coli* MG1655Δ*lacIZYA* strain harbored plasmids pS‐B‐AII‐L and pS‐iL‐LKY. 3 cipA represented *E. coli* MG1655Δ*lacIZYA* strain harbored plasmids pS‐B‐CA‐CIC‐ID‐CLA‐LBCD and pS‐iL‐LKY. CipA was individually fused to AlsS, IlvC, and LeuA. Values and error bars represent mean and SD (*n* = 3), respectively.

To obtain the dominant production of isobutanol, *alsS*, *ilvC*, and *ilvD* in AA biosynthesis plasmid pS‐B‐AII were fused with *cipA* in three different types to form plasmids pS‐B‐CA‐CIC‐ID, pS‐B‐CA‐CIC‐CID and pS‐B‐CA‐CICD. The generated enzymes CipA‐AlsS, CipA‐IlvC, and CipA‐IlvD could aggregate to speed up the biosynthesis of KIV, which was then converted into isobutanol (**Figure** **6**a). The structure simulation and enzyme assays demonstrated that CipA did not obviously change the folding (Figure 6b) and the activity of AlsS, IlvC or IlvD (Figure S12, Supporting Information). These three plasmids were individually co‐transformed with HA production plasmid pS‐iL‐LKY into *E. coli* MG1655Δ*lacIZYA* to generate the experimental strains for fermentation in M9NY medium, while *E. coli* MG1655Δ*lacIZYA* strain harboring pS‐B‐AII and pS‐iL‐LKY was used as the control strain. As shown in Figure 6c, the experimental strain containing the individual fusion of CipA with AlsS, IlvC, and IlvD reached a maximum OD_600_ value of 2.84 at 48 h, a 244% higher value when compared with the control strain. The experimental strains rapidly accumulated isobutanol in the first 48 hours. Significantly, the experimental strain containing the individual fusion of CipA with AlsS and IlvCD produced 1.60 g L^−1^ isobutanol within 48 h, which was 129% higher than that of the control strain. No significant difference in isobutanol titer was appeared between the experimental strains with different scaffold‐mediated fusion. For by‐product isopentanol, the control strain reached a highest titer of 0.125 g L^−1^ at 84 h, while the experimental strain containing the individual fusion of CipA with AlsS and IlvCD only produced 0.090 g L^−1^ isopentanol. Introduction of scaffold into the CRUISE not only significantly improved the production rate, but also ensured the yield of isobutanol.

To obtain the dominant production of isopentanol, *alsS*, *ilvC*, and *leuA* in pS‐B‐AII‐L were fused with *cipA*, respectively, forming plasmid pS‐B‐CA‐CIC‐ID‐CLA‐LBCD. This plasmid was co‐transformed with pS‐iL‐LKY into *E. coli* MG1655Δ*lacIZYA* to generate experimental strain, while *E. coli* MG1655Δ*lacIZYA* strain harboring pS‐B‐AII‐L and pS‐iL‐LKY was used as the control strain. Self‐assembly of AlsS, IlvC and LeuA could accelerate the biosynthesis of KIC, which was then converted into isopentanol (Figure 6d). Similarly, CipA did not obviously change the folding of LeuA (Figure S13, Supporting Information). In the presence of CipA in the CRUISE, the isopentanol titer reached a maximum of 0.330 g L^−1^ at 72 h (Figure 6e), 67.6% higher than that of the control strain. The control strain produced 0.605 g L^−1^ isobutanol within 60 h, while the experimental strain produced 0.473 g L^−1^ isobutanol, showing a decrease of 21.7%. These results suggested that with the assistance of self‐assembly scaffold, the CRUISE could dominant the production of target HA while significantly increasing production efficiency.

### 2.8 Utilization of HA‐Tolerant Strain for Efficient Isobutanol Production

**Figure 7:**
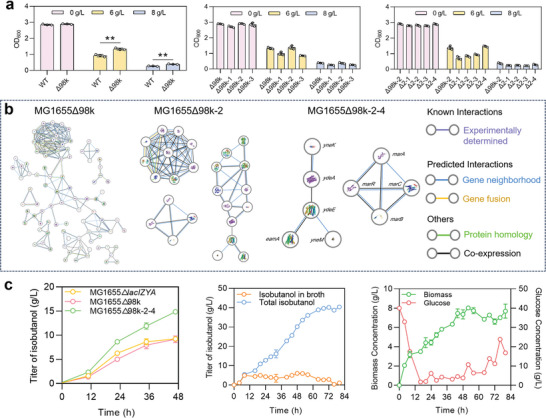
Utilization of HA‐tolerant strain for efficient isobutanol production. a) Verification of the isobutanol tolerance of different knockout strains and confirmation of the knockout region that determined the tolerance. b) Analysis of the correlation between the genes in the knockout region using PPI network from STRING database. c) The shake‐flask production of isobutanol using M9Y medium and the scale‐up production of isobutanol in a 3‐L bioreactor. For shake‐flask fermentation, *E. coli* MG1655Δ*lacIZYA* strain harboring plasmids pS‐B‐AII and pS‐iL‐LKY, *E. coli* MG1655Δ98k strain harboring plasmids pS‐B‐AII and pS‐iL‐LKY, and *E. coli* MG1655Δ98k‐2‐4 strain harboring plasmids pS‐B‐AII and pS‐iL‐LKY were used. For scale‐up fermentation, *E. coli* MG1655Δ98k‐2‐4 strain harboring plasmids pS‐B‐CA‐CICD and pS‐iL‐LKY was used. CipA was individually fused to AlsS and the fusion IlvC‐IlvD. Values and error bars represent mean and SD (*n* = 3), respectively. ^∗^ *P* < 0.1, ^∗∗^ *P* < 0.01, as determined by two‐tailed *t*‐test.

We investigated the toxic effects of HA on the cells. Different concentrations of isobutanol and isopentanol were individually fed to the medium containing strain *E. coli* MG1655. As shown in Figure S14 (Supporting Information), in the presence of 0–2 g L^−1^ isobutanol or 0–1 g L^−1^ isopentanol *E. coli* MG1655 strain had comparable OD_600_ values to those in the absence of HA, indicating that the low concentrations of HA were only slightly toxic to the cells. However, HA higher than 4 g L^−1^ significantly reduced the OD_600_ values of *E. coli* MG1655. Wild‐type chassis host, which was not evolved for exogenous HA production and less tolerant to HA, has limited production efficiency of HA. Base on this, we applied HA‐tolerant strain to demonstrate the HA production capacity of the CRUISE. We first screened an unpublished lab‐stored library of *E. coli* MG1655‐derived strains with different genomic large‐fragment deletions ranging from 14 to 143 kb, and obtained an isobutanol‐tolerant *E. coli* MG1655Δ98k with a 98 kb deletion. This strain demonstrated a 44.1% higher OD_600_ value than wild type against a background of 6 g L^−1^ isobutanol (**Figure** **7**a). Under microscope, the cell length of *E. coli* MG1655Δ98k was significantly longer than that of *E. coli* MG1655 (Figure S15, Supporting Information). Next, to identify specific genes that improved isobutanol tolerance, we divided the 98 kb deletion fragment into three regions in a direction of 5′ to 3′ and individually deleted these regions. *E. coli* MG1655Δ98k‐2, which was generated by knocking out the 2nd region, showed significant isobutanol tolerance and had an OD_600_ value 49.5% higher than that of wild type against a background of 6 g L^−1^ isobutanol. We further divided the 2nd region into four subregions and individually deleted these subregions. *E. coli* MG1655 with a deletion of the 4th subregion (*E. coli* MG1655Δ98k‐2‐4) had significant isobutanol tolerance. Under the background of 6 and 8 g L^−1^ isobutanol, the OD_600_ values of this strain were 58.3% and 8.00% higher than those of wild type, respectively. The cell length of *E. coli* MG1655Δ98k‐2‐4 was similar to that of *E. coli* MG1655 (Figure S15, Supporting Information). The 4th subregion contained *yneK*, *yneM*, *ydeA*, *ydeE*, *eamA*, *marA*, *marB*, *marC*, and *marR* (Figure 7b), which are related to the substances transport and the resistance of the strain to harsh conditions. We speculated that knockout of these genes could dilute isobutanol concentration within the cells to generate the isobutanol tolerance.

Subsequently, the AA biosynthesis plasmid pS‐B‐AII and the HA production pS‐iL‐LKY were co‐transformed into *E. coli* MG1655Δ98k‐2‐4 to establish the CRUISE for continuous production of isobutanol. The engineered *E. coli* MG1655Δ98k‐2‐4 could produce 14.9 g L^−1^ isobutanol within 48 h in M9Y medium (Figure 7c), 61.1% higher than that of *E. coli* MG1655Δ*lacZYA* strain harboring pS‐B‐AII and pS‐iL‐LKY. Subsequently, we scaled up the isobutanol production of this strain in a bioreactor. The scaffold‐aided plasmid pS‐B‐CA‐CIC‐ID was co‐transformed with pS‐iL‐LKY into *E. coli* MG1655Δ98k‐2‐4 to generate the experimental strain for fed‐batch fermentation. As shown in Figure 7c, the experimental strain produced isobutanol rapidly in the first 8 h, reaching a 4.91‐g L^−1^ broth titer and a 5.52‐g L^−1^ total titer at 8 h. After this time point, the total isobutanol titer continued to increase, reaching a maximum of 40.4 g L^−1^ at 80 h, a 71.9% higher value when compared with the HA‐intolerant *E. coli* MG1655Δ*lacZYA* strain harboring pS‐B‐AII and pS‐iL‐LKY. Besides, the experimental strain had a maximum biomass of 8.02 g L^−1^ at 52 h. These results suggested that utilization of HA‐tolerant strain could boost the regulatory performance of the self‐assembly‐aided CRUISE to accelerate the scale‐up production of HA.

## 3 Discussion

In the inherent production mode where growth and production are independent of each other, the inappropriate match between microbes and exogenous production pathways leads to uneven resource allocation, resulting in the dilemma that growth and production could not be maintained for a long time. In this study, we developed a CRUISE based on the sensing of AA and HA concentrations. This system established a close connection between endogenous AA biosynthesis and exogenous HA production, further improving the adaptability of cells to HA production. In this system, the microbes were promoted to intelligently balance the allocation of resources and form a GiPiG production mode in which growth and production drove each other, enabling the continue AA‐to‐HA conversion through the fermentation process and effectively avoiding the waste of resources in non‐target production pathways. In the biosynthetic pathway from glucose to HAs, i.e., isobutanol and isopentanol, the essential AAs, i.e., L‐Leu and L‐Val are essential intermediates and their biosynthetic pathways could not be deleted in the cells due to the growth needs. The crucial enzymes for converting AA to HA have substrate diversity, which led to the partial overlap of isobutanol and isopentanol biosynthetic pathways and further resulted in the mixed production of isobutanol and isopentanol. Base on this, we developed self‐assembly‐aided CRUISE so that the cell resources were tilting toward achieving directed AA‐to‐HA conversion. Further, an HA‐tolerant strain was isolated to boost the production capacity of the self‐assembly‐aided CRUISE.

For the CRUISE‐driven continuous conversion of L‐Val to isobutanol, the high concentration of L‐Val ensured sufficient precursor for continue isobutanol production throughout the fermentation process. The enzymes in L‐Val biosynthetic pathway except AlsS, IlvC, and IlvD had comparable transcription levels in the experimental and control strains (Figure 5a). However, we did not detect significant accumulation of L‐Val in the control strain. The experimental strain had higher L‐Val production capacity than the control strain (Figure 4c), indicating that the crucial enzymes for the L‐Val biosynthesis were AlsS, IlvC, and IlvD involved in the CRUISE. To develop self‐assembly‐aided CRUISE, we used AlphaFold2 to model the structures of CipA‐AlsS, CipA‐IlvC, CipA‐IlvD, CipA‐IlvCD, and CipA‐LeuA, and analyzed the effects of CipA on the structures of AlsS, IlvC, and IlvD. Based on the structures of CipA (AF‐Q7N6H4‐F1), AlsS (AF‐Q04789‐F1), IlvC (AF‐P05793‐F1), IlvD (AF‐P05791‐F1) and LeuA (AF‐P09151‐F1) from UniProt, we assembled the fusion state of CipA‐AlsS, CipA‐IlvC, CipA‐IlvD, CipA‐IlvC‐IlvD, and CipA‐LeuA, and confirmed that the structures of the fusion enzymes were similar to that of the CipA‐free structures (Figure 6b; Figure S13, Supporting Information). These results suggested that CipA did not change the folding of AlsS, IlvC, or IlvD, suggesting that CipA did not influence the catalytic mechanisms of AlsS, IlvC, and IlvD and the increased HA production by CipA fusion was likely due to the increased catalytic efficiencies induced by aggregation effects.

For the HA‐tolerant strain *E. coli* MG1655Δ98k‐2‐4, the genes *yneK*, *yneM*, *ydeA*, *ydeE*, *eamA*, *marA*, *marB*, *marC*, and *marR* were knocked out. YneM, YdeA, YdeE, and EamA are closely related and are involved in the transport of Mg^2+^, L‐arabinose, dipeptide, and cysteine/O‐acetylserine, respectively (Figure 6b). YdeA and YdeE belong to the major facilitator superfamily (MFS) of transporters.^[^ ^52^, ^53^ ^]^ YneM is inner membrane protein and EamA is integral membrane protein.^[^ ^54^, ^55^ ^]^ MarA, MarB, MarC, and MarR are also distributed on the cell membrane and are closely related.^[^ ^56^ ^]^ These four proteins participate in regulating the expression of several genes involved in resistance to antibiotics, oxidative stress, and organic solvents. Notably, MarC is an inner membrane protein with six predicted transmembrane domains, and has been proved that deletion of *marC* could increase the tolerance of cells to isobutanol.^[^ ^57^ ^]^ We speculated that knockout of these genes significantly affected the substances transport and the resistance of the strain to harsh conditions, which in turn increased the tolerance of the strain to HA.

The design of the CRUISE could be applied to establish other dynamic regulation systems for the production of other L‐Val or L‐Leu‐derived compounds such as 4‐methy‐1‐pentanol, 2‐methyl‐1,3‐propanediol (MPO), 2‐methyl‐1,4‐butanediol (2‐M‐1,4‐BDO) and isopentyldiol (IPDO).^[^ ^58^ ^]^ Any of the components in the system could be replaced with the relevant components for other compounds biosynthesis in order to build the corresponding system. Notably, mining and modification of the TFs‐based biosensors for responding to L‐Val or L‐Leu‐derived compounds is necessary for constructing functional dynamic regulation systems. Based on this, a highly responsive mutant BmoR^V311A^ reported in our previous study^[^ ^45^ ^]^ was used to construct the regulation system for responding to MPO, a derivative of L‐Val (Figure S16, Supporting Information). BmoR^V311A^ had a significant linear response to 0–100 mm MPO (Figure S17, Supporting Information). Enzymes MfL, AaD, KivD, and YqhD in MPO biosynthetic pathway were introduced into the CRUISE to replace the enzymes in isobutanol biosynthetic pathway. Due to the limited activity of MfL and AaD, we could only detect the accumulation of by‐product isobutanol, but not the final product MPO. Whether the enzymes in production pathway have high activities is crucial for establishing high‐performance CRUISE.

In the CRUISE, the natural regulatory elements are suboptimal for production, i.e., the identification pocket is not specific to SM, the key residue side chain is not fitted, and the sensitivity is low, etc. These performances could not meet the requirements of the CRUISE for start‐up speed and accuracy, resulting in a waste of the limited resources and thus affecting the efficient production. For the system established in this study, the more sensitive the *ivbL* system, the faster the start‐up speed of the CRUISE. This could activate HA production at low concentration of AA, so that the cells could allocate resources to HA production in time in the initial stage of fermentation. Based on this, we tried to semi‐rationally engineer *ivbL* for the first time, and established a visual high‐throughput screening system (Figure S18, Supporting Information). Two highly responsive *ivbL* mutants were obtained (Figure S19, Supporting Information). The sequences of *ivbL* mutants were displayed in supplementary Table S2 (Supporting Information). The response effects of these two highly responsive *ivbL* mutants toward L‐Val or L‐Leu were tested via kinetic experiments, and the highly responsive mutants reached the maximum response value within a short time (≈4 h) (Figure S20, Supporting Information). We then introduced *ivbL* mutants into the two‐layer cascade system of regulating HA production by AA availability to verify the response effect of the mutants (Figure S21, Supporting Information). In the absence of AA feeding, the strains with *ivbL* mutants emitted distinct fluorescence, brighter than the strain with wild type (Figure S22a, Supporting Information). In the presence of 0.5 mm AA feeding, the strains with *ivbL* mutants or wild type emitted similar fluorescence. In the presence of low concentrations of AA (0‐0.01 mm) feeding, the strains with *ivbL* mutants had higher response values than the strain with wild type. In the presence of high concentrations of AA (0.05–0.5 mm) feeding, the strains with *ivbL* mutants had lower response values than the strain with wild type (Figure S22b, Supporting Information). After that, we introduced *ivbL* mutants into the two‐layer cascade system of regulating AA biosynthesis by HA availability (Figure S23, Supporting Information). The results in Figure S24a,b (Supporting Information) showed that the strains with *ivbL* mutants displayed stronger fluorescence responses than the strain with wild type in the presence of 50 mm isobutanol feeding. Meanwhile, an increase in isobutanol concentration could enhance the fluorescence output of the strains with *ivbL* mutants. Subsequently, we introduced these two highly responsive *ivbL* mutants into the CRUISE, but the yield of HA was not significantly improved (Figure S25, Supporting Information). This might be because microbes quickly, but temporarily, devoted large amounts of resources to expressing the HA production pathway when using highly responsive *ivbL* mutants for dynamic regulation, while the supply of precursors was not sufficient, resulting in the difficulty for the long‐term HA production.

We also rationally designed *ivbL* to obtain some special mutants. First, we replaced all L‐Leu codons in *ivbL* with L‐Ser codons in an attempt to generate a mutant that specifically responded to L‐Val. We used structural simulation to test the secondary structures of this mutant when L‐Val was sufficient and deficient, respectively. This mutant could form a terminator loop only when L‐Val was sufficient (Figure S26, Supporting Information). The AA‐concentration‐regulated one‐layer cascade activation system was used to verify the response effect of this mutant toward AA. As shown in Figure S27 (Supporting Information), this mutant could respond only to L‐Val, but not L‐Leu, while the response value was slightly lower than that of wild‐type *ivbL*. Next, introduction of this mutant in the CRUISE enabled an accumulation of 0.914 g L^−1^ isobutanol within 72 h (Figure S28, Supporting Information), which was slightly lower than that of the CRUISE containing wild‐type *ivbL*. This might be due to the weak response of this mutant to L‐Val. Subsequently, we replaced all L‐Val codons in *ivbL* with L‐Ser codons, trying to generate a mutant that specifically responded to L‐Leu. This mutant did not show a significant linear response to L‐Leu. After that, we tried to replace all L‐Val and L‐Leu codons in *ivbL* with the codons of other eighteen AAs, in an attempt to obtain *ivbL* mutants that respond to other AAs. The secondary structure simulation showed the mutants containing the codons of one of the eleven AAs (L‐Thr, L‐Pro, L‐Ile, L‐Tyr, L‐Asp, L‐Asn, L‐Cys, L‐Arg, L‐Ser, L‐Gln, and L‐His) could form a terminator loop only when AA was sufficient (Figures S29–S33, Supporting Information). The validation experiments showed that only the mutant with L‐Thr codons substitution had a linear response of 0–10 mm to L‐Thr and the mutant with L‐Pro codons substitution had a linear response of 0–10 mm to L‐Pro (Figure S34, Supporting Information). The *ivbL* mutants containing the codons of one of other seven AAs (L‐Ala, L‐Glu, L‐glycine, L‐Trp, L‐Lys, L‐Phe, and L‐Met) could not form a terminator loop when AA was sufficient (Figure S35, Supporting Information). These results suggested that developing other AAs concentration‐dependent *ivbL* mutants required to modify other regions besides those containing L‐Val and L‐Leu codons.

In addition, the SM‐specific BmoR could enable precise regulation by the CRUISE to accurately allocate resources to the directed AA‐to‐HA conversion. The SM recognition and binding region of BmoR is a key region that determines its SM species. Modifying this region might allow rapid access to HA‐specific BmoR. Random mutagenesis could be carried out on HA binding regions. BmoR mutants specific for isobutanol or isopentanol could be screened and used for precise regulation of the systems to produce target HA. Besides, *de novo* design of regulatory elements is an alternative way to create new regulatory elements. The design process requires a deep understanding of the underlying regulatory mechanisms and the interplay between TFs and DNA sequences. So far, machine learning and deep learning techniques have emerged as powerful tools in the field of protein prediction, modification, and redesign.^[^ ^59^, ^60^ ^]^ These AI techniques leverage large‐scale datasets and complex algorithms to extract patterns, learn from existing knowledge, and make predictions or generate novel solutions. These AI techniques have the potential to greatly accelerate the design and modification of novel regulatory elements. By combining these AI techniques with traditional protein engineering approaches, the novel regulatory elements with desired functionalities could be rapidly generated and optimized, enabling precise regulation on gene expression.

## 4 Experimental Section

### Medium, Strains, and Plasmids

*Escherichia coli* DH5α was used as host for plasmid construction in this study, while *E. coli* MG1655Δ*lacIZYA* derived from *E. coli* MG1655 by deleting *lacIZYA* genes was used as host for fluorescence characterization and HA production. LB medium contains 10 g L^−1^ tryptone, 5 g L^−1^ yeast extract, and 10 g L^−1^ NaCl was used for inoculation and plasmid construction. M9NY contains 10 g L^−1^ glucose, 6 g L^−1^ Na_2_HPO_4_, 3 g L^−1^ KH_2_PO_4_, 1 g L^−1^ NH_4_Cl, 0.5 g L^−1^ NaCl, 1 mm MgSO_4_, 0.1 mm CaCl_2_ and 10 mg L^−1^ VB_1_ was used for the fluorescence characterization of the units in dynamic regulation system and for the microbial production of HA. M9Y contains 40 g L^−1^ glucose, 4 g L^−1^ yeast extract, 6 g L^−1^ Na_2_HPO_4_, 3 g L^−1^ KH_2_PO_4_, 1 g L^−1^ NH_4_Cl, 0.5 g L^−1^ NaCl, 1 mm MgSO_4_, 0.1 mm CaCl_2_, and 10 mg L^−1^ VB_1_ was used for the microbial production of HA. The details of strains and plasmids were described in Table S3 (Supporting Information).

### Plasmid Construction

To investigate the regulatory effect of AA on *ivbL* transcriptional attenuation system, the *ivbL* operon sequence was amplified from *E. coli* MG1655 genome and gene *gfp* (accession number: AAX07425.1) was synthesized by OE‐PCR. Gene *gfp* were assembled downstream of *ivbL* operon by Gibson Assembly to generate plasmid pS‐iG. The *ivbL* operon, *lacI* which was amplified from pCS97,^[^ ^46^ ^]^ and the *P_L_lacO_1_*‐*gfp* operon were sequentially assembled to generate plasmid pS‐iL‐G. The *P_L_lacO_1_*‐*gfp* operon was obtained by OE‐PCR of *P_L_lacO_1_* promoter and *gfp*. To construct the production plasmid, genes *leuDH, kivD* and *yqhD* were amplified from pCS97^[^ ^46^ ^]^ and then assembled into pS‐iL‐G by replacing the *gfp*, resulting in plasmid pS‐iL‐LKY. The *gfp* was constructed downstream of *leuDH, kivD*, and *yqhD* in pS‐iL‐LKY to generate plasmid pS‐iL‐LKY‐G, which was used for detection of the expression of production‐related enzymes. To create the growth‐related plasmid, the genes *alsS, ilvC*, and *ilvD* were amplified from pSA69^[^ ^41^ ^]^ and ligated with the *P_bmoR_*‐*bmoR‐P_bmo_* from pYH1^[^ ^43^ ^]^ via Gibson Assembly to result in plasmid pS‐B‐AII. To enhance the production of L‐Leu, the gene cluster *leuABCD* amplified from *E. coli* MG1655 genome was assembled downstream of *alsS, ilvC*, and *ilvD* in pS‐B‐AII to generate plasmid pS‐B‐AII‐L. Correspondingly, the gene cluster *leuABCD* was assembled downstream of *alsS, ilvC*, and *ilvD* in pS‐AII to generate pS‐AII‐L. To detect the expression of growth‐related enzymes, the *rfp* was assembled downstream of *alsS, ilvC*, and *ilvD* in pS‐B‐AII to generate plasmid pS‐B‐AII‐R. The *rfp* was assembled downstream of *alsS, ilvC*, and *ilvD* in pS‐AII to generate pS‐AII‐R. The *gfp* was assembled downstream of *leuDH, kivD*, and *yqhD* in pS‐LKY to generate pS‐LKY‐G. In order to achieve the dominant production of isobutanol, *Photorhabdus luminescens cipA* was individually fused N‐terminal of the *alsS* and *ilvC* in pS‐B‐AII to result in plasmid pS‐B‐CA‐CIC‐ID. In addition, *cipA* was individually fused N‐terminal of the *alsS*, *ilvC*, and *ilvD* in pS‐B‐AII to result in plasmid pS‐B‐CA‐CIC‐CID. Fusing *cipA* and alsS, and fusing *cipA*, *ilvC*, and *ilvD* in the plasmid pS‐B‐AII resulted in plasmid pS‐B‐CA‐CICD. For the dominant production of isopentanol, *cipA* was individually fused N‐terminal of the *alsS*, *ilvC*, and *leuA* in pS‐B‐AII‐L to result in plasmid pS‐B‐CA‐CIC‐ID‐CLA‐LBCD. All the plasmids were sequenced by GENEWIZ company. The overexpression genes sequences were displayed in supplementary Table S4 (Supporting Information).

### Fluorescence Kinetic Detection

To characterize the *ivbL* transcriptional attenuation system and identify the trend of the response intensity of *ivbL* system to L‐Val and L‐Leu, the corresponding *E. coli* transformants were cultured in 3 mL LB medium with appropriate antibiotics at 37 °C at 220 rpm for 8 h. Then 4 µL cultures were transferred into a black 96‐well plate with clear bottom (BRAND plates) containing 200 µL of M9NY medium with various concentrations of AA (0–5 mm) and appropriate antibiotics. The plate was incubated in the plate reader (BioTek Cytation 3) with continuous shaking at 30 °C for 20 h. The OD_600_ values and fluorescent intensities were quantified every 30 min. The excitation and emission wavelengths of green fluorescent were set at 470 and 510 nm, respectively.

### Fluorescence Assay

Single colonies were cultivated in 3 mL LB medium with appropriate antibiotics for 8 h at 37 °C at 220 rpm. Each sample of culture was washed with M9NY medium to remove the residual LB and then inoculated into 3 mL fresh M9NY medium with a final OD_600_ value of 0.1. To measure the response of *ivbL* transcriptional attenuation system to L‐Val and L‐Leu, the AA concentrations in the culture varied from 0 to 5 mm. To measure the response of BmoR transcriptional activation to HA, the HA concentrations in the culture varied from 0 to 50 mm. The cultures were then incubated at 30 °C, 220 rpm for 20 h. Microplate reader was used to detect the OD_600_ value and GFP fluorescence.

### Fermentation Verification in Shake Flask

Single colonies of the strain harboring growth and production plasmids were pre‐inoculated into 3 mL LB medium with associated antibiotics for 8 h at 37 °C, 220 rpm. Then, 200 µL culture was inoculated into 20 mL M9NY or M9Y in 250 mL screw cap conical flask and then left at 30 °C in a shaker at 220 rpm. Samples were taken every 12 h for OD_600_ measurement and HA concentration detection. HA was quantified by Agilent 6890 GC chromatograph equipped with flame ionization detector (Agilent Technologies, CA, USA). The analysis method was performed as described in the previous study.^[^ ^61^ ^]^ For red fluorescent detection, the excitation and emission wavelengths were set at 532 and 588 nm, respectively.

### Fed‐Batch Fermentation

To scale up the production of the strain containing the dynamic regulation system, the fermentation was performed in a 3‐L bioreactor with 1 L working volume. The medium containing 40 g L^−1^ glucose, 3 g L^−1^ (NH_4_)_2_SO_4_, 14.6 g L^−1^ K_2_HPO_4_, 4 g L^−1^ KH_2_PO_4_, 2.2 g L^−1^ sodium citrate, 8 g L^−1^ yeast extract, 1.25 g L^−1^ MgSO_4_·7H_2_O, 0.1 g L^−1^ ampicillin, 0.025 g L^−1^ chloromycetin and 1 mL L^−1^ trace metal solution was used for fermentation. Trace metal solution contained 14.1 g EDTA, 2.5 g CoCl_2_·6H_2_O, 15 g MnCl_2_·4H_2_O, 1.5 g CuCl_2_·2H_2_O, 3 g H_3_BO_3_, 2.1 g Na_2_MoO_4_·2H_2_O, 33.8 g Zn (CH_3_COO)_2_·2H_2_O, and 80 g FeCl_3_·6H_2_O per liter. During the cultivation period, 1.5 L stock solution containing 600 g L^−1^ glucose, 1.25 g L^−1^ MgSO4·7H_2_O, 0.1 g L^−1^ ampicillin, 0.025 g L^−1^ chloromycetin was used for fed‐batch fermentation. Single colonies were pre‐inoculated into 3 mL LB medium with associated antibiotics for 8 h at 37 °C at 220 rpm. Then, 250 µL culture was inoculated into 25 mL LB with associated antibiotics and then was left at 37 °C at 220 rpm to generate the seed culture with an OD_600_ value of 0.8. 100 mL seed culture was inoculated into 1 L medium in fermenter and then grew at 37 °C with 1 vvm of air flow rate and 600 rpm of stirrer speed for 4 h. The temperature was then changed to 30 °C for the expression of growth and production‐related enzymes. The pH was controlled at 6.8 by automatic addition of ammonia solution (25%). After incubating at 30 °C for 12 h, the air flow rate was increased from 1 to 3 vvm in order to strip out HA from the broth. The evaporated HA was condensed by a condenser and subsequently the generated liquid HA flowed into collection bottle A. The residual uncondensed HA was collected into bottle B and C which contained 800 mL water. The samples were taken to determinate the biomass, glucose concentration, and HA titers. The total isobutanol titer was calculated as the sum of isobutanol in bottle A, B, C, and in broth.

### Knockout of *lacIZYA* in *E. coli* MG1655

The plasmid p15A‐PBAD‐Cas9‐PT5‐Redγβα constructed in the previous study^[^ ^62^ ^]^ was transformed into *E. coli* MG1655 and then cultured in LB plate containing kanamycin. Temperature‐sensitive plasmid pHCY26D‐*lacIZYA* was constructed to express specific sgRNA which was designed on the website http://chopchop.cbu.uib.no/#. Plasmid pHCY26D‐*lacIZYA* was then transformed into *E. coli* MG1655 harboring p15A‐PBAD‐Cas9‐PT5‐Redγβα and cultivated on LB plate with ampicillin, kanamycin, and glucose for 30 °C. Several single colonies of *E. coli* MG1655 harboring p15A‐PBAD‐Cas9‐PT5‐Redγβα and pHCY26D‐*lacIZYA* were inoculated into 2 mL LB medium, and then cultivated at 30 °C for 2 h. Then, 0.1 vol.% of stock solution containing 0.1 g L^−1^ ampicillin, 0.05 g L^−1^ kanamycin, and 1 mm IPTG was added into the culture. After 1 h, 20 mm L‐arabinose was added into the culture and cultivated for another 3 h at 30 °C. Subsequently, the culture was diluted and cultured in LB plate containing ampicillin, kanamycin, and L‐arabinose at 30 °C for 12 h. Positive knockout strains were verified by colony PCR and DNA sequencing. Positive strains were then cultivated in antibiotic‐free LB medium at 37 °C for 16 h to lose the plasmids pSC101‐PBAD‐sgRNA‐Donor and p15A‐PBAD‐Cas9‐PT5‐Redγβα. The colonies that grew on the plate were the strains without plasmids. This method was utilized to obtain the strain *E. coli* MG1655Δ*lacIZYA*. The corresponding plasmid for knockout, and the *sgRNA* and *donor* sequences were supplied in Tables S3 and S5 (Supporting Information), respectively.

### Construction of the Mutagenesis Library for L‐Val‐Producing Strain

Plasmid MP6 for mutagenesis was bought from the website https://www.addgene.org and was transformed into L‐Val‐producing strain, which was then cultured in LB plate containing 25 mm glucose at 37 °C for 12 h. Subsequently, single colonies were inoculated into LB medium containing 40 mg mL^−1^ chloramphenicol and 25 mm glucose for 12 h to obtain seed culture. Seed culture was then inoculated into LB medium containing 40 mg mL^−1^ chloramphenicol and 25 mm glucose with a ratio of 0.1 vol.%. After incubating the culture at 37 °C for 3 h, 25 mm arabinose was added into the medium to induce the expression of enzymes in plasmid MP6. The induction was performed at 37 °C for 24 h. The generated culture was then incubated into LB medium containing 100 mm glucose with a ratio of 1 vol.% and cultivated at 37 °C for 12 h to generate the mutagenesis library.

### Screening of the Mutagenesis Library

The inducible cascade regulatory system‐related plasmid pS‐iL‐G was transformed into the strains included in the mutagenesis library to enable the screening of L‐Val overproducers through detection of GFP/OD_600_ value. Single colonies were inoculated into LB medium containing chloramphenicol and left at 37 °C for 12 h to generate seed culture. Subsequently, seed culture was inoculated into LB medium containing chloramphenicol in a 96‐deep well plate with a ratio of 5 vol.% and incubated at 30 °C for 24 h. Microplate reader was used to detect OD_600_ value and GFP. The strains with higher GFP/OD_600_ values than wild‐type strain were selected as candidates. To evaluate the screening efficiency of the inducible cascade regulatory system, 1 µL wild type strain harboring pS‐iL‐G and 1 µL mutated strain harboring pS‐iL‐G were mixed and then added to 2 mL PBS buffer for FACS analysis. For the plasmid pS‐iL‐G in wild type, the marker *Cm* has a synonymous mutation from ggt to ggc in Gly128 codon.

### Derivatization of L‐Val and L‐Leu

Acetonitrile was used as solvent to prepare 1 mm triethylamine and 0.2 m phenyl isothiocyanate (PITC). The samples were centrifuged at 12 000 rpm and the supernatant was retained. 200 µL supernatant was then mixed with 100 µL 1 mm triethylamine and 100 µL 0.2 m PITC to induce the derivatization reaction for 1 h at room temperature. Subsequently, 400 µL n‐hexane was added into the mixture and vortexed for 10 s to extract the derivatized L‐Val or L‐Leu. The derivatized samples were then analyzed by uHPLC.

### uHPLC Analysis

The derivatized samples were analyzed and quantified by uHPLC (Agilent Technologies 1290 Infinity II). Solvent A was ddH_2_O with 0.1% formic acid, and solvent C was 100% acetonitrile. The column temperature was set to 30 °C and the flow rate of solvent was 1 mL min^−1^ with gradient concentrations: 5–98% solvent C for 6 min, 98% solvent C for 5 min, and 98‐5% solvent C for 4 min. Quantification of derivatized L‐Val was based on the peak area at absorbance of 254 nm.

### Pretreatment of the Samples for Transcriptome Analysis

The pre‐culture of the experimental strain *E. coli* MG1655Δ*lacIZYA* harboring plasmids pS‐iL‐LKY and pS‐B‐AII or the control strain *E. coli* MG1655Δ*lacIZYA* harboring plasmids pS‐AII and pS‐LKY were incubated into 20 mL M9NY medium for the fermentation in shake flask. The samples were taken at 12, 24, 48, 60, and 84 h, respectively. 20 mL samples at each time point were centrifuged at 12 000 rpm for 10 min. The supernatant was discarded, and the cell precipitation were retained for transcriptome sequencing by Azenta Life Sciences Company.

### Identification of the Crucial Proteins for Isobutanol Tolerance

An unpublished library of *E. coli* MG1655‐derived strains with different genomic large‐fragment deletions ranging from 14 to 143 kb was constructed and stored in the laboratory. The library was screened through addition of 0, 6 and 8 g L^−1^ isobutanol, respectively. The pre‐culture was transferred to fresh LB medium with a ratio of 1 vol.%. 0, 6 and 8 g L^−1^ isobutanol were individually added to the culture and then incubated at 37 °C for 24 h. Microplate reader was used to detect OD_600_ value. An isobutanol‐tolerant *E. coli* MG1655Δ98k with a 98 kb deletion was screened out. The knockout fragment was divided into different regions to confirm the specific genes that determined the isobutanol tolerance. First, the 98 kb fragment was divided into three regions in a direction of 5′ to 3′, and these regions were individually knocked out. Plasmids pHCY108‐1, pHCY108‐2, and pHCY108‐3 were constructed for knockout of region 1, 2, and 3, respectively. Plasmids pHCY108‐1, pHCY108‐2, and pHCY108‐3 were individually co‐transformed with pHCY‐25E into *E. coli* MG1655 for knockout. The knockout method was same as the above “Knockout of *lacIZYA* in *E. coli* MG1655”. The generated strains *E. coli* MG1655Δ98k‐1, *E. coli* MG1655Δ98k‐2, and *E. coli* MG1655Δ98k‐3 were fed with 0, 6, and 8 g L^−1^ isobutanol, respectively, in order to verify the isobutanol tolerance. The strains *E. coli* MG1655Δ98k‐2‐1, *E. coli* MG1655Δ98k‐2‐2, *E. coli* MG1655Δ98k‐2‐3, and *E. coli* MG1655Δ98k‐2‐4 were obtained using the above‐mentioned method.

### Statistical Analysis

GraphPad Prism 8.0 software was used for data processing. All values and error bars represent mean and SD (*n* = 3), respectively, and differences between two groups were compared with two‐tailed *t*‐test. Statistical significance was accepted for values of *P* < 0.1. Dose‐response correlation between AA concentration and GFP/OD_600_ value was simulated by “Dose‐response‐Inhibition” in GraphPad Prism 8.0.

## Conflict of Interest

The authors declare no conflict of interest.

## Author Contributions

Z.C. and S.Y. contributed equally to this work. Y.‐X.H. and Z.C. generated the idea. Y.‐X.H., Z.C., and S.Y. designed the project. S.Y., Z.C., J.L., L.G., T.W., P.D., D.Y., and C.H. carried out the experiments. Z.C., S.Y., and Y.‐X.H. analyzed the data. Z.C., S.Y., and Y.‐X.H. wrote the manuscript.

## Supporting information

Supporting Information

## Data Availability

The data that support the findings of this study are available from the corresponding author upon reasonable request.
