# Supplementary material for: Concentration Recognition‐Based Auto‐Dynamic Regulation System (CRUISE) Enabling Efficient Production of Higher Alcohols
Source: Adv Sci (Weinh). 2024 Apr 16;11(23):2310215. doi: 10.1002/advs.202310215 (PMC11187965; doi:10.1002/advs.202310215)
Supplement: Supplementary file 1 — Supporting Information [file ADVS-11-2310215-s001.pdf]

ADVANCED SCIENCE
Open Access

Supporting Information

for *Adv. Sci.*, DOI 10.1002/advs.202310215

Concentration Recognition-Based Auto-Dynamic Regulation System (CRUISE) Enabling Efficient Production of Higher Alcohols

*Zhenya Chen, Shengzhu Yu, Jing Liu, Liwei Guo, Tong Wu, Peifeng Duan, Dongli Yan, Chaoyong Huang and Yi-Xin Huo**

<u>C</u>oncentration <u>r</u>ecognition-based a<u>u</u>to-dynamic regulat<u>i</u>on <u>s</u>yst<u>e</u>m (CRUISE) enabling efficient production of higher alcohols

Supplementary

Contents:

Methods

Table s1-5

Figure s1-35

Methods

Establishment and high-throughput screening of *ivbL* mutagenesis library

Error-prone PCR was performed on *ivbL* and terminator loop region, resulting in an *ivbL* mutagenesis library. The pink protein gene *pink* was assembled downstream of *ivbL-lacI-*P_L*lacO*$_1$ fragment to generate plasmid pS-iL-P, which was used for visual screening of highly responsive *ivbL* mutants. For error-prone PCR, 10× unbalanced dNTPs mixture containing 2 mM dATP, 2 mM dGTP, 8 mM dCTP and 8 mM dTTP was prepared. 100 µL reaction mixture contained 1 µL 0.4 ng/L original plasmid pS-iL-P, 10 µL 10× unbalanced dNTPs mixture, 1.5 µL 25 mM $MnCl_2$, 2 µL 200 mM $MgCl_2$, 2 µL 10 µM forward primer, 2 µL 10 µM reverse primer, 2 µL 5 U/µL HieffTM Taq DNA Polymerase, 10 µL 10×M5 Taq PCR Buffer (Mg^{2+} free) and 70 µL ddH_2O.The amplification program was of 94 °C initial denaturation for 3 minutes and then 30 cycles of 94 °C denaturation for 1 minute, 56 °C annealing for 1 minute and 72 °C extension for 1 min. PCR product was purified via gel extraction kit and digested by *Dpn* I at 37 °C for 1 hour. Then, the generated product was cloned into pS-iL-P by Gibson Assembly, and 10 µL of the ligation product was transformed into 50 µL *E. coli* MG1655Δ*lacIZYA* competent cells. The transformants were incubated at 37 °C for 12 hours. The colonies on plate with deeper pink were selected as the candidates that may have higher response to L-Val or L-Leu when compared with the wild-type *ivbL*. The mutants were sequenced, amplified and assembled upstream of *lacI-*P_L*lacO*$_1$*-gfp* via Gibson Assembly. The generated plasmid was transformed into *E. coli* MG1655Δ*lacIZYA* and then incubated in 0.2×LB and M9NY medium. The highly responsive *ivbL* mutants was confirmed by detection of GFP/OD_{600} values through addition of L-Val or L-Leu.

Fluorescence kinetic detection of *ivbL* mutants

To characterize the *ivbL* transcription attenuation system and identify the trend of the response intensity of *ivbL* system to L-Val and L-Leu, the corresponding *E. coli* transformants were cultured in 3 mL LB medium with appropriate antibiotics at 37 °C at 220 rpm for 8 hours. Then 4 µL cultures were transferred into a black 96-well plate with clear bottom (BRAND plates) containing 200 µL of M9NY medium with various concentrations of amino acids (0-0.5 mM) and appropriate antibiotics. The plate was incubated in the plate reader (BioTek Cytation 3) with continuous shaking at 30 °C

for 20 hours. The OD_{600} values and fluorescent intensities were quantified every 30 minutes. The excitation and emission wavelengths of green fluorescent were set at 470 and 510 nm, respectively.

Fluorescence assays of *ivbL* mutants

Single colonies were cultivated in 3 mL LB medium with appropriate antibiotics for 8 hours at 37 °C at 220 rpm. Each sample of culture was washed with 0.2×LB medium to remove the residual LB and then inoculated into 3 mL fresh 0.2×LB medium with a final OD_{600} value of 0.1. Meanwhile, each sample of culture was washed with M9NY medium to remove the residual LB and then inoculated into 3 mL fresh M9NY medium with a final OD_{600} value of 0.1. The generated culture was then cultivated at 30 °C at 220 rpm for 20 hours. Without addition of L-Val or L-Leu, the GFP/OD_{600} values of the highly responsive *ivbL* mutants was detected by Microplate reader.

Plasmid construction

To express AlsS, IlvCD, CipA-AlsS and CipA-IlvCD, genes *alsS*, *ilvCD*, *cipA-alsS* and *cipA-ilvCD* were individually amplified from the plasmid pS-B-CA-CICD. Fragments *alsS*, *ilvCD*, *cipA-alsS* and *cipA-ilvCD* were individually assembled into plasmid pET-28a, to generate plasmids pET28a-A, pET28a-ICD, pET28a-CA and pET28a-CICD.

Protein expression

Single colonies of the strain BL21(DE3) harboring pET28a-A, pET28a-CD, pET28a-CA or pET28a-CICD were cultivated in 4 mL LB medium with appropriate antibiotics at 37 °C at 220 rpm for 8 hours. Then 2 mL cultures were inoculated into 200 mL TB and cultivated at 37 °C at 220 rpm. When the OD_{600} value reached 0.8, IPTG was added into the culture with a final concentration of 0.5 mM to induce enzyme expression at 30 °C at 220 rpm for 6 hours. After expression, the cells were collected and resuspended in Tris-HCl buffer containing 20 mM imidazole, followed by ultrasonic treatment to obtain the crude enzyme extract.

AlsS assay

Enzyme AlsS could convert pyruvate into acetolactate, and the acetolactate could be hydrolyzed by acid to form acetoin. Based on this, the reaction mixture containing 100 μM TPP (thiamine pyrophosphate), 20 mM sodium pyruvate, 100 mM MOPS and 1 mM $MgCl_2$ was prepared. The reaction was initiated by the addition of 20 μL AlsS crude extract to 180 μL reaction mixture, and then proceed at 37 ℃ for 30 minutes. The reaction was terminated by 20 μL of 50% H_2SO_4. The generated mixture was incubated for an additional 5 minutes at 37 °C to allow the acid hydrolysis of acetolactate to acetoin. 40 μL sample or acetoin standard was transferred into 160 μL 0.45 M NaOH. 100 μL of the generated mixture was transferred into 100 μL of 1:1 reagent (0.5% creatine and 5% 1-naphthol in 2.5 M NaOH) to maintain at 37 ℃ for 10 minutes. After that, the absorbance value of the solution at 535 nm was measured to calculate the acetoin concentration.

E. coli MG1655	Significance	Glucose consumption (g/L)	Isobutanol (g/L)	Percentage of theoretical yield (%)	Conditions
Δ*lacIZYA*	None	10	0	0	M9NY
Δ*lacIZYA*	None of CRUISE	10	0.66	16.1	M9NY
Δ*lacIZYA*	CRUISE	10	1.32	32.2	M9NY
Δ*lacIZYA*	Self-assembly-aided CRUISE	10	1.78	43.4	M9NY
Δ*lacIZYA*	CRUISE	40	9.25	56.4	M9Y
Δ98k-2-4	CRUISE	40	14.9	90.9	M9Y
Δ*lacIZYA*	CRUISE	135	23.5	42.5	Fed-batch
Δ98k-2-4	Self-assembly-aided CRUISE	233	40.4	42.3	Fed-batch

Table s1 Isobutanol production

Δ98k-2-4: HA-tolerant strain

Table s2 Sequences of *ivbL* mutants

Name	Sequence
ivbL-7	atgactacttccatgctcaacgcaaaactactaccaactgcgccatccgccgcagtggtcgtcatgcgtgtggtg<br>gtggtcgtcggcaatgcgccgtagggactggtacaacacacgattccaaaaccccgccggcgcaaaccggg<br>cggggtttttcgtttaagcacctcccggaaagtcggtcctgaagaaaaggactggagc
ivbL-8	atgactactaccatgctcaacgcaaaactactaccaactgcgccatccgccgcagtggtcgtcgtgcgtgtggt<br>ggtggtcgtcggcaatgcgccgtagggactggaacaacacacgattccaaagccccgccggcgcaaaccg<br>ggcggggtttttcgtttaagcacctcccggaatgtcggtccagaagaaaaggactggagc
ivbL-13	atgactacttccatgctcaacgcaaaactactaccaactgcgccatccgccgcagtggtcgtcgtgcgtgtggt<br>ggtggtcgtcggcaatgcgccgtagggactgggacaacacacgattccaaaaccccgccggcgcaaaccg<br>ggcggggtttttcgtttaagcacctcccggaaagtcggcccagaagaaaaagactggagc
ivbL-19	aagactacttccatgctcaacggaaaactactaccaactgcgccatccgccgcagtggtcgtcgtgcgtgtggt<br>ggtggtcgtcggcaatgcgccgtagggactggaacaacacacgattccaaaaccccgccggcgcaaaccgg<br>gcggggtttttcgtttaagcacctcccggaaagtcggtccagaagaaaaggactggagc
ivbL-26	atgactacttccatgctcaacgcaaaactactaccaactgcgccatccgccgcagtggtcgtcgtgcgtgtggt<br>ggtggtcgtcggcaatgcgccgtagggactggaactacacacgattccaaaaccccgccggcgcaaaccgg<br>gcggggtttttcgtttaagcgcctcccggaaagtcggtccagaagaataggactggagc
ivbL-ser (*leu*)	atgactacttccatgtccaacgcaaaatcctccccaactgcgccatccgccgcagtggtcgtcgtgcgtgtggt<br>ggtggtcgtcggcaatgcgccgtagggactggaacaacacacgattccaaaaccccgccggcgcaaaccgg<br>gcggggtttttcgtttaagcacctcccggaaagtcggcccagaagaaaaggactggagc
ivbL-thr	atgactacttccatgacgaacgcaaaaacgacgccaactgcgccatccgccgcaacgacgacgacgcgtac<br>gacgacgacgacgggcaatgcgccgtagggactggaacaacacacgattccaaaaccccgccggcgcaaa<br>ccgggcggggtttttcgtttaagcacctcccggaaagtcggcccagaagaaaaggactggagc
ivbL-pro	atgactacttccatgccaaacgcaaaaccaccaccaactgcgccatccgccgcaccaccaccaccacgtcca<br>ccaccaccaccaggcaatgcgccgtagggactggaacaacacacgattccaaaaccccgccggcgcaaac<br>cgggcggggtttttcgtttaagcacctcccggaaagtcggcccagaagaaaaggactggagc
ivbL-ile	atgactacttccatgatcaacgcaaaaatcatcccaactgcgccatccgccgcaatcatcatcatccgtatcatca<br>tcatcatcggcaatgcgccgtagggactggaacaacacacgattccaaaaccccgccggcgcaaaccgggc<br>ggggtttttcgtttaagcacctcccggaaagtcggcccagaagaaaaggactggagc
ivbL-tyr	atgactacttccatgtacaacgcaaaatactacccaactgcgccatccgccgcatactactactaccgttactact<br>actactacggcaatgcgccgtagggactggaacaacacacgattccaaaaccccgccggcgcaaaccgggc<br>ggggtttttcgtttaagcacctcccggaaagtcggcccagaagaaaaggactggagc
ivbL-asp	atgactacttccatggacaacgcaaaagacgacccaactgcgccatccgccgcagacgacgacgaccgtga<br>cgacgacgacgacggcaatgcgccgtagggactggaacaacacacgattccaaaaccccgccggcgcaaa<br>ccgggcggggtttttcgtttaagcacctcccggaaagtcggcccagaagaaaaggactggagc
ivbL-asn	atgactacttccatgaataacgcaaaaaataatccaactgcgccatccgccgcaaataataataatcgtaataata<br>ataataatggcaatgcgccgtagggactggaacaacacacgattccaaaaccccgccggcgcaaaccgggc<br>ggggtttttcgtttaagcacctcccggaaagtcggcccagaagaaaaggactggagc

ivbL-cys	atgactacttccatgtgtaacgcaaaatgttgtccaactgcgccatccgccgcatgttgttgttgtcgttgttgttgtt<br>gttgtggcaatgcgccgtagggactggaacaacacacgattccaaaccccgccggcgcaaaccgggcgg<br>ggtttttcgtttaagcacctcccggaaagtcggcccagaagaaaaggactggagc
ivbL-arg	atgactacttccatgcggaacgcaaaacggcggccaactgcgccatccgccgcacggcggcggcggcgtc<br>ggcggcggcggcggggcaatgcgccgtagggactggaacaacacacgattccaaaccccgccggcgca<br>aaccgggcggggtttttcgtttaagcacctcccggaaagtcggcccagaagaaaaggactggagc
ivbL-ser	atgactacttccatgtcaaacgcaaaatcatcaccaactgcgccatccgccgcatcatcatcatcacgttcatcat<br>catcatcaggcaatgcgccgtagggactggaacaacacacgattccaaaccccgccggcgcaaaccgggc<br>ggggtttttcgtttaagcacctcccggaaagtcggcccagaagaaaaggactggagc
ivbl-gln	atgactacttccatgcaaaacgcaaaacaacaaccaactgcgccatccgccgcacaacaacaacaacgtcaa<br>caacaacaacaaggcaatgcgccgtagggactggaacaacacacgattccaaaccccgccggcgcaaac<br>cgggcggggtttttcgtttaagcacctcccggaaagtcggcccagaagaaaaggactggagc
ivbl-his	atgactacttccatgcacaacgcaaaacaccacccaactgcgccatccgccgcacaccaccaccaccgtcac<br>caccaccaccacggcaatgcgccgtagggactggaacaacacacgattccaaaccccgccggcgcaaac<br>cgggcggggtttttcgtttaagcacctcccggaaagtcggcccagaagaaaaggactggagc
ivbl-ala	atgactacttccatggcaaacgcaaaagcagcaccaactgcgccatccgccgcagcagcagcagcacgtgc<br>agcagcagcagcaggcaatgcgccgtagggactggaacaacacacgattccaaaccccgccggcgcaaa<br>ccgggcggggtttttcgtttaagcacctcccggaaagtcggcccagaagaaaaggactggagc
ivbl-glu	atgactacttccatggagaacgcaaaagaggagccaactgcgccatccgccgcagaggaggaggag<br>cgtgaggaggaggaggagggcaatgcgccgtagggactggaacaacacacgattccaaaccccgccgg<br>cgcaaaccgggcggggtttttcgtttaagcacctcccggaaagtcggcccagaagaaaaggactggagc
ivbl-gly	atgactacttccatgggaaacgcaaaaggaggaccaactgcgccatccgccgcaggaggaggaggacgtg<br>gaggaggaggaggaggcaatgcgccgtagggactggaacaacacacgattccaaaccccgccggcgca<br>aaccgggcggggtttttcgtttaagcacctcccggaaagtcggcccagaagaaaaggactggagc
ivbl-lys	atgactacttccatgaagaacgcaaaaaagaagccaactgcgccatccgccgcaaagaagaagaagcgtaa<br>gaagaagaagaagggcaatgcgccgtagggactggaacaacacacgattccaaaccccgccggcgcaaa<br>ccgggcggggtttttcgtttaagcacctcccggaaagtcggcccagaagaaaaggactggagc
ivbl-phe	atgactacttccatgttcaacgcaaaattcttcccaactgcgccatccgccgcattcttcttcttccgtttcttcttcttc<br>ttcggcaatgcgccgtagggactggaacaacacacgattccaaaccccgccggcgcaaaccgggcggggt<br>ttttcgtttaagcacctcccggaaagtcggcccagaagaaaaggactggagc
ivbl-met	atgactacttccatgatgaacgcaaaaatgatgccaactgcgccatccgccgcaatgatgatgatgcgtatgatg<br>atgatgatgggcaatgcgccgtagggactggaacaacacacgattccaaaccccgccggcgcaaaccggg<br>cggggtttttcgtttaagcacctcccggaaagtcggcccagaagaaaaggactggagc
ivbl-trp	atgactacttccatgtggaacgcaaaatggtggccaactgcgccatccgccgcatggtggtggtggcgttggtg<br>gtggtggtggggcaatgcgccgtagggactggaacaacacacgattccaaaccccgccggcgcaaaccg<br>ggcggggtttttcgtttaagcacctcccggaaagtcggcccagaagaaaaggactggagc

Note: the bases highlighted in red are mutated bases.

Table s3 Plasmids and strains used in this study

Plasmids and strains	Description	Source
Plasmids		
pS-AII	*$P_L lacO_1$-alsS-ilvC-ilvD; p15A; kan^r*	Storage
pS-LKY	*$P_L lacO_1$-leuDH-kivD-yqhD; colE1; amp^r*	Storage
pYH1	*P_{bmoR}-bmoR; P_{bmo}-gfp; colE1; amp^r*	[1]
pHCY-25E	P_{BAD}-Cas9-PT5-Redγβα; *p15A; kan^r*	[2]
pS-iG	*P_{ivbL}-ivbL-gfp; p15A; cm^r*	This study
pS-iL-G	*P_{ivbL}-ivbL-lacI; $P_L lacO_1$-gfp; p15A; cm^r*	This study
pS-iL-LKY	*P_{ivbL}-ivbL-lacI; $P_L lacO_1$-leuDH-kivD-yqhD; p15A; cm^r*	This study
pS-B-AII	*P_{bmoR}-bmoR; P_{bmo}-alsS-ilvC-ilvD; colE1; amp^r*	This study
pS-AII-R	*$P_L lacO_1$-alsS-ilvC-ilvD-rfp; p15A; kan^r*	This study
pS-LKY-G	*$P_L lacO_1$-leuDH-kivD-yqhD-gfp; colE1; amp^r*	This study
pS-B-AII-R	*P_{bmoR}-bmoR; P_{bmo}-alsS-ilvC-ilvD-rfp; colE1; amp^r*	This study
pS-iL-LKY-G	*P_{ivbL}-ivbL-lacI; $P_L lacO_1$-leuDH-kivD-yqhD-gfp; p15A; cm^r*	This study
pS-B-AII-L	*P_{bmoR}-bmoR; P_{bmo}-alsS-ilvC-ilvD-leuABCD; colE1; amp^r*	This study
pS-AII-L	*$P_L lacO_1$-alsS-ilvC-ilvD-leuABCD; p15A; kan^r*	This study
pS-B-CA-CIC-ID	*P_{bmoR}-bmoR; P_{bmo}-cipA-alsS-cipA-ilvC-ilvD; colE1; amp^r*	This study
pS-B-CA-CIC-CID	*P_{bmoR}-bmoR; P_{bmo}-cipA-alsS-cipA-ilvC-cipA-ilvD; colE1; amp^r*	This study
pS-B-CA-CICD	*P_{bmoR}-bmoR; P_{bmo}-cipA-alsS-cipA-ilvCD; colE1; amp^r*	This study
pS-B-CA-CIC-ID-CLA-LBCD	*P_{bmoR}-bmoR; P_{bmo}-cipA-alsS-cipA-ilvC-ilvD-cipA-leuABCD; colE1; amp^r*	This study
pET28a-A	*P_{T7}-alsS; P_{lacI}-lacI; colE1; kan^r*	This study
pET28a-ICD	*P_{T7}-ilvCD; P_{lacI}-lacI; colE1; kan^r*	This study

pET28a-CA	P_{T7}-*cipA-alsS*; P_{lacI}-*lacI*; *colE1*; kan^{r}	This study
pET28a-CICD	P_{T7}-*cipA-ilvCD*; P_{lacI}-*lacI*; *colE1*; kan^{r}	This study
pHCY26D-*lacIZYA*	P_{BAD}-*sgRNAΔlacIZYA-DonorΔlacIZYA*; *pSC101*; amp^{r}	This study
pHCY108-1	P_{BAD}-*sgRNAΔ98k-1-DonorΔ98k-1*; *pSC101*; amp^{r}	This study
pHCY108-2	P_{BAD}-*sgRNAΔ98k-2-DonorΔ98k-2*; *pSC101*; amp^{r}	This study
pHCY108-3	P_{BAD}-*sgRNAΔ98k-3-DonorΔ98k-3*; *pSC101*; amp^{r}	This study
pHCY108-2-1	P_{BAD}-*sgRNAΔ98k-2-1-DonorΔ98k-2-1*; *pSC101*; amp^{r}	This study
pHCY108-2-2	P_{BAD}-*sgRNAΔ98k-2-2-DonorΔ98k-2-2*; *pSC101*; amp^{r}	This study
pHCY108-2-3	P_{BAD}-*sgRNAΔ98k-2-3-DonorΔ98k-2-3*; *pSC101*; amp^{r}	This study
pHCY108-2-4	P_{BAD}-*sgRNAΔ98k-2-4-DonorΔ98k-24*; *pSC101*; amp^{r}	This study
pYH1-V311A	P_{bmoR}-$bmoR^{V311A}$; P_{bmo}-*gfp*; *colE1*; amp^{r}	[3]
pS-L-G	P_{lacI} -*lacI*; $P_{L}lacO_{1}$-*gfp*; *p15A*; cm^{r}	This study
pS-B^{T12N}-AII	P_{bmoR}- $bmoR^{T12N}$; P_{bmo}-*alsS-ilvC-ilvD*; *colE1*; amp^{r}	This study
pS-iL-MAKY	P_{ivbL}-*ivbL-lacI*; $P_{L}lacO_{1}$-*mfl-aad-kivD-yqhD*; *p15A*; cm^{r}	This study
pS-B^{V311A}-AII	P_{bmo}- $bmoR^{V311A}$-*alsS-ilvC-ilvD*; *colE1*; amp^{r}	This study
pS-iL-P	P_{ivbL} -*ivbL-lacI*; $P_{L}lacO_{1}$-*pink*; *p15A*; cm^{r}	This study
pS-i7L-G	P_{ivbL}-*ivbL-7-lacI*; $P_{L}lacO_{1}$-*gfp*; *p15A*; cm^{r}	This study
pS-i8L-G	P_{ivbL}-*ivbL-8-lacI*; $P_{L}lacO_{1}$-*gfp*; *p15A*; cm^{r}	This study
pS-i13L-G	P_{ivbL}-*ivbL-13-lacI*; $P_{L}lacO_{1}$-*gfp*; *p15A*; cm^{r}	This study
pS-i19L-G	P_{ivbL}-*ivbL-19-lacI*; $P_{L}lacO_{1}$-*gfp*; *p15A*; cm^{r}	This study
pS-i26L-G	P_{ivbL}-*ivbL-26-lacI*; $P_{L}lacO_{1}$-*gfp*; *p15A*; cm^{r}	This study
pS-i13L-LKY	P_{ivbL}-*ivbL-13-lacI*; $P_{L}lacO_{1}$-*leuDH-kivD-yqhD*; *p15A*; cm^{r}	This study
pS-i19L-LKY	P_{ivbL}-*ivbL-19-lacI*; $P_{L}lacO_{1}$-*leuDH-kivD-yqhD*; *p15A*; cm^{r}	This study
pS-iSL-G	P_{ivbL}-*ivbL-ser(leu)-lacI*; $P_{L}lacO_{1}$-*gfp*; *p15A*; cm^{r}	

pS-iSL-LKY	*P_{ivbL}-ivbL-ser(leu)-lacI; $P_L$lacO$_1$-leuDH-kivD-yqhD; p15A; cm^r*	This study
pS-i-T-L-G	*P_{ivbL}-ivbL-thr-lacI; $P_L$lacO$_1$-gfp; p15A; cm^r*	This study
pS-i-P-L-G	*P_{ivbL}-ivbL-pro-lacI; $P_L$lacO$_1$-gfp; p15A; cm^r*	This study
pS-i-I-L-G	*P_{ivbL}-ivbL-ile-lacI; $P_L$lacO$_1$-gfp; p15A; cm^r*	This study
pS-i-Y-L-G	*P_{ivbL}-ivbL-tyr-lacI; $P_L$lacO$_1$-gfp; p15A; cm^r*	This study
pS-i-D-L-G	*P_{ivbL}-ivbL-asp-lacI; $P_L$lacO$_1$-gfp; p15A; cm^r*	This study
pS-i-N-L-G	*P_{ivbL}-ivbL-asn-lacI; $P_L$lacO$_1$-gfp; p15A; cm^r*	This study
pS-i-C-L-G	*P_{ivbL}-ivbL-cys-lacI; $P_L$lacO$_1$-gfp; p15A; cm^r*	This study
pS-i-R-L-G	*P_{ivbL}-ivbL-arg-lacI; $P_L$lacO$_1$-gfp; p15A; cm^r*	This study
pS-i-S-L-G	*P_{ivbL}-ivbL-ser-lacI; $P_L$lacO$_1$-gfp; p15A; cm^r*	This study
pS-i-Q-L-G	*P_{ivbL}-ivbL-gln-lacI; $P_L$lacO$_1$-gfp; p15A; cm^r*	This study
pS-i-H-L-G	*P_{ivbL}-ivbL-his-lacI; $P_L$lacO$_1$-gfp; p15A; cm^r*	This study
pS-i-A-L-G	*P_{ivbL}-ivbL-ala-lacI; $P_L$lacO$_1$-gfp; p15A; cm^r*	This study
pS-i-E-L-G	*P_{ivbL}-ivbL-glu-lacI; $P_L$lacO$_1$-gfp; p15A; cm^r*	This study
pS-i-G-L-G	*P_{ivbL}-ivbL-gly-lacI; $P_L$lacO$_1$-gfp; p15A; cm^r*	This study
pS-i-K-L-G	*P_{ivbL}-ivbL-lys-lacI; $P_L$lacO$_1$-gfp; p15A; cm^r*	This study
pS-i-F-L-G	*P_{ivbL}-ivbL-phe-lacI; $P_L$lacO$_1$-gfp; p15A; cm^r*	This study
pS-i-M-L-G	*P_{ivbL}-ivbL-met-lacI; $P_L$lacO$_1$-gfp; p15A; cm^r*	This study
pS-i-W-L-G	*P_{ivbL}-ivbL-trp-lacI; $P_L$lacO$_1$-gfp; p15A; cm^r*	This study
E. coli strains		
DH5α	F$^-$ λ$^-$ *endA1 glnV44 thi-1 recA1 relA1 gyrA96 deoR nupG purB20 φ80dlacZΔM1Δ(lacZYA-argF)U169, hsdR17*($r_k^- m_k^+$)	Storage
MG1655	F$^-$ λ$^-$ilvG$^-$ *rfb-50 rph-1*	Storage
BL21(DE3)	*F$^-$ ompT hsdS (rB$^-$mB$^-$) gal dcm (DE3)*	Storage
MG1655Δ98k	MG1655 derivative, deletion of 98kb fragment location (1,549,491~1,647,484) on the MG1655 genome	Storage

MG1655Δ*lacIZYA*	MG1655 derivative, deletion of *lacIZYA* on the MG1655 genome	This study
MG1655Δ98k-1	MG1655 derivative, deletion of fragment location (1,549,491~1,598,691) on the MG1655 genome	This study
MG1655Δ98k-2	MG1655 derivative, deletion of fragment location (1,598,670~1,622,839) on the MG1655 genome	This study
MG1655Δ98k-3	MG1655 derivative, deletion of fragment location (1,622,799~1,647,484) on the MG1655 genome	This study
MG1655Δ98k-2-1	MG1655 derivative, deletion of fragment location (1,598,670~1,604,669) on the MG1655 genome	This study
MG1655Δ98k-2-2	MG1655 derivative, deletion of fragment location (1,604,670~1,610,669) on the MG1655 genome	This study
MG1655Δ98k-2-3	MG1655 derivative, deletion of fragment location (1,610,670~1,616,669) on the MG1655 genome	This study
MG1655Δ98k-2-4	MG1655 derivative, deletion of fragment location (1,616,670~1,622,839) on the MG1655 genome	This study

References

1. Yu, H. et al. Establishment of BmoR-based biosensor to screen isobutanol overproducer. *Microb Cell Fact* 18, 30 (2019).
2. Huang, C. et al. CRISPR-Cas9-assisted native end-joining editing offers a simple strategy for efficient genetic engineering in *Escherichia coli*. *Appl Microbiol Biotechnol* 103, 8497-8509 (2019).
3. Wu, T., Chen, Z., Guo, S., Zhang, C. & Huo, Y.X. Engineering Transcription Factor BmoR Mutants for Constructing Multifunctional Alcohol Biosensors. *ACS Synth Biol* 11, 1251-1260 (2022).

Table s4 Sequences of overexpression genes

Name	Sequence
ivbL	atgactacttccatgctcaacgcaaaactactaccaactgcgccatccgccgcagtggtcgtcgtgcgtgtggtggtggtcgtcggcaatgcgccgtagggactggaacaacacacgattccaaaaccccgccggcgcaaaccgggcggggtttttcgtttaagcacctcccggaaagtcggcccagaagaaaaggactggagc
lacI	atgaaaccagtaacgttatacgatgtcgcagagtatgccggtgtctcttatcagaccgtttcccgcgtggtgaaccaggccagccacgtttctgcgaaaacgcgggaaaaagtggaagcggcgatggcggagctgaattacattcccaaccgcgtggcacaacaactggcgggcaaacagtcgttgctgattggcgttgccacctccagtctggccctgcacgcgccgtcgcaaattgtcgcggcgattaaatctcgcgccgatcaactgggtgccagcgtggtggtgtcgatggtagaacgaagcggcgtcgaagcctgtaaagcggcggtgcacaatcttctcgcgcaacgcgtcagtgggctgatcattaactatccgctggatgaccaggatgccattgctgtggaagctgcctgcactaatgttccggcgttatttcttgatgtctctgaccagacacccatcaacagtattattttctcccatgaagacggtacgcgactgggcgtggagcatctggtcgcattgggtcaccagcaaatcgcgctgttagcgggcccattaagttctgtctcggcgcgtctgcgtctggctggctggcataaatatctcactcgcaatcaaattcagccgatagcggaacgggaaggcgactggagtgccatgtccggttttcaacaaaccatgcaaatgctgaatgagggcatcgttcccactgcgatgctggttgccaacgatcagatggcgctgggcgcaatgcgcgccattaccgagtccgggctgcgcgttggtgcggatatctcggtagtgggatacgacgataccgaagacagctcatgttatatcccgccgttaaccaccatcaaacaggattttcgcctgctggggcaaaccagcgtggaccgcttgctgcaactctctcagggccaggcggtgaagggcaatcagctgttgcccgtctcactggtgaaaagaaaaaccaccctggcgcccaatacgcaaaccgcctctccccgcgcgttggccgattcattaatgcagctggcacgacaggtttcccgactggaaagcgggcagtga
alsS	atgttgacaaaagcaacaaaagaacaaaaatcccttgtgaaaaacagaggggcggagcttgttgttgattgcttagtggagcaaggtgtcacacatgtatttggcattccaggtgcaaaaattgatgcggtatttgacgctttacaagataaaggacctgaaattatcgttgcccggcacgaacaaaacgcagcattcatggcccaagcagtcggccgtttaactggaaaaccgggagtcgtgttagtcacatcaggaccgggtgcctctaacttggcaacaggcctgctgacagcgaacactgaaggagaccctgtcgttgcgcttgctggaaacgtgatccgtgcagatcgtttaaaacggacacatcaatctttggataatgcggcgctattccagccgattacaaaatacagtgtagaagttcaagatgtaaaaaatataccggaagctgttacaaatgcatttaggatagcgtcagcagggcaggctggggccgcttttgtgagctttccgcaagatgttgtgaatgaagtcacaaatacgaaaaacgtgcgtgctgttgcagcgccaaaactcggtcctgcagcagatgatgcaatcagtgcggccatagcaaaaatccaaacagcaaaacttcctgtcgttttggtcggcatgaaaggcggaagaccggaagcaattaaagcggttcgcaagcttttgaaaaaggttcagcttccatttgttgaaacatatcaagctgccggtaccctttctagagatttagaggatcaatattttggccgtatcggtttgttccgcaaccagcctggcgatttactgctagagcaggcagatgttgttctgacgatcggctatgacccgattgaatatgat

ccgaaattctggaatatcaatggagaccggacaattatccatttagacgagattatcgctgacattgatcatgcttaccag

cctgatcttgaattgatcggtgacattccgtccacgatcaatcatatcgaacacgatgctgtgaaagtggaatttgcagag

cgtgagcagaaaatcctttctgatttaaaacaatatatgcatgaaggtgagcaggtgcctgcagattggaaatcagacag

agcgcaccctcttgaaatcgttaaagagttgcgtaatgcagtcgatgatcatgttacagtaacttgcgatatcggttcgca

cgccatttggatgtcacgttatttccgcagctacgagccgttaacattaatgatcagtaacggtatgcaaacactcggcgtt

gcgcttccttgggcaatcggcgcttcattggtgaaaccgggagaaaaagtggtttctgtctctggtgacggcggtttctta

ttctcagcaatggaattagagacagcagttcgactaaaagcaccaattgtacacattgtatggaacgacagcacatatga

catggttgcattccagcaattgaaaaaatataaccgtacatctgcggtcgatttcggaaatatcgatatcgtgaaatatgcg

gaaagcttcggagcaactggcttgcgcgtagaatcaccagaccagctggcagatgttctgcgtcaaggcatgaacgct

gaaggtcctgtcatcatcgatgtcccggttgactacagtgataacattaatttagcaagtgacaagcttccgaaagaattc

ggggaactcatgaaaacgaaagctctctag

atggctaactacttcaatacactgaatctgcgccagcagctggcacagctgggcaaatgtcgctttatgggccgcgatg

aattcgccgatggcgcgagctaccttcagggtaaaaaagtagtcatcgtcggctgtggcgcacagggtctgaaccagg

gcctgaacatgcgtgattctggtctcgatatctcctacgctctgcgtaaagaagcgattgccgagaagcgcgcgtcctg

gcgtaaagcgaccgaaaatggttttaaagtgggtacttacgaagaactgatcccacaggcggatctggtgattaacctg

acgccggacaagcagcactctgatgtagtgcgcaccgtacagccactgatgaaagacggcgcggcgctgggctact

cgcacggtttcaacatcgtcgaagtgggcgagcagatccgtaaagatatcaccgtagtgatggttgcgccgaaatgcc

caggcaccgaagtgcgtgaagagtacaaacgtgggttcggcgtaccgacgctgattgccgttcacccggaaaacgat

ccgaaaggcgaaggcatggcgattgccaaagcctgggcggctgcaaccggtggtcaccgtgcgggtgtgctggaat

cgtccttcgttgcggaagtgaaatctgacctgatgggcgagcaaaccatcctgtgcggtatgttgcaggctggctctctg

ilvC

ctgtgcttcgacaagctggtggaagaaggtaccgatccagcatacgcagaaaaactgattcagttcggttgggaaacc

atcaccgaagcactgaaacagggcggcatcaccctgatgatggaccgtctctctaacccggcgaaactgcgtgcttat

gcgctttctgaacagctgaaagagatcatggcacccctgttccagaaacatatggacgacatcatctccggcgaattctc

ttccggtatgatggcggactgggccaacgatgataagaaactgctgacctggcgtgaagagaccggcaaaaccgcgt

ttgaaaccgcgccgcagtatgaaggcaaaatcggcgagcaggagtacttcgataaaggcgtactgatgattgcgatgg

tgaaagcgggcgttgaactggcgttcgaaaccatggtcgattccggcatcattgaagagtctgcatattatgaatcactg

cacgagctgccgctgattgccaacaccatcgcccgtaagcgtctgtacgaaatgaacgtggttatctctgataccgctga

gtacggtaactatctgttctcttacgcttgtgtgccgttgctgaaaccgtttatggcagagctgcaaccgggcgacctggg

taaagctattccggaaggcgcggtagataacgggcaactgcgtgatgtgaacgaagcgattcgcagccatgcgattga

gcaggtaggtaagaaactgcgcggctatatgacagatatgaaacgtattgctgttgcgggttaa

atgcctaagtaccgttccgccaccaccactcatggtcgtaatatggcgggtgctcgtgcgctgtggcgcgccaccgga

atgaccgacgccgatttcggtaagccgattatcgcggttgtgaactcgttcacccaatttgtaccgggtcacgtccatctg

cgcgatctcggtaaactggtcgccgaacaaattgaagcggctggcggcgttgccaaagagttcaacaccattgcggtg

gatgatgggattgccatgggccacgggggatgctttattcactgccatctcgcgaactgatcgctgattccgttgagtat

atggtcaacgcccactgcgccgacgccatggtctgcatctctaactgcgacaaaatcaccccggggatgctgatggctt

ccctgcgcctgaatattccggtgatctttgtttccggcggcccgatggaggccgggaaaaccaaactttccgatcagatc

atcaagctcgatctggttgatgcgatgatccagggcgcagacccgaaagtatctgactcccagagcgatcaggttgaa

cgttccgcgtgtccgacctgcggttcctgctccgggatgtttaccgctaactcaatgaactgcctgaccgaagcgctgg

gcctgtcgcagccgggcaacggctcgctgctggcaacccacgccgaccgtaagcagctgttccttaatgctggtaaac

gcattgttgaattgaccaaacgttattacgagcaaaacgacgaaagtgcactgccgcgtaatatcgccagtaaggcggc

gtttgaaaacgccatgacgctggatatcgcgatgggtggatcgactaacaccgtacttcacctgctggcggcggcgca

ggaagcggaaatcgacttcaccatgagtgatatcgataagctttcccgcaaggttccacagctgtgtaaagttgcgccg

ilvD

agcacccagaaataccatatggaagatgttcaccgtgctggtggtgttatcggtattctcggcgaactggatcgcgcgg

ggttactgaaccgtgatgtgaaaaacgtacttggcctgacgttgccgcaaacgctggaacaatacgacgttatgctgac

ccaggatgacgcggtaaaaaatatgttccgcgcaggtcctgcaggcattcgtaccacacaggcattctcgcaagattgc

cgttgggatacgctggacgacgatcgcgccaatggctgtatccgctcgctggaacacgcctacagcaaagacggcgg

cctggcggtgctctacggtaactttgcggaaaacggctgcatcgtgaaaacggcaggcgtcgatgacagcatcctcaa

attcaccggcccggcgaaagtgtacgaaagccaggacgatgcggtagaagcgattctcggcggtaaagttgtcgccg

gagatgtggtagtaattcgctatgaaggcccgaaaggcggtccggggatgcaggaaatgctctacccaaccagcttcc

tgaaatcaatgggtctcggcaaagcctgtgcgctgatcaccgacggtcgtttctctggtggcacctctggtctttccatcg

gccacgtctcaccggaagcggcaagcggcggcagcattggcctgattgaagatggtgacctgatcgctatcgacatc

ccgaaccgtggcattcagttacaggtaagcgatgccgaactggcggcgcgtcgtgaagcgcaggacgctcgaggtg

acaaagcctggacgccgaaaaatcgtgaacgtcaggtctcctttgccctgcgtgcttatgccagcctggcaaccagcg

ccgacaaaggcgcggtgcgcgataaatcgaaactgggggggttaa

atgaaaatcttcgattacatggaaaaatatgattatgaacaactcgtcatgtgccaagataaagagtccggcctcaaagc

leuDH

catcatttgtatccatgttaccaccctggggccggctctgggtggtatgcgcatgtggacttacgcttcggaagaagaag

ccatcgaggatgcgctgcgcctgggaagagggatgacttacaaaaatgcagctgccggcttaaacctcggcggcgg

aaaaacggtgatcatcggcgatccgagaaaagacaaaaacgaagccatgttccgtgctttgggacgctttatccaagg

attgaacggacggtacatcacggcggaagacgtgggaaccactgtggaagacatggatatcattcatgaggaaaccc

gttacgtgacaggcgtatcgcccgctttcggttccagcggcaacccttcaccggtgacggcttacggcgtataccgtgg

aatgaaagcggctgcaaaagaagctttcggcgatgactcactcgaaggcaaagtggtggctgttcaaggagtgggac

atgttgcttacgaattatgtaagcacttgcataatgaaggggccaaattgattgtgaccgacatcaacaaagaaaatgca

gaccgtgccgtgcaagaatttggtgccgaatttgttcatccggacaaaatctatgatgtggaatgcgacatttttgctcctt

gtgcgctcggggcgatcattaatgacgaaaccattgaacgcttgaaatgcaaagtggtggccggttcggcaaacaatc

agctgaaagaagagcgccatggaaaaatgcttgaagaaaaaggaatcgtctatgcgccggattatgtgatcaatgccg

gaggcgtgatcaatgtcgccgatgaattgctgggttataaccgtgagcgtgcgatgaaaaaagtggaaggcatttacga

caaaatcctgaaagtgtttgagatcgcaaaacgggacggcattccgagctatctcgcagcagaccgcatggcggaag

aacggattgaaatgatgcgcaaaacaagaagcacatttttgcaagatcaacgcaacctgatcaattttaacaacaaataa
atgtatacagtaggagattacctattagaccgattacacgagttaggaattgaagaaatttttggagtccctggagactata
acttacaattttttagatcaaattatttcccgcaaggatatgaaatgggtcggaaatgctaatgaattaaatgcttcatatatgg
ctgatggctatgctcgtactaaaaaagctgccgcatttcttacaacctttggagtaggtgaattgagtgcagttaatggatt
agcaggaagttacgccgaaaatttaccagtagtagaaatagtgggatcacctacatcaaaagttcaaaatgaaggaaaa
tttgttcatcatacgctggctgacggtgattttaaacactttatgaaaatgcacgaacctgttacagcagctcgaactttact
gacagcagaaaatgcaaccgttgaaattgaccgagtactttctgcactattaaaagaaagaaaacctgtctatatcaactt
accagttgatgttgctgctgcaaaagcagagaaaccctcactccctttgaaaaaagaaaactcaacttcaaatacaagtg
accaagagatcttgaacaaaattcaagaaagcttgaaaaatgccaaaaaaccaatcgtgattacaggacatgaaataatt
agttttggcttagaaaaaacagtctctcaatttatttcaaagacaaaactacctattacgacattaaactttggaaaaagttca
gttgatgaagctctcccttcatttttaggaatctataatggtaaactctcagagcctaatcttaaagaattcgtggaatcagc
kivD cgacttcatcctgatgcttggagttaaactcacagactcttcaacaggagccttcactcatcatttaaatgaaaataaaatg
atttcactgaatatagatgaaggaaaaatatttaacgaaagcatccaaaattttgattttgaatccctcatctcctctctcttag
acctaagcgaaatagaatacaaaggaaaatatatcgataaaaagcaagaagactttgttccatcaaatgcgcttttatcac
aagaccgcctatggcaagcagttgaaaacctaactcaaagcaatgaaacaatcgttgctgaacaagggacatcattcttt
ggcgcttcatcaattttcttaaaaccaaagagtcattttattggtcaacccttatggggatcaattggatatacattcccagca
gcattaggaagccaaattgcagataaagaaagcagacaccttttatttattggtgatggttcacttcaacttacggtgcaag
aattaggattagcaatcagagagaaaaaattaatccaatttgctttattatcaataatgatggttatacagtcgaaagagaaatt
catggaccaaatcaaagctacaatgatattccaatgtggaattactcaaaattaccagaatcatttggagcaacagaaga
acgagtagtctcgaaaatcgttagaactgaaaatgaatttgtgtctgtcatgaaagaagctcaagcagatccaaatagaat
gtactggattgagttaattttggcaaaagaagatgcaccaaaagtactgaaaaaaatgggcaaactatttgctgaacaaa
ataaatcataa
atgaacaactttaatctgcacaccccaacccgcattctgtttggtaaaggcgcaatcgctggtttacgcgaacaaattcct
cacgatgctcgcgtattgattacctacggcggcggcagcgtgaaaaaaaccggcgttctcgatcaagttctggatgccc
yqhD tgaaaggcatggacgtgctggaatttggcggtattgagccaaaccccggcttatgaaacgctgatgaacgccgtgaaact
ggttcgcgaacagaaagtgactttcctgctggcggttggcggcggttctgtactggacggcaccaaatttatcgccgca
gcggctaactatccggaaaatatcgatccgtggcacattctgcaaacgggcggtaaagagattaaaagcgccatcccg

atgggctgtgtgctgacgctgccagcaaccggttcagaatccaacgcaggcgcggtgatctcccgtaaaaccacagg
cgacaagcaggcgttccattctgcccatgttcagccggtatttgccgtgctcgatccggtttatacctacaccctgccgcc
gcgtcaggtggctaacggcgtagtggacgcctttgtacacaccgtggaacagtatgttaccaaaccggttgatgccaaa
attcaggaccgtttcgcagaaggcattttgctgacgctaatcgaagatggtccgaaagccctgaaagagccagaaaact
acgatgtgcgcgccaacgtcatgtgggcggcgactcaggcgctgaacggtttgattggcgctggcgtaccgcaggac
tgggcaacgcatatgctgggccacgaactgactgcgatgcacggtctggatcacgcgcaaacactggctatcgtcctg
cctgcactgtggaatgaaaaacgcgataccaagcgcgctaagctgctgcaatatgctgaacgcgtctggaacatcact
gaaggttccgatgatgagcgtattgacgccgcgattgccgcaacccgcaatttctttgagcaattaggcgtgccgaccc
acctctccgactacggtctggacggcagctccatcccggctttgctgaaaaaactggaagagcacggcatgacccaac
tgggcgaaaatcatgacattacgttggatgtcagccgccgtatatacgaagccgcccgctaa
atgcgtaaaggagaagaacttttcactggagttgtcccaattcttgttgaattagatggtgatgttaatgggcacaaattttct
gtcagtggagagggtgaaggtgatgcaacatacggaaaacttacccttaaatttatttgcactactggaaaactacctgtt
ccatggccaacacttgtcactactttcggttatggtgttcaatgctttgcgagatacccagatcatatgaaacagcatgactt
tttcaagagtgccatgcccgaaggttatgtacaggaaagaactatatttttcaaagatgacgggaactacaagacacgtg
gfp ctgaagtcaagtttgaaggtgatacccttgttaatagaatcgagttaaaaggtattgattttaaagaagatggaaacattctt
ggacacaaattggaatacaactataactcacacaatgtatacatcatggcagacaaacaaaagaatggaatcaaagtta
acttcaaaattagacacaacattgaagatggaagcgttcaactagcagaccattatcaacaaaatactccaattggcgat
ggccctgtccttttaccagacaaccattacctgtccacacaatctgccctttcgaaagatcccaacgaaaagagagacca
catggtccttcttgagtttgtaacagctgctgggattacacatggcatggatgaactatacaaataa
atggcgagcttcctgaaaaagacgatgccgttcaaaaccacgattgaaggcacggtcaacggtcactactttaaatgta
cgggtaaaggcgaaggtaacccgttcgaaggcacccaggaaatgaaaattgaagtcatcgaaggcggtccgctgcc
gtttgcgtttcatattctgagcacgtcttgcatgtacggctcaaagaccttcatcaagtacgtgtcgggtatcccggattact
ttaaacagagcttcccggaaggctttacctgggaacgtaccacgacctatgaagatggcggtttcctgacggcgcacca
pink agacacctctctggatggtgactgtctggtgtacaaagttaagattctgggcaacaattttccggccgatggtccggttat
gcagaacaaagcgggccgttgggaaccggctacggaaatcgtctatgaagtggacggcgttctgcgcggtcaatccc
tgatggccctgaaatgcccgggcggtcgtcatctgacctgtcatctgcacacgacctatcgtagcaaaaaaccggcga
gcgccctgaaaatgccgggctttcatttcgaagatcaccgcattgaaatcatggaagaagtcgaaaaaggtaagtgcta
caagcagtacgaagcagcagtgggtcgctattgtgatgcggcaccgagcaagctgggtcataattaa
atgtctaaaatgcaggaattcgctcgtctggaaaccgttgcttctatgcgtcgtgctgtttgggacggtaacgaatgccag
ccgggtaaagttgctgacgttgttctgcgttcttggacccgttgccgtgctgaaggtgttgttccgaacgctcgtcaggaa
ttcgacccgatcccgcgtaccgctctggacgaaaccgttgaagctaaacgtgctctgatcctggctgctgaaccggttgt
tgacgctctgatggaacagatgaacgacgctccgcgtatgatcatcctgaacgacgaacgtggtgttgttctgctgaacc
agggtaacgacaccctgctggaagacgctcgtcgtcgtgctgttcgtgttggtgtttgctgggacgaacacgctcgtggt
bmoR accaacgctatgggtaccgctctggctgaacgtcgtccggttgctatccacggtgctgaacactacctggaatctaacac
catcttcacctgcaccgctgctccgatctacgacccgttcggtgaattcaccggtatcctggacatctctggttacgctggt
gacatgggtccggttccgatcccgttcgttcagatggctgttcagttcatcgaaaaccagctgttccgtcagaccttcgct
gactgcatcctgctgcacttccacgttcgtccggacttcgttggtaccatgcgtgaaggtatcgctgttctgtctcgtgaag
gtaccatcgtttctatgaaccgtgctggtctgaaaatcgctggtctgaacctggaagctgttgctgaccaccgtttcgactc
tgttttcgacctgaactttggcgcgttcctggaccacgttcgtcagtctgctttcggtctggttcgtgtttctctgtacggtggt

gttcaggtttacgctcgtgttgaaccgggtctgcgtgttccgccgcgtccggctgctcacgctcgtccgccgcgtccgg
ctccgcgtccgctggactctctggacaccggtgacgctgctgttcgtctggctatcgaccgtgctcgtcgtgctatcggt
cgtaacctgtctatcctgatccagggtgaaaccggtgctggtaaagaagttttcgctaaacacctgcacgctgaatctcc
gcgttctaaaggtccgttcgttgctgttaactgcgctgctatcccggaaggtctgatcgaatctgaactgttcggttacgaa
gaaggtgctttcaccggtggtcgtcgtaaaggtaacatcggtaaagttgctcaggctcacggtggtaccctgttcctgga
cgaaatcggtgacatggctccgggtctgcagacccgtctgctgcgtgttctgcaggaccgtgctgttatgccgctgggt
ggtcgtgaaccgatgccggttgacatagcgctggtctgcgcaacccaccgtaacctgcgttctctgatcgctcagggtc
agttccgtgaagacctgtactaccgtctgaacggtctggctatctctctgccgccgctgcgtcagcgttctgacctggctg
ctctggttaaccacatcctgttccagtgctgcggtggtgaaccacattactctgtaagcccggaagttatgaccctgttcaa
acgtcacgcttggccgggtaacctgcgtcagctgcacaacgttctggacgctgctctggctatgctggacgacggtcac
gttatcgaaccgcaccacctgccggaagacttcgttatggaagttgactctggtctgcgtccgatcgaagaagacggttc
taccgctgctcaccgtgctcgtcagccggcttctggttctggtccggctaaaaaactgcaggacctggctctggacgcta
tcgaacaggctatcgaacagaacgaaggtaacatctctgttgctgcgcgtcagctgggtgtaagccgtaccaccatcta
ccgtaaactgcgtcagctgtctccgaccggttgccaccgtccggctcactggtctcagtctcgtatcggtacctaa
atgtctaaaatgcaggaattcgctcgtctggaaaAcgttgcttctatgcgtcgtgctgtttgggacggtaacgaatgcca
gccgggtaaagttgctgacgttgttctgcgttcttggacccgttgccgtgctgaaggtgttgttccgaacgctcgtcagga
attcgacccgatcccgcgtaccgctctggacgaaaccgttgaagctaaacgtgctctgatcctggctgctgaaccggtt
gttgacgctctgatggaacagatgaacgacgctccgcgtatgatcatcctgaacgacgaacgtggtgttgttctgctgaa
ccagggtaacgacaccctgctggaagacgctcgtcgtcgtgctgttcgtgttggtgtttgctgggacgaacacgctcgt
ggtaccaacgctatgggtaccgctctggctgaacgtcgtccggttgctatccacggtgctgaacactacctggaatctaa
caccatcttcacctgcaccgctgctccgatctacgacccgttcggtgaattcaccggtatcctggacatctctggttacgc
tggtgacatgggtccggttccgatcccgttcgttcagatggctgttcagttcatcgaaaaccagctgttccgtcagaccttc
gctgactgcatcctgctgcacttccacgttcgtccggacttcgttggtaccatgcgtgaaggtatcgctgttctgtctcgtg
aaggtaccatcgtttctatgaaccgtgctggtctgaaaatcgctggtctgaacctggaagctgttgctgaccaccgtttcg
actctgttttcgacctgaactttggcgcgttcctggaccacgttcgtcagtctgctttcggtctggttcgtgtttctctgtacg
gtggtgttcaggtttacgctcgtgttgaaccgggtctgcgtgttccgccgcgtccggctgctcacgctcgtccgccgcgt
bmoR^{T12N} ccggctccgcgtccgctggactctctggacaccggtgacgctgctgttcgtctggctatcgaccgtgctcgtcgtgctat
cggtcgtaacctgtctatcctgatccagggtgaaaccggtgctggtaaagaagttttcgctaaacacctgcacgctgaat
ctccgcgttctaaaggtccgttcgttgctgttaactgcgctgctatcccggaaggtctgatcgaatctgaactgttcggtta
cgaagaaggtgctttcaccggtggtcgtcgtaaaggtaacatcggtaaagttgctcaggctcacggtggtaccctgttcc
tggacgaaatcggtgacatggctccgggtctgcagacccgtctgctgcgtgttctgcaggaccgtgctgttatgccgct
gggtggtcgtgaaccgatgccggttgacatagcgctggtctgcgcaacccaccgtaacctgcgttctctgatcgctcag
ggtcagttccgtgaagacctgtactaccgtctgaacggtctggctatctctctgccgccgctgcgtcagcgttctgacctg
gctgctctggttaaccacatcctgttccagtgctgcggtggtgaaccacattactctgtaagcccggaagttatgaccctg
ttcaaacgtcacgcttggccgggtaacctgcgtcagctgcacaacgttctggacgctgctctggctatgctggacgacg
gtcacgttatcgaaccgcaccacctgccggaagacttcgttatggaagttgactctggtctgcgtccgatcgaagaaga
cggttctaccgctgctcaccgtgctcgtcagccggcttctggttctggtccggctaaaaaactgcaggacctggctctgg
acgctatcgaacaggctatcgaacagaacgaaggtaacatctctgttgctgcgcgtcagctgggtgtaagccgtaccac
catctaccgtaaactgcgtcagctgtctccgaccggttgccaccgtccggctcactggtctcagtctcgtatcggtacctaa

a

atgtctaaaatgcaggaattcgctcgtctggaaaccgttgcttctatgcgtcgtgctgtttgggacggtaacgaatgccag
ccgggtaaagttgctgacgttgttctgcgttcttggacccgttgccgtgctgaaggtgttgttccgaacgctcgtcaggaa
ttcgacccgatcccgcgtaccgctctggacgaaaccgttgaagctaaacgtgctctgatcctggctgctgaaccggttgt
tgacgctctgatggaacagatgaacgacgctccgcgtatgatcatcctgaacgacgaacgtggtgttgttctgctgaacc
agggtaacgacaccctgctggaagacgctcgtcgtcgtgctgttcgtgttggtgtttgctgggacgaacacgctcgtggt
accaacgctatgggtaccgctctggctgaacgtcgtccggttgctatccacggtgctgaacactacctggaatctaacac
catcttcacctgcaccgctgctccgatctacgacccgttcggtgaattcaccggtatcctggacatctctggttacgctggt
gacatgggtccggttccgatcccgttcgttcagatggctgttcagttcatcgaaaaccagctgttccgtcagaccttcgct
gactgcatcctgctgcacttccacgttcgtccggacttcgttggtaccatgcgtgaaggtatcgctgttctgtctcgtgaag
gtaccatcgtttctatgaaccgtgctggtctgaaaatcgctggtctgaacctggaagctgttgctgaccaccgtttcgactc
tgttttcgacctgaactttggcgcgttcctggaccacgttcgtcagtctgctttcggtctggttcgtgtttctctgtacggtggt
gttcaggtttacgctcgtgttgaaccgggtctgcgtgctccgccgcgtccggctgctcacgctcgtccgccgcgtccgg
BmoR^{V311A} ctccgcgtccgctggactctctggacaccggtgacgctgctgttcgtctggctatcgaccgtgctcgtcgtgctatcggt
cgtaacctgtctatcctgatccagggtgaaaccggtgctggtaaagaagttttcgctaaacacctgcacgctgaatctcc
gcgttctaaaggtccgttcgttgctgttaactgcgctgctatcccggaaggtctgatcgaatctgaactgttcggttacgaa
gaaggtgctttcaccggtggtcgtcgtaaaggtaacatcggtaaagttgctcaggctcacggtggtaccctgttcctgga
cgaaatcggtgacatggctccgggtctgcagacccgtctgctgcgtgttctgcaggaccgtgctgttatgccgctgggt
ggtcgtgaaccgatgccggttgacatagcgctggtctgcgcaacccaccgtaacctgcgttctctgatcgctcagggtc
agttccgtgaagacctgtactaccgtctgaacggtctggctatctctctgccgccgctgcgtcagcgttctgacctggctg
ctctggttaaccacatcctgttccagtgctgcggtggtgaaccacattactctgtaagcccggaagttatgaccctgttcaa
acgtcacgcttggccgggtaacctgcgtcagctgcacaacgttctggacgctgctctggctatgctggacgacggtcac
gttatcgaaccgcaccacctgccggaagacttcgttatggaagttgactctggtctgcgtccgatcgaagaagacggttc
taccgctgctcaccgtgctcgtcagccggcttctggttctggtccggctaaaaaactgcaggacctggctctggacgcta
tcgaacaggctatcgaacagaacgaaggtaacatctctgttgctgcgcgtcagctgggtgtaagccgtaccaccatcta
ccgtaaactgcgtcagctgtctccgaccggttgccaccgtccggctcactggtctcagtctcgtatcggtacctaa
atggttcgtccggcttctcgttctgaacgtgcttctctgcgtatcatgaacatcaacccgtggcgttgcgaagtttctgaaa
aactgctgaaacaggttaacgacaaagtttctctgtgcgaaaaaatcgctgctgaaggtttctctttcgttccgggtcagc
agatccgtgaatggctgctggcttctaactctgaagctctgcaggactggcaggctttcgacgcttcttgggacggtatg
ccgctggacgaatacatggctgacggtggtcgttaccgtcgtcgtcgtttcgctaccctgtctgctgctaccgaaggtcc
gatcatgctggaaccgcaccagccgcactaccagtctcgtgaatacaactctctgaacggtggtatcgctcgtatctacg
mfl aaccgatcccgccggctgttatccagggtcagaccatgcagtctatcctgcagctgtctcgtgacctgttctctcacctgc
gtccgcagacccgttggcacatcgaagctcaccagttccgtatcgaaaccaaccagcacgaacgtggtcagccggct
ccggaaggtgttcaccgtgacggtgttgactacgttctggttatgatggttaaacgtgttaacatctcttctggtaccacca
ccctgcacaacctggacaaagttgttctggactctttcaccctgaccaacccgctggactgcgctctggttgacgaccgt
cgttgcatgcacggtgttaccccggttgaacagatcgacccggctaaagctgcttaccgtgacgttctggttgttaccttc
accgctaaactgtaa

aad	atggctatctctcgtcgtaaattcatcatcggtggtaccgttgttgctgttgctgctggtgctggtatcctgaccccgatgct<br>gacccgtgaaggtcgtttcgttccgggtaccccgcgtcacggtttcgttgaaggtaccgaaggtgctctgccgaaacag<br>gctgacgttgttgttgttggtgctggtatcctgggtatcatgaccgctatcaacctggttgaacgtggtctgtctgttgttatc<br>gttgaaaaaggtaacatcgctggtgaacagtcttctcgtttctacggtcaggctatctcttacaaaatgccggacgaaacc<br>ttcctgctgcaccacctgggtaaacaccgttggcgtgaaatgaacgctaaagttggtatcgacaccacctaccgtaccc<br>agggtcgtgttgaagttccgctggacgaagaagacctggttaacgttcgtaaatggatcgacgaacgttctaaaaacgtt<br>ggttctgacatcccgttcaaaacccgtatcatcgaaggtgctgaactgaaccagcgtctgcgtggtgctaccaccgact<br>ggaaaatcgctggtttcgaagaagactctggttctttcgacccggaagttgctaccttcgttatggctgaatacgctaaaa<br>aaatgggtgttcgtatctacacccagtgcgctgctcgtggtctggaaacccaggctggtgttatctctgacgttgttaccg<br>aaaaaggtgctatcaaaacctctcaggttgttgttgctggtggtgtttggtctcgtctgttcatgcagaacctgaacgttgac<br>gttccgaccctgccggcttaccagtctcagcagctgatctctggttctccgaccgctccgggtggtaacgttgctctgcc<br>gggtggtatcttcttccgtgaacaggctgacggtacctacgctacctctccgcgtgttatcgttgctccggttgttaaagaa<br>tctttcacctacggttacaaatacctgccgctgctggctctgccggacttcccggttcacatctctctgaacgaacagctg<br>atcaactctttcatgcagtctacccactggaacctggacgaagtttctccgttcgaacagttccgtaacatgaccgctctgc<br>cggacctgccggaactgaacgcttctctggaaaaactgaaagctgaattcccggctttcaaagaatctaaactgatcga<br>ccagtggtctggtgctatggctatcgctccggacgaaaacccgatcatctctgaagttaaagaatacccgggtctggtta<br>tcaacaccgctaccggttggggtatgaccgaatctccggtttctgctgaactgaccgctgacctgctgctgggtaaaaaa<br>ccggttctggacccgaaaccgttctctctgtaccgtttctaa
cipA	atgatcaacgacatgcacccgtctctgatcaaagacaaagacatcgttgacgacgttatgctgcgttcttgcaaaatcatc<br>gctatgaaagttatgccggacaaagttatgcaggttatggttaccgttctgatgcatgatggtgtctgcgaagagatgctg<br>ctgaaatggaacctgctggacaaccgtggtatggctatctacaaagttctgatggaagctctgtgcgctaaaaaagacgt<br>taaaatctctaccgttggtaaagttggtccgctgggttgcgactacatcaactgcgttgaaatctctatgtaa

Table s5 sgRNA and donor sequences used in this study

Target genes	sgRNA sequence	Donor sequence
lacIZYA	ctgacaatggcagatcccag	5HA<br>cggttccttactggcattgatgacgcttgggcaagccgcaggagctttgctgatgcctgctatggctcgccat

		caggatcggcgcaaactgttaatgctggcgctggtgttacaactggtggggttctgcggctttatctggctgc<br>cgatgcaattgccggtattgtgggcgatggtgtgtgggttaggtctgggcggcgcgtttccgctctgtttgct<br>gctggcgctcgatcactctgtgcaaccggctattgctggcaagctggtggcgtttatgcagggaatcggtttt<br>atcatcgccgggcttgccccgtggttttctggcgtgctgcgtagtatcagcggcaattacctgatggactgg<br>gcatttcatgcgctgtgcgtcgttgggctgatgatcataaccctgcgttttgcaccagtacgttttccgcagct<br>gtgggtcaaagaggcatgatgcgacgcttgttcctgcgctttgttcatgccggatgcggctaatgtagatcg<br>ctgaacttgtaggcctgataagcgcagcgtatcaggcaatttttataatttaaactgacgattcaactttataatc<br>tttgaaataatagtgcttatcccggtcgtttatttcgc<br>3HA<br>attcaccaccctgaattgactctcttccgggcgctatcatgccataccgcgaaaggttttgcgccattcgatg<br>gtgtcaacgtaaatgcatgccgcttcgccttccggccaccagaatagcctgcgattcaaccccttcttcgatc<br>tgttttgctacccgttgtagcgccggaagatgcttttccgctgcctgttcaatggtcattgcgctcgccatatac<br>accagattcagacagccaatcacccgttgttcactgcgcagcggtacggcgatagaggcgatcttctcctc<br>ctgatcccagccgcggtagttctgtccgtaaccctctttgcgcgcgcgcgccagaatggcttccagctttaa<br>cggttcccgtgccagttgatagtcatcaccggggcgggaggctaacatttcgattaattccttgcggtcttgtt<br>ccgggcaaaaggccagccaggtcaggcccgaggcggttttcagaagcggcaaacgtcgcccgaccatt<br>gcccggtgaaaggataagcggctgaaacggtgagtggtttcgcgtaccaccattgcatcaacatccagcgt<br>ggacacatctgtcggccata<br>5HA<br>agacctacgacgtcatcaagtatttcaacatgatggatgaaggcaaagtcaccggttatttctgccagggctt<br>taacccggttgcgtccttcccggacaaaaacaaagtggtgagctgcctgagcaagctgaagtacatggtgg<br>ttatcgatccgctggtgactgaaacctctaccttctggcagaaccacggtgagtcgaacgatgtcgatccgg<br>cgtctattcagactgaagtattccgtctgccttcgacctgctttgctgaagaagatggttctatcgctaactccg<br>gtcgctggttgcagtggcactggaaaggtcaggacgcgccgggcgaagcgcgtaacgacggtgaaattc<br>tggcgggtatctaccatcatctgcgcgagctgtaccagtccgaaggtggtaaaggcgtagaaccgctgatg<br>aagatgagctggaactacaagcagccgcacgaaccgcaatctgacgaagtggctaaagagaa
98k-1	gaagtggctaaagagaacaa;<br>cagcgtgtcacatatgaagg	3HA<br>ctgccacttatcgcgtgaatcctgataaagttcatgcttttccgggtctggtgtgtgcgtccgttcccagcgaa<br>ccaggcgttctccggtttctgccattgatgaaaaaattccggcaccgacgccagctgcaatggcacatccta<br>atgcagtggcttctttgaccaccggaatattgacgggtaatcccgagacatcagcgagaatttgactccataa<br>tttcccttttgaacctccgcctgcaaagactaacgatgaaggatgaatattcgagaaatcagcaatttgctgca<br>agttacacgctgatacaatcgccgcattttcttccagcgcacggaacaatgtcgctttgttacatttatccgggt<br>caatggacaagttaataaaggaaggcgcagcgtgataccaggttttaaagcgcattctgtcggagaagatc<br>ggcattacgccccacgacccaggcggcacccgactggccatctcttccagcagcgtataggtgtcgatgc<br>ctaaacgttcg

98k-2	cagtttatcaggatcagctg;<br>aaaaaccgccagtaaaccgg	5HA<br>gaggtgggaaggtgtcaatttggctaatttgctaatgctttcgaattgatagattaaaaattgcatagttcttattt<br>tatttaaatatgaactatgagttattgttgcttaataagaaaactgtttctttcaataggaaaattatcatcatcatat<br>ttaacaaagaatagcactaattgctaaaaatcgaagtttattaaaccccttttgcatccattagtgtctttaggaa<br>tattcgctataaaataagggttgtgcttaatgctttaagaaaaatagcaattttcccttgaatatcgtactggtgat<br>ggaacgatgaatctgcaagctggcttttaacaagccagctctaaaagaagggaaataagaataactatactc<br>aaaaactaacagccacggtcatcatgatgtggctgtcaatgaaactataacccaggcgctttccataacgac<br>gtcgtcagtccatgatcaaccagcc<br>3HA<br>cggtgaatgcttgcatggatagatttgtgttttgcttttacgctaacaggcattttcctgcactgataacgaatcg<br>ttgacacagtagcatcagttttctcaatgaatgttaaacggagcttaaactcggttaatcacattttgttcgtcaa<br>taaacatgcagcgatttcttccggtttgcttaccctcatacattgcccggtccgctcttccaatgaccacatcca<br>gaggctcttcaggaaatgcgcgactcacacctgctgtcacggtaatgttgatatgcccttcagaatgtgtgat<br>ggcatggttatcgactaactggcaaattctgacacctgcacgacatgcttcttcatcattagccgctttgacaa<br>taatgataaattcttcgcccccgtagcgataaaccgtttcgtaatcacgcgtccaactggctaagtaagttgcc<br>agggtgcgtaatactacatcgccgattaaatgccc
98k-3	aaaaaccgccagtaaaccgg;<br>cagcgtgtcacatatgaagg	5HA<br>ccgccagaaatgaaagccagctatttttccgcccagtctttaggctggcttggtgccgcgattaacccattag<br>tgagtggcgtagtgctaaccagcctgccgccttcctcgctgtttgtcatcttagcgttggtgatcattgctgcgt<br>gggtgctgatgttaaaagggattcgagcaagaccgtgggggcagcccgcgctttgttgatttaagtcgaac<br>acaataaagatttaattcagccttcgtttaggttacctctgctaatatctttctcattgagatgaaaattaaggtaa<br>gcgaggaaacacaccacaccataaacggaggcaaataatgctgggtaatatgaatgtttttatggccgtact<br>gggaataattttattttctggttttctggccgcgtatttcagccacaaatgggatgactaatgaacggagataat<br>ccctcacctaaccggccccttgttacagttgtgtacaaggggc<br>3HA<br>tcatatgtgacacgctgaacatcgatgttcgggcgctctttcaaagcccagaatgcttcagtgattaatatcgt<br>cgcctgctcctgtgtcattcctggtcgacatatccaggcatccagagcctcacgagcctgttcaggagtgatt<br>ttcattgttcaaccgccccgcccgcttcgtcttacgatattcatcataaactttgggatcatactgaagctcccc<br>gccagatgcctcctgtagacgcatcgcgcgaccttcgggaactaaatcccctttccagctataaagcgaag<br>ccaaacgaatacctgctgcttgtgcaagttttgtttttgaaccgaaatacaaaagagcgtcagttttaagcattt<br>aaaacacctttattgttagtcataactaacaagatagatgttaacaaaaacatagtcaatacgatttagcattag<br>ctaactatggaaacaaaaaatttaactatcggcgaacgcatcaggtatcgtcg
98k-2-1	tattcaggccatcaccccag	5HA<br>gaggtgggaaggtgtcaatttggctaatttgctaatgctttcgaattgatagattaaaaattgcatagttcttattt<br>tatttaaatatgaactatgagttattgttgcttaataagaaaactgtttctttcaataggaaaattatcatcatcatat

		ttaacaaagaatagcactaattgctaaaaatcgaagtttattaaaccccttttgcatccattagtgtctttaggaa<br>tattcgctataaaataagggttgtgcttaatgctttaagaaaaatagcaattttcccttgaatatcgtactggtgat<br>ggaacgatgaatctgcaagctggcttttaacaagccagctctaaaagaagggaaataagaataactatactc<br>aaaaactaacagccacggtcatcatgatgtggctgtcaatgaaactataacccaggcgctttccataacgac<br>gtcgtcagtccatgatcaaccagcc<br>3HA<br>ccgtaccttatgtgcgctctatgccatgacggggctggcgtctgcggtcgccgctgtgctgctggtatcgtat<br>tttggttcagcacgttccgatctcggtgcgtcgtttctgatgcccgccatcaccgccgtggtgcttggcgggg<br>ccaatatttatggtggttccggttccattatcggcaccgccattgcggttttattagtgggatatttgcaacaag<br>gtttgcaaatggcaggagtgccaaatcaggtgtccagcgccctttccggtgcgctacttatcgtcgttgtcgt<br>aggtcgttccgttagcctgcatcgccagcaaattaaagagtggctggcgcgtcgggccaataacccattgc<br>cataaaggatatcttcatgacacttcatcgctttaagaaaatcgccttacttagcgctcttggcattgccgcaat<br>ctctatgaatgtgcaggccgcagagcgtattgcatttattcccaaactggttggcg
98k-2-2	atacgataccagcagcacag	5HA<br>tgttcagcaccagtgactttatctgcattggcattgtcgccctaccgctaacgatggtgattgtcagtggcggg<br>atcgatatttcgtttggttcgaccatcggcctctgcgccattgcattgggcgtactgtttcaaagtggtgtgccg<br>atgccgctggcgatactcctgaccttactgctcggcgcattgtgcgggctgatcaacgccggattaattatct<br>ataccaaagttaacccgctggtgattacgcttggcacgctgtatctgtttgccggaagcgctctgctgctttcc<br>ggtatggccggagcgacggggtacgaaggtattggtggattcccgatggcgtttacagatttcgctaacct<br>ggatgtgctgggactccccgttccgctgattatcttcctgatatgtctcctcgttttctggctctggctgcataaa<br>acccatgccggacgtaatgtgtttttgattgggcaaagcccgcgcgtggcgctttatagcgcgattccagtta<br>a<br>3HA<br>agtgttttcacaatgggttcccttctgattagtcatacaacctgtttgaattggtacgacaggttagcaaacttta<br>atacgccgaacccctgttttgatcaactcctgatgattaatgagcagttttatgagaaaagtgtggcgcggatc<br>atggtttaatcgaggaaaaacgccttttcctggatcataaagtggtagaacacattgcattcaaatcgcgcgt<br>aatgaataaagatgtcagacaacttcctcaccgtaacgcatagtgctggtacggttgcgcccatctttcttcga<br>gcgatacagacacgtatcggcttcgaccatcaatttattaaagtcatcggtcagcgttaggtacgatgcgcg<br>accactaccgacgccaatacttaccgtgagataaagcgtttttttgttgccaggtgaatggttgcagttcaacg<br>cctttacggatttttttccgccattagcagaccatcgacaggattcaccgacggcactgcaacagcaaattctt<br>cg
98k-2-3	tcgcttaacgaaacaccctg	5HA<br>acggaatgatgatccaacctttatccagcgcaccgttgaaatggctaaagcgttcgaacagcagacgggtc<br>agttttgccggatagcttaccgctggcgcgtcatcgaatttatcgcccgcatggtagctgatacccgcttcgg<br>tggtgttagagaaaacaaagcgcatttccgggttgtgcgccagtttcaggaattcatcgtattcactgtagac<br>gctgatttcacgattaaccgagcgaatcagacgcgcgtcgctgaccgcttccccccttctcattcaggccacg<br>gataatggtggtgtacagaccatcctgcgtgctcagtgacggcgggaatgaagtttcaatcggacgaacaa<br>cgaccacgccagaattcagatcggtgtgctcattcaggagatcgatttgccagtcaacaaaggcgcgcag<br>gaagttaccttcaccaaactgaatgatacgttctggatactgtgcaccgggaaaatcgcgacgattt

		3HA<br>gtgatttgaatgacgtgttgatgccagcatcattaagtgatgataaaattatcaaacatatggcgcgcaggac<br>cctcaaactgtttgtaaaaaatgaaacaccgccaaaagttactcacgaagacctggtgaaaaacaggcctc<br>gctcccctgtcagaccacctatacccgcaaccgccaaaacgccagacctccctgaacgtcattaaaccgtg<br>atgttaccgactctctgacgcgtgaaagaatcagcgtcagagaaacggaaaacgcgatccagatcacaaa<br>tgcattgtattcacatcattaaccgttttaagatcatttcatcactttttcgcaactcacccgataatctgttatgac<br>aacaaacactgtttcccgcaaagtggcgtggctacgggtcgttacgctggcagtcgccgccttcatcttcaa<br>caccaccgaatttgtccctgttggcctgctctctgacattgcgcaaagttttcacatgcaaacc
98k-2-4	aatgattgaccaaattaacg	5HA<br>ctatggggcgcgtatttcagaccggcgtcgagcgtgtactctttctgtttctgaatgattttattgaacaatttcc<br>aatgatcaaccctggtgttcccatcaaaagagcgcatacaccacatattgaacccctgccttcggatcaccat<br>accgctgcagattacttacgccagtttgatttgcttgtcctgaattttatctctcgcggtaattttgtcatactcccc<br>cgattatggaataactctgaggttcacagatggttcgtcaataaagatcctaacttgatcaccgccattctcga<br>cataacggacagcgaattaaaagaggatttgttgcaaagcctgatggattcattaggttctaacaaacatgta<br>ctacccgaagtctgcatctgctttttatcccttttagcagaacaagaatctcctcattttcagaacttgtttttattttt<br>cgccaatatgttattgcactatcaccaatttatgaatcccaatgaaa<br>3HA<br>cggtgaatgcttgcatggatagatttgtgttttgcttttacgctaacaggcattttcctgcactgataacgaatcg<br>ttgacacagtagcatcagttttctcaatgaatgttaaacggagcttaaactcggttaatcacattttgttcgtcaa<br>taaacatgcagcgatttcttccggtttgcttaccctcatacattgcccggtccgctcttccaatgaccacatcca<br>gaggctcttcaggaaatgcgcgactcacacctgctgtcacggtaatgttgatatgcccttcagaatgtgtgat<br>ggcatggttatcgactaactggcaaattctgacacctgcacgacatgcttcttcatcattagccgctttgacaa<br>taatgataaattcttcgcccccgtagcgataaaccgtttcgtaatcacgcgtccaactggctaagtaagttgcc<br>agggtgcgtaatactacatcgccgattaaatgcccgtag

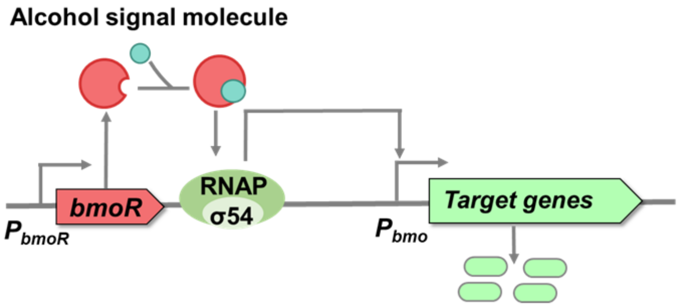

Figure s1. The transcription activation mechanism of BmoR-based regulation system. BmoR reshapes the structure of RNA polymerase holoenzyme ($E\sigma^{54}$) by sensing alcohol molecules, in order to activate the transcription of target genes downstream of BmoR-regulated σ^{54}-dependent promoter P_{bmo}.

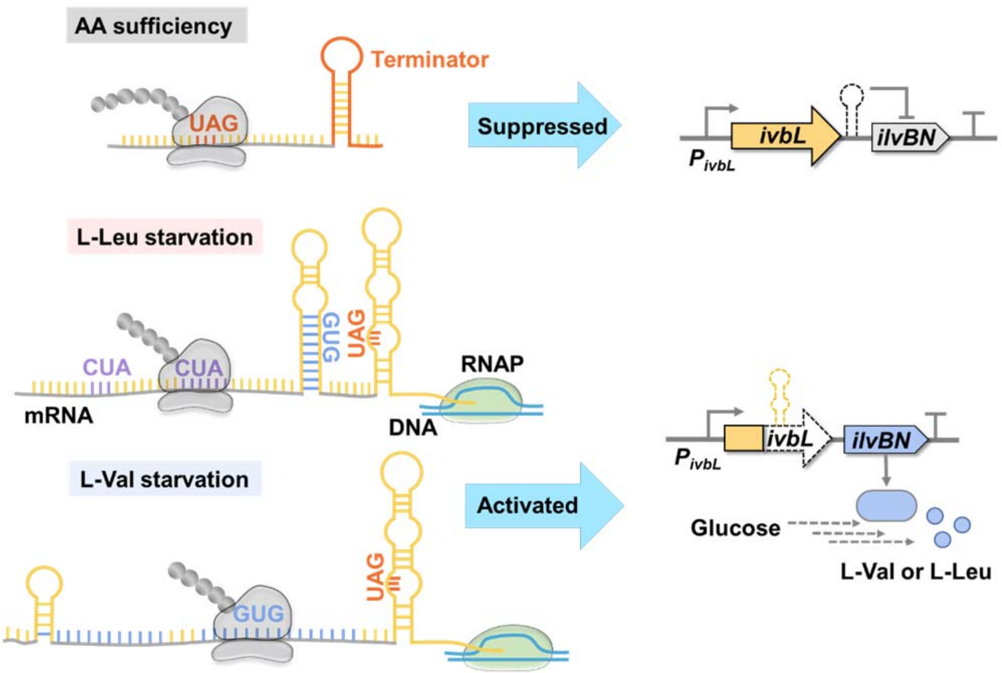

Figure s2. The transcription attenuation mechanism of *ivbL* system in *E. coli*.

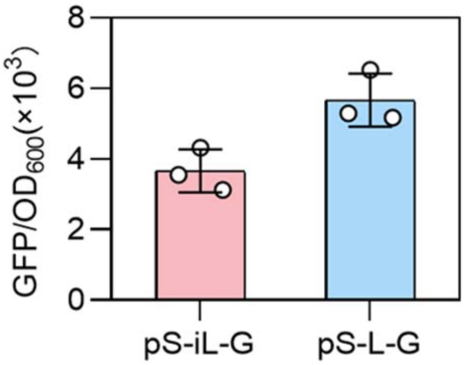

Figure s3. Disclosure expression level of *ivbL*-dependent inducible cascade system. *E. coli* MG1655Δ*lacIZYA* strain harboring pS-iL-G and *E. coli* MG1655Δ*lacIZYA* harboring pS-L-G were used. The culture was without addition of AA. Medium M9NY was used. Values and error bars represent mean and s.d. (n = 3), respectively.

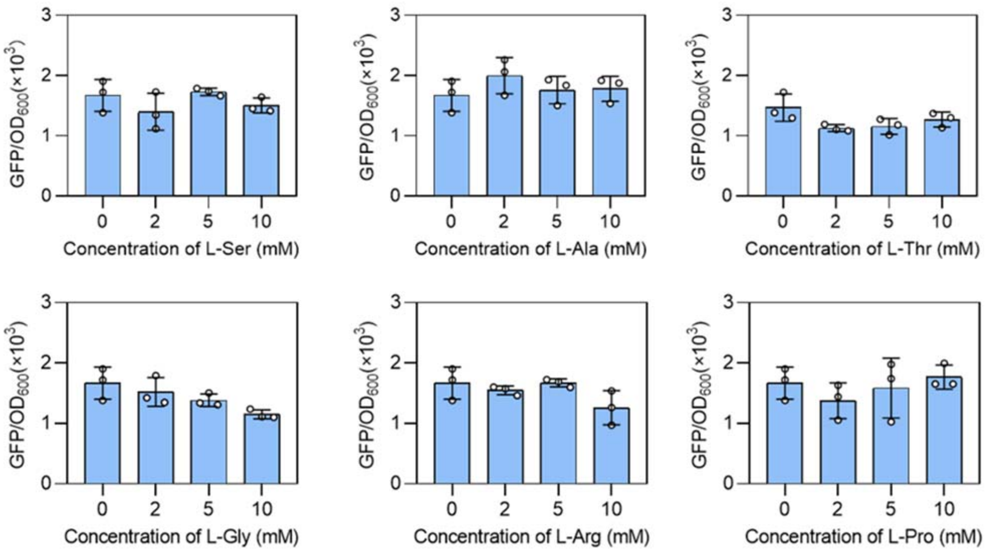

Figure s4. The response of *ivbL*-dependent inducible cascade activation system to L-Ser, L-Ala, L-Thr, L-Gly, L-Arg or L-Pro. Values and error bars represent mean and s.d. (n = 3), respectively.

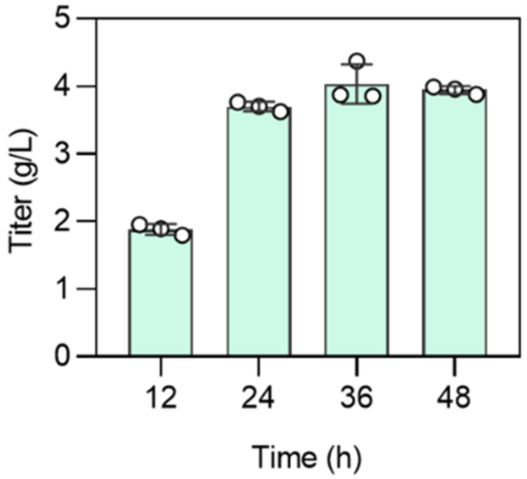

Figure s5. The titer of L-Val. The L-Val-producing strain stored in our lab was used. Values and error bars represent mean and s.d. (n = 3), respectively.

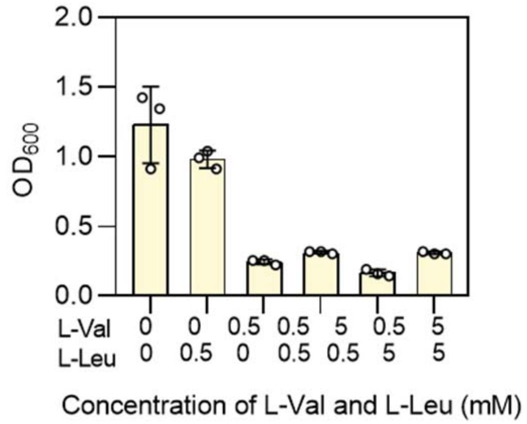

Figure s6. The OD_{600} values of the strain that contained the two-layer cascade system in Figure 3b. Values and error bars represent mean and s.d. (n = 3), respectively.

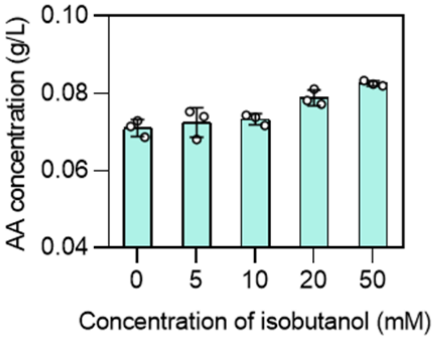

Figure s7. The AA concentration of the strain that contained the two-layer cascade system in Figure 3e. Values and error bars represent mean and s.d. (n = 3), respectively.

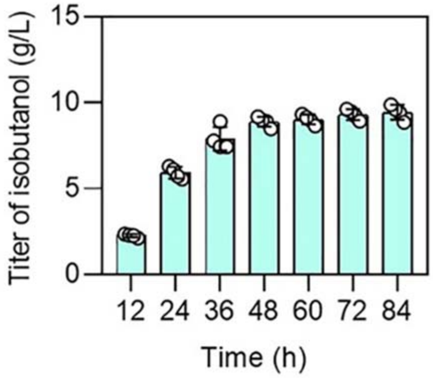

Figure s8. The CRUISE containing BmoR^{T12N} to regulate isobutanol production. *E. coli* MG1655Δ*lacIZYA* strain harboring pS-B^{T12N}-AII and pS-iL-LKY was used. Values and error bars represent mean and s.d. (n = 3), respectively.

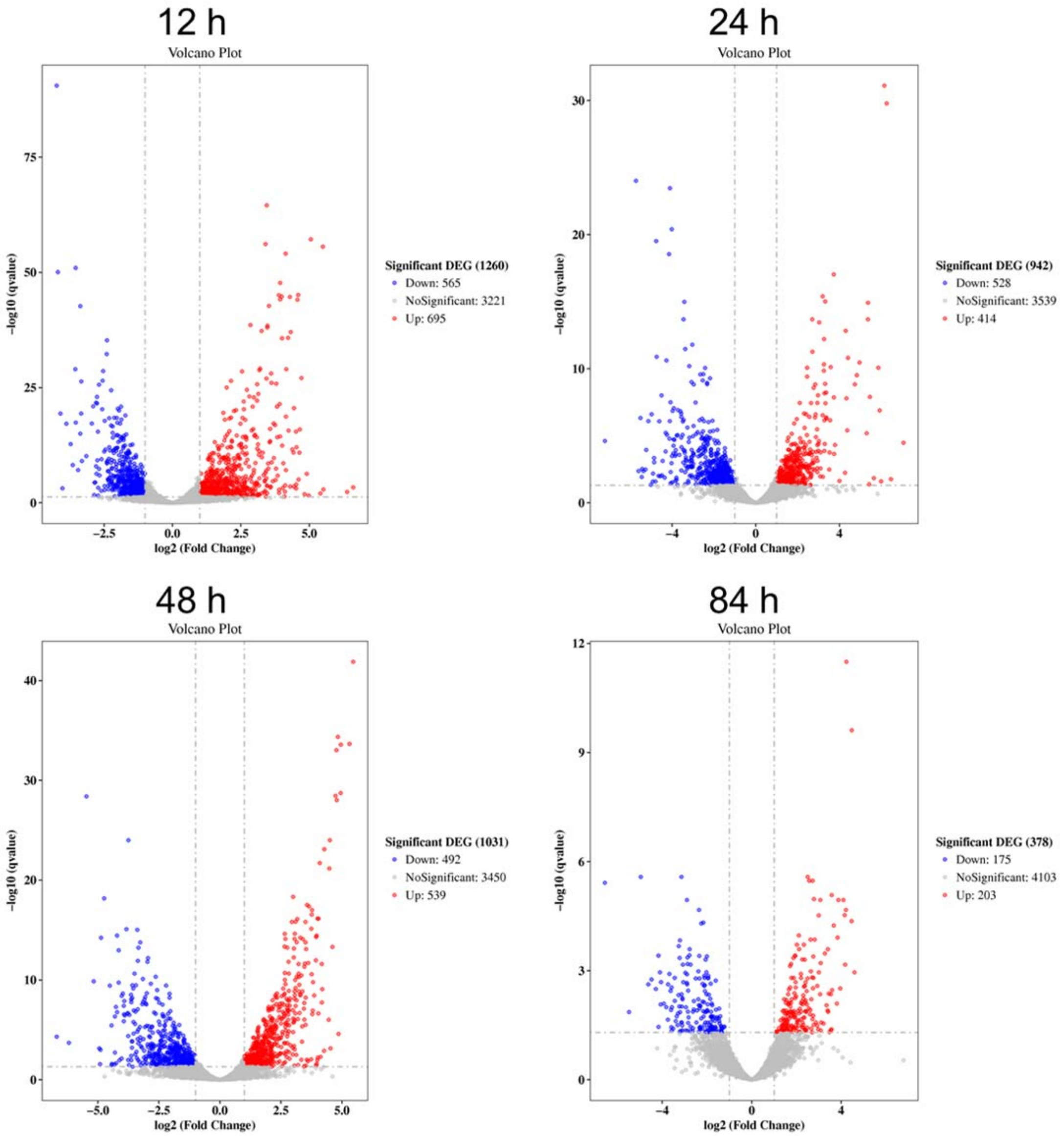

Figure s9. The number of differential genes between the experimental strain and the control strain at 12, 24, 48 and 84 h.

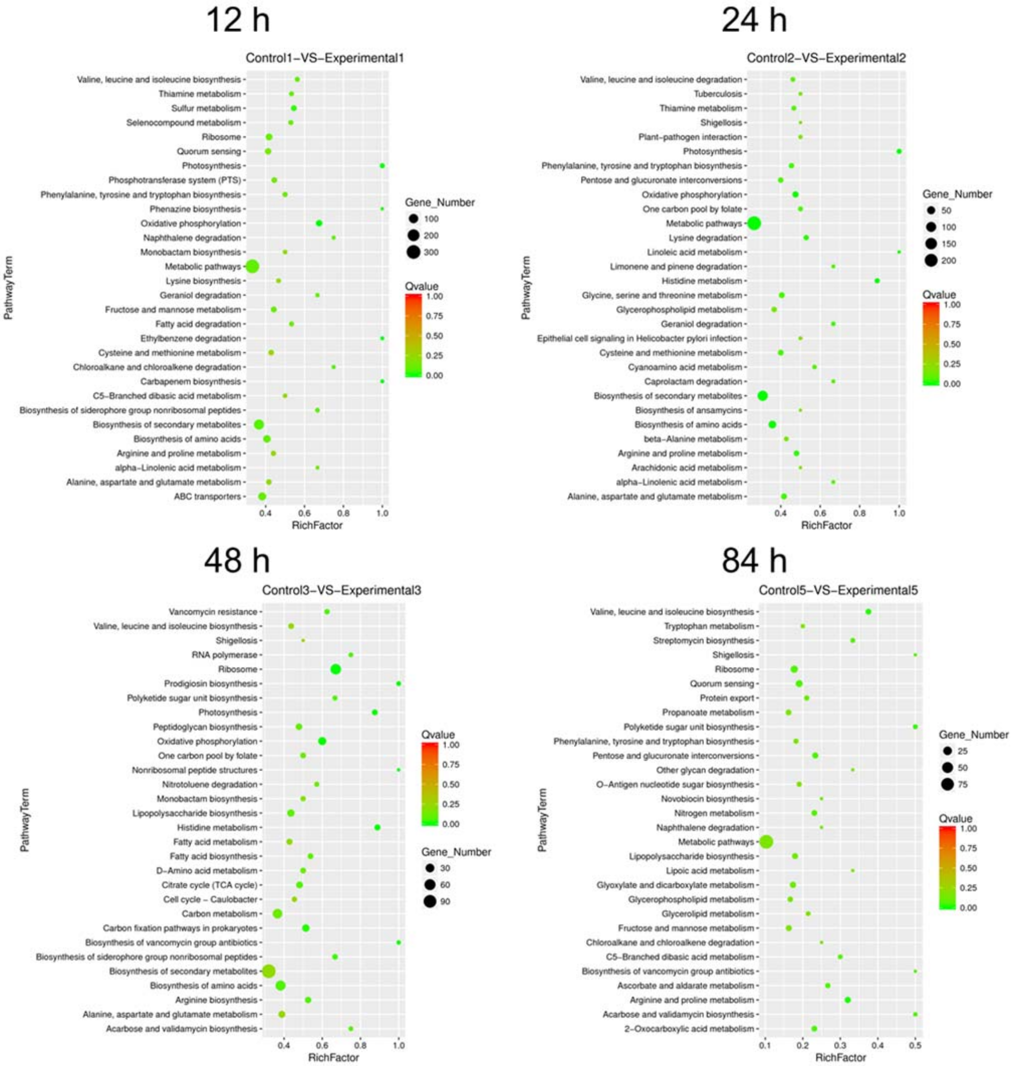

Figure s10. The ratio of the number of differential genes annotated in the pathway to the total number of annotated genes in the pathway at 12, 24, 48 and 84 h.

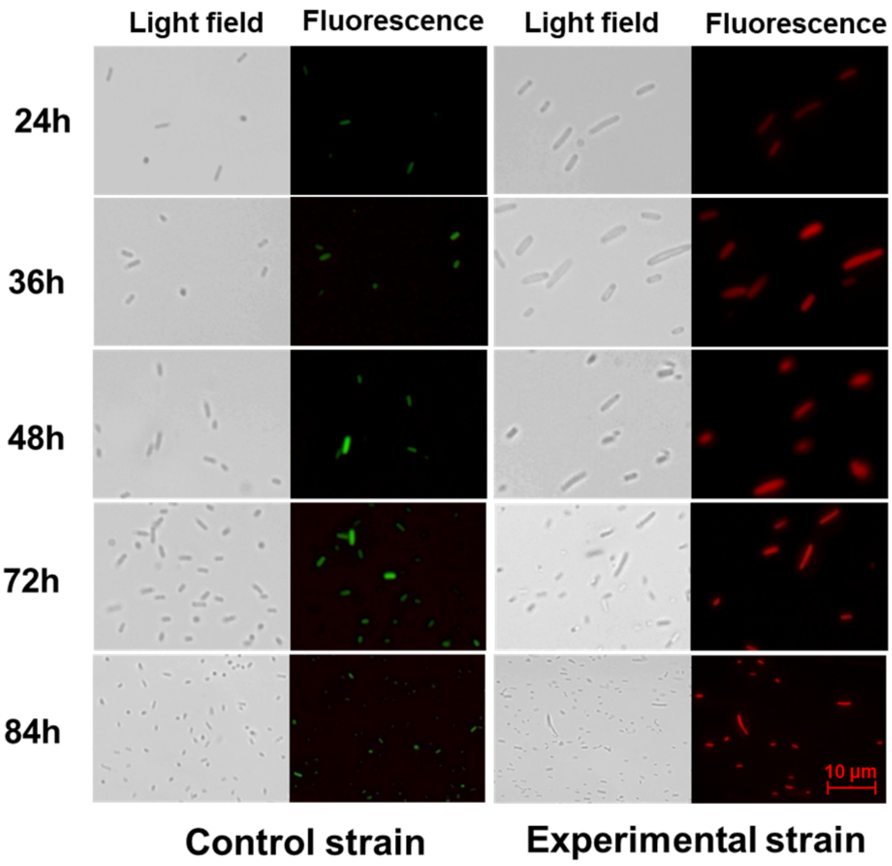

Figure s11. The fluorescence intensity of strains throughout the fermentation process under fluorescence microscopy. *E. coli* MG1655Δ*lacIZYA* strain harboring pSA69-R and pCS97-G was used as control strain, while *E. coli* MG1655Δ*lacIZYA* strain harboring pS-B-AII-R and pS-iL-LKY-G was used as experimental strain.

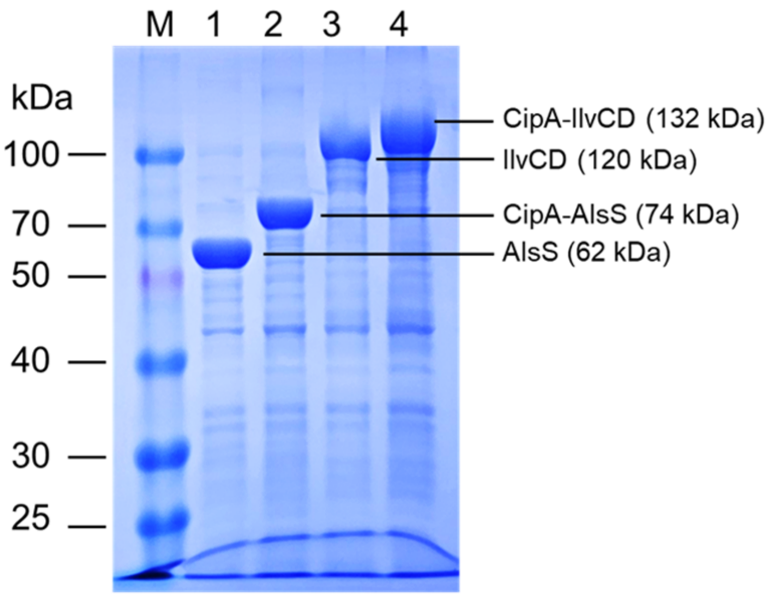

	Specific activity (μmol/min/mg protein)
**AlsS**	1.39±0.02
**CipA-AlsS**	1.24±0.04

Figure s12. SDS-PAGE of AlsS, CipA-AlsS, IlvCD and CipA-IlvCD crude extract, and the specific activity of AlsS and CipA-AlsS. M: 100 kDa protein maker. Line 1: AlsS crude extract. Line 2: CipA-AlsS crude extract. Line 3: IlvCD crude extract. Line 4: CipA-IlvCD crude extract. Values and error bars represent mean and s.d. (n = 3), respectively.

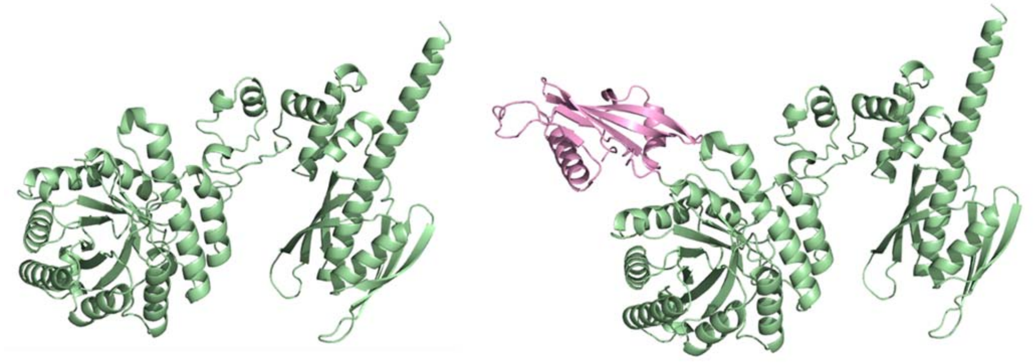

Figure s13. The structures of LeuA and CipA-LeuA. Pink represented CipA. Green represented LeuA.

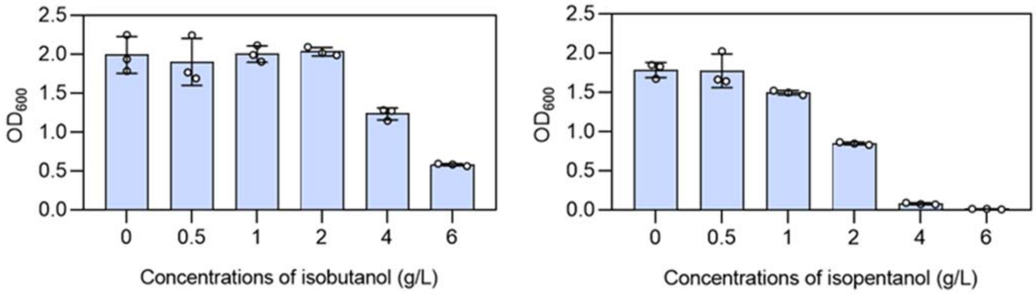

Figure s14. Verification of the isobutanol and isopentanol tolerance of *E. coli* MG1655. Values and error bars represent mean and s.d. (n = 3), respectively.

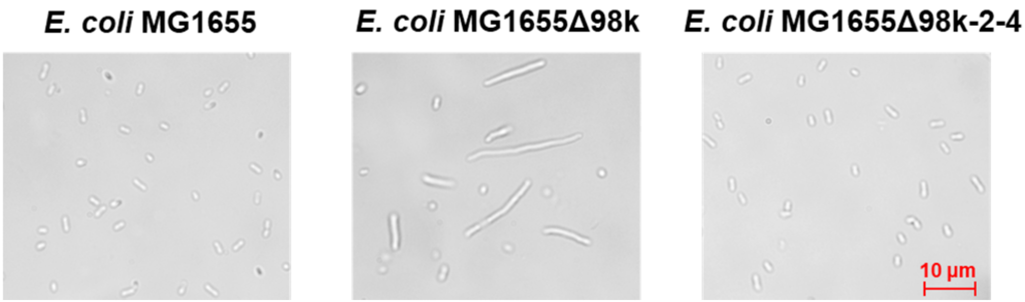

Figure s15. Morphology of *E. coli* MG1655, *E. coli* MG1655Δ98k and *E. coli* MG1655Δ98k-2-4 under microscope.

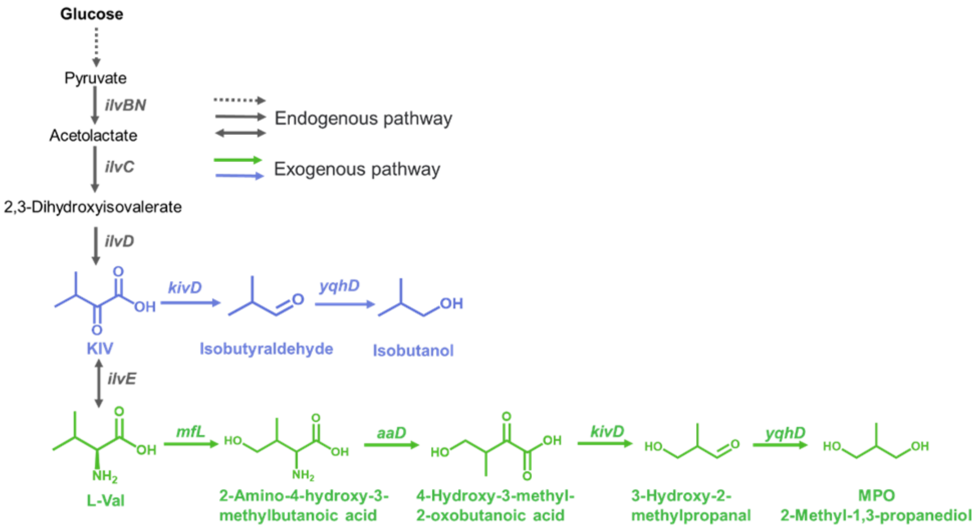

Figure s16. The biosynthetic pathway of MPO (2-methyl-1,3-propanediol). MfL, hydroxylase; AaD, L-amino acid deaminase.

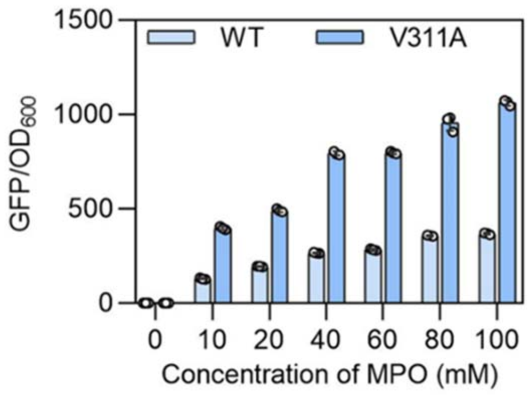

Figure s17. The response effect of wild-type BmoR and BmoR^{V311A} towards MPO. Values and error bars represent mean and s.d. (n = 3), respectively.

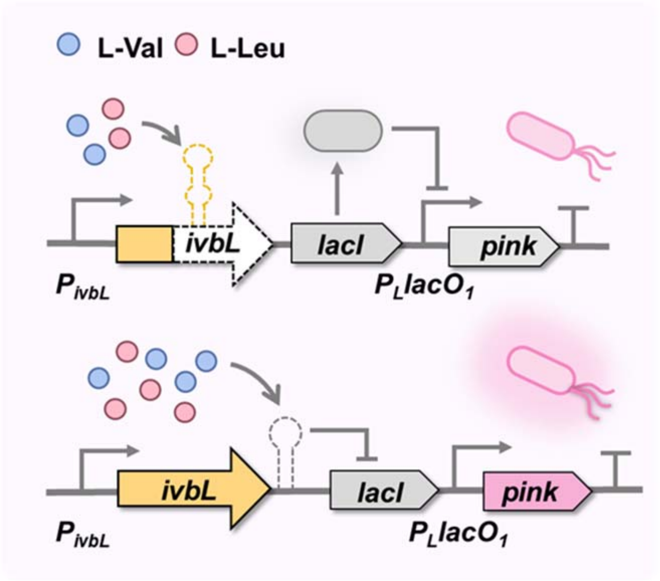

Figure s18. Construction of a visual high-throughput screening system. This system was based on an inducible cascade system with a visual protein pink as output.

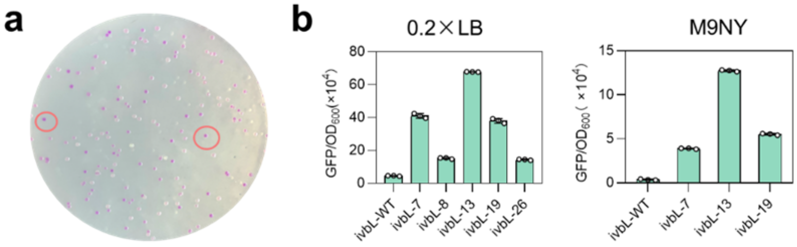

Figure s19. Screening of *ivbL* mutants. **a** The response effect of *ivbL* mutants on the plate. **b** The response effect of *ivbL* mutants-dependent inducible cascade system with GFP as output. Medium 0.2×LB or M9NY was used. Values and error bars represent mean and s.d. (n = 3), respectively.

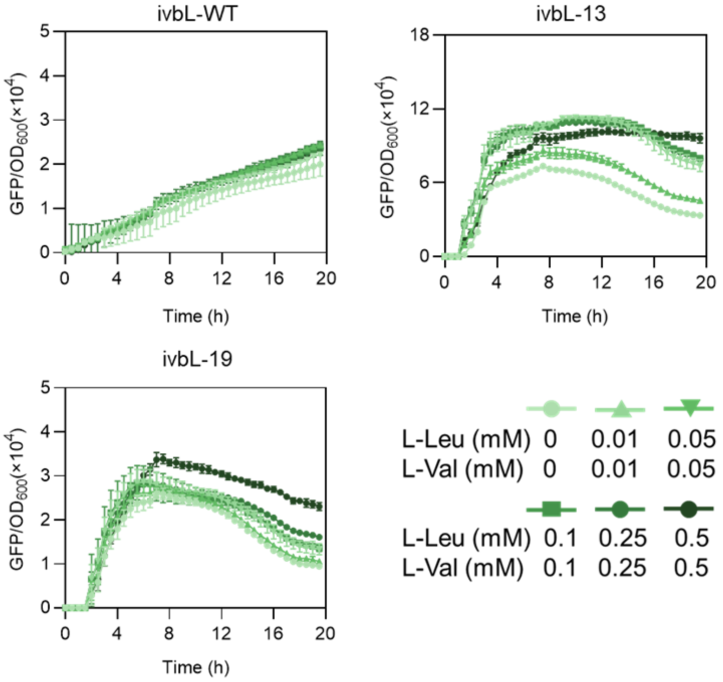

Figure s20. Fluorescence kinetic detection of highly responsive *ivbL* mutants-based inducible cascade regulatory system. Values and error bars represent mean and s.d. (n = 3), respectively.

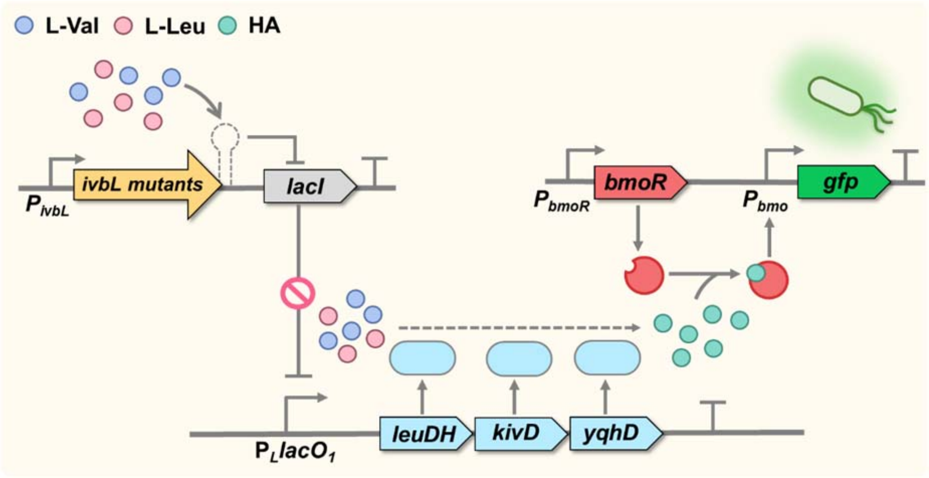

Figure s21. Introduction of highly responsive *ivbL* mutants into the two-layer cascade system to enable the precise inducible regulation of HA production by AA availability.

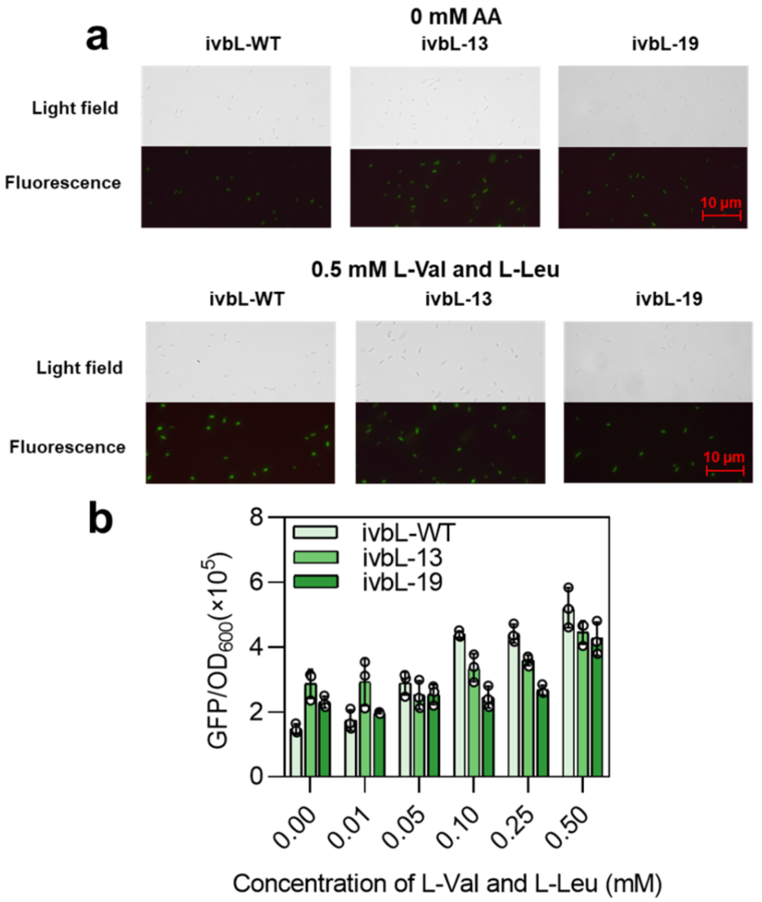

Figure s22. The response effect of the two-layer cascade system in **Figure s15**. **a** The fluorescence intensity under fluorescence microscopy. **b** The response effect of this system with different concentration of L-Val and L-Leu addition.

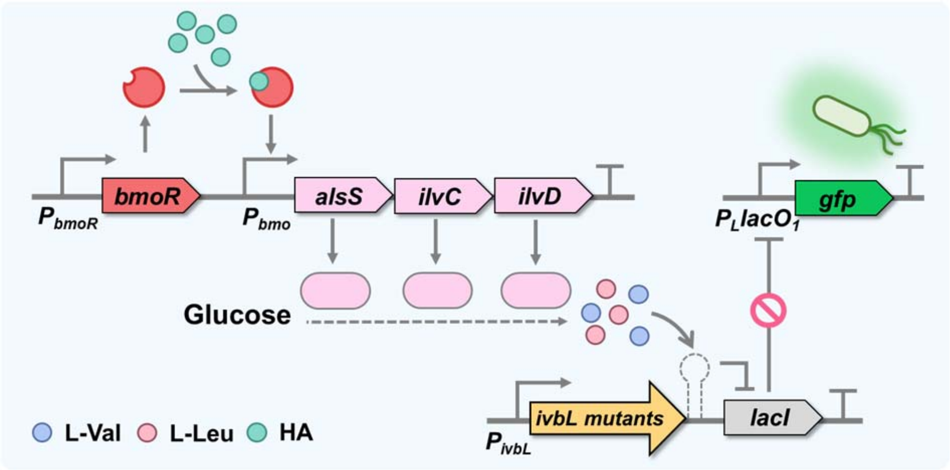

Figure s23. Introduction of highly responsive *ivbL* mutants into the two-layer cascade system to enable the precise inducible regulation of AA biosynthesis by HA availability.

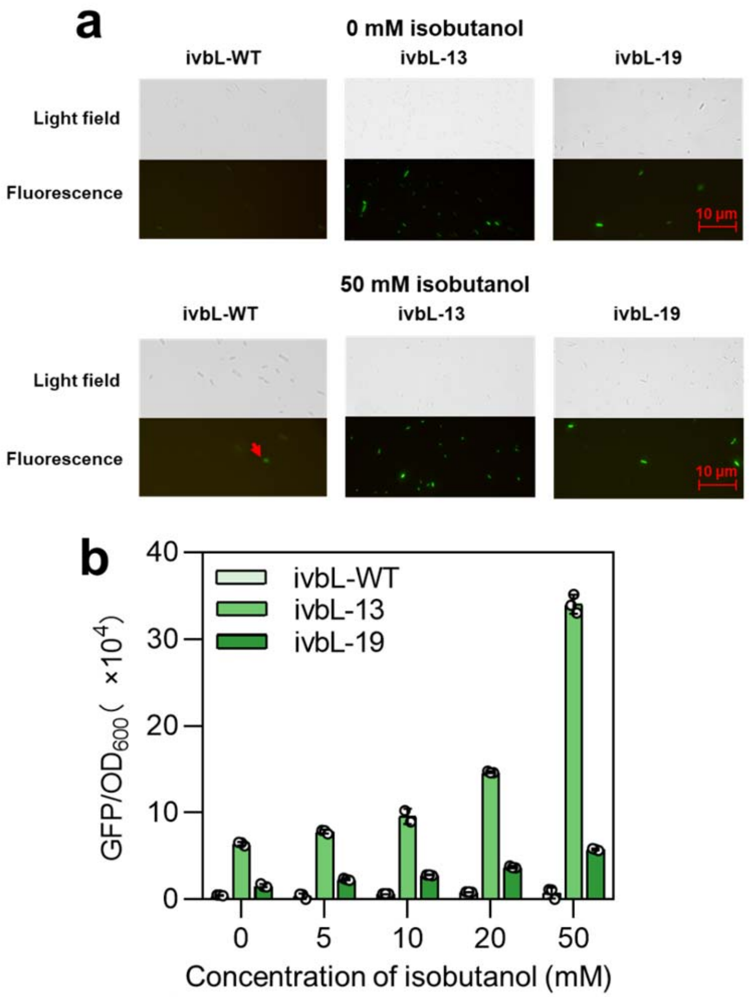

Figure s24. The response effect of the two-layer cascade in **Figure s17**. **a** The fluorescence intensity under fluorescence microscopy. **b** The response effect of this system with different concentration of isobutanol addition. Values and error bars represent mean and s.d. (n = 3), respectively.

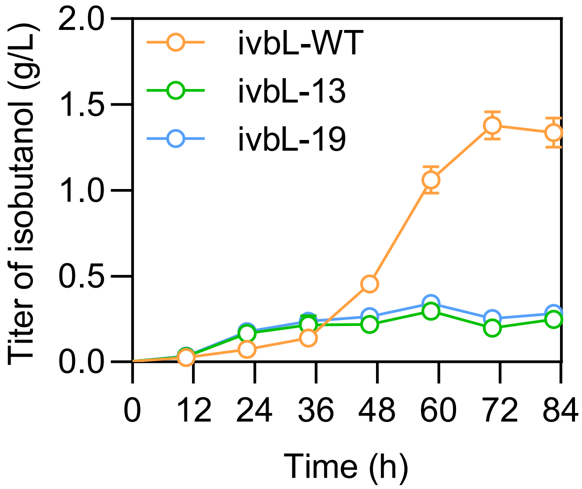

Figure s25. Shake flask fermentation of highly responsive *ivbL* mutants-contained CRUISE. *E. coli* MG1655Δ*lacIZYA* strain harboring pS-B-AII and pS-iL-LKY (ivbL-WT), *E. coli* MG1655Δ*lacIZYA* strain harboring pS-B-AII and pS-i13L-LKY (ivbL-13), and *E. coli* MG1655Δ*lacIZYA* strain harboring pS-B-AII and pS-i19L-LKY (ivbL-19) were used. Medium M9NY was used. Values and error bars represent mean and s.d. (n = 3), respectively.

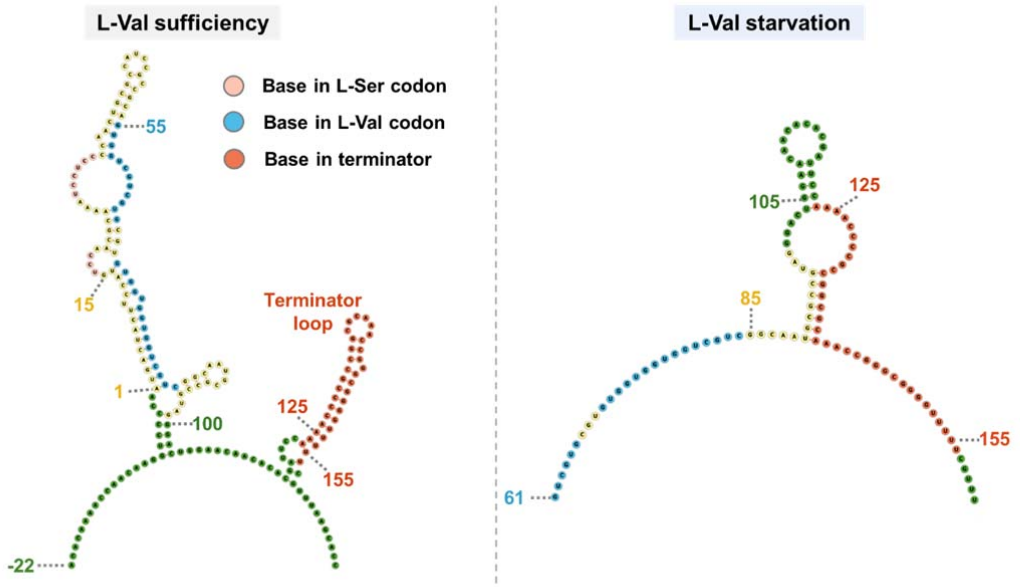

Figure s26. The second structures of L-Val-specific response *ivbL* mutant under the sufficient and deficient L-Val.

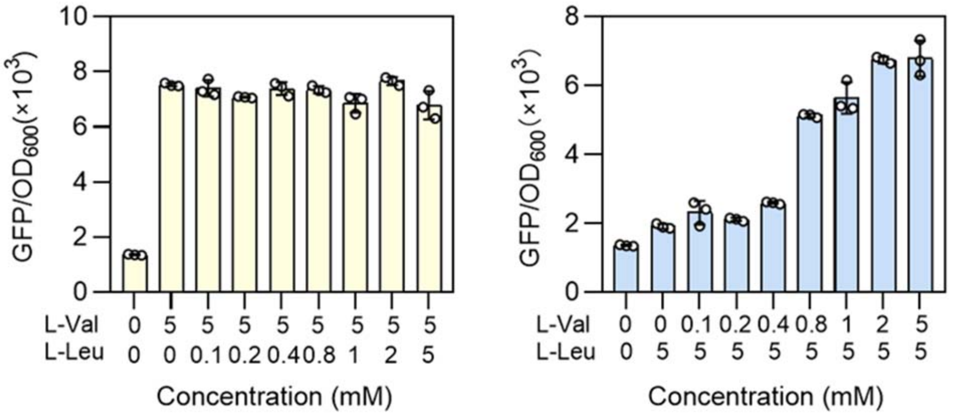

Figure s27. The response effect of the inducible cascade regulatory system containing L-Val-specific *ivbL* mutant. Values and error bars represent mean and s.d. (n = 3), respectively.

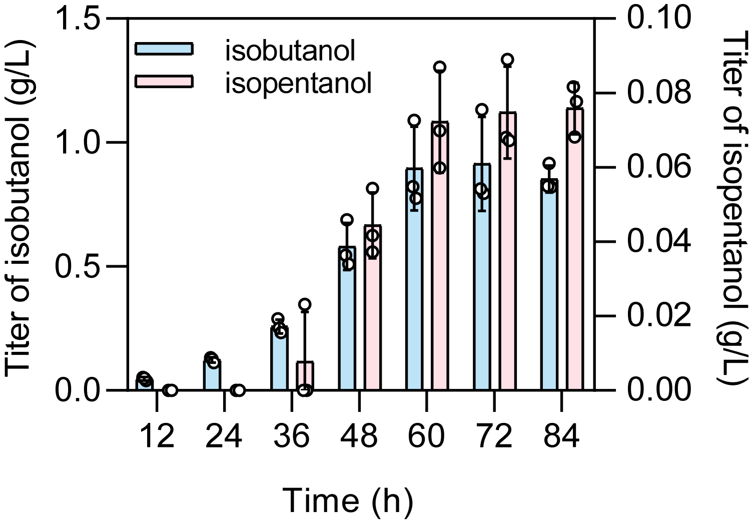

Figure s28. HA titers of the strain with L-Val-specific *ivbL* mutant-contained CRUISE. *E. coli* MG1655Δ*lacIZYA* strain harboring pS-B-AII and pS-iSL-LKY was used. Medium M9NY was used. Values and error bars represent mean and s.d. (n = 3), respectively.

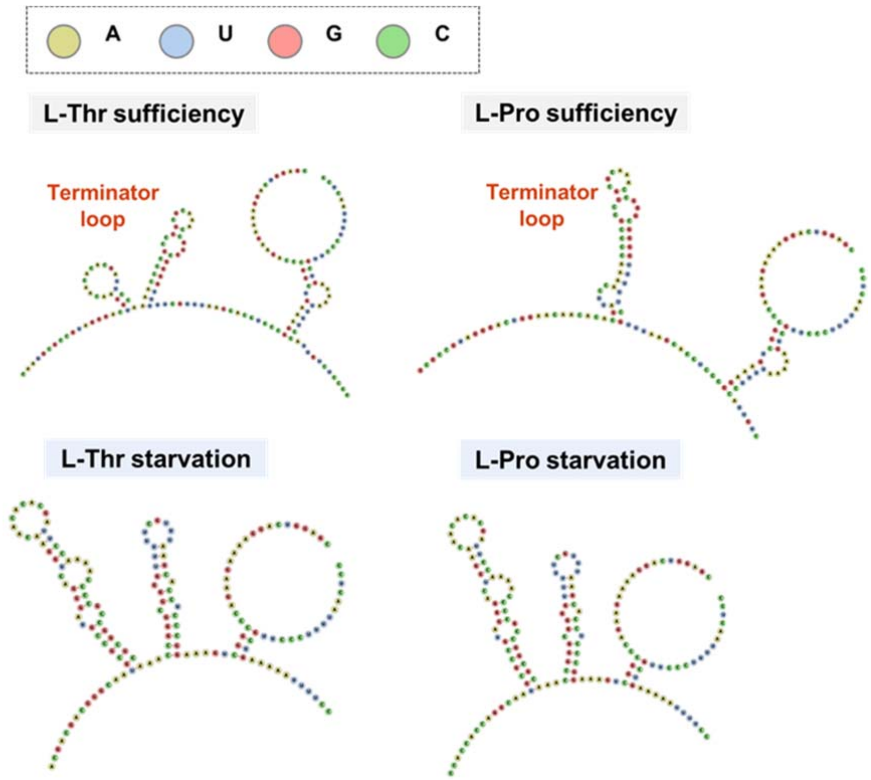

Figure s29. The second structures of *ivbL* mutants whose L-Val and L-Leu codons were replaced with L-Thr or L-Pro codons. These *ivbL* mutants can form terminator loops under sufficient AA.

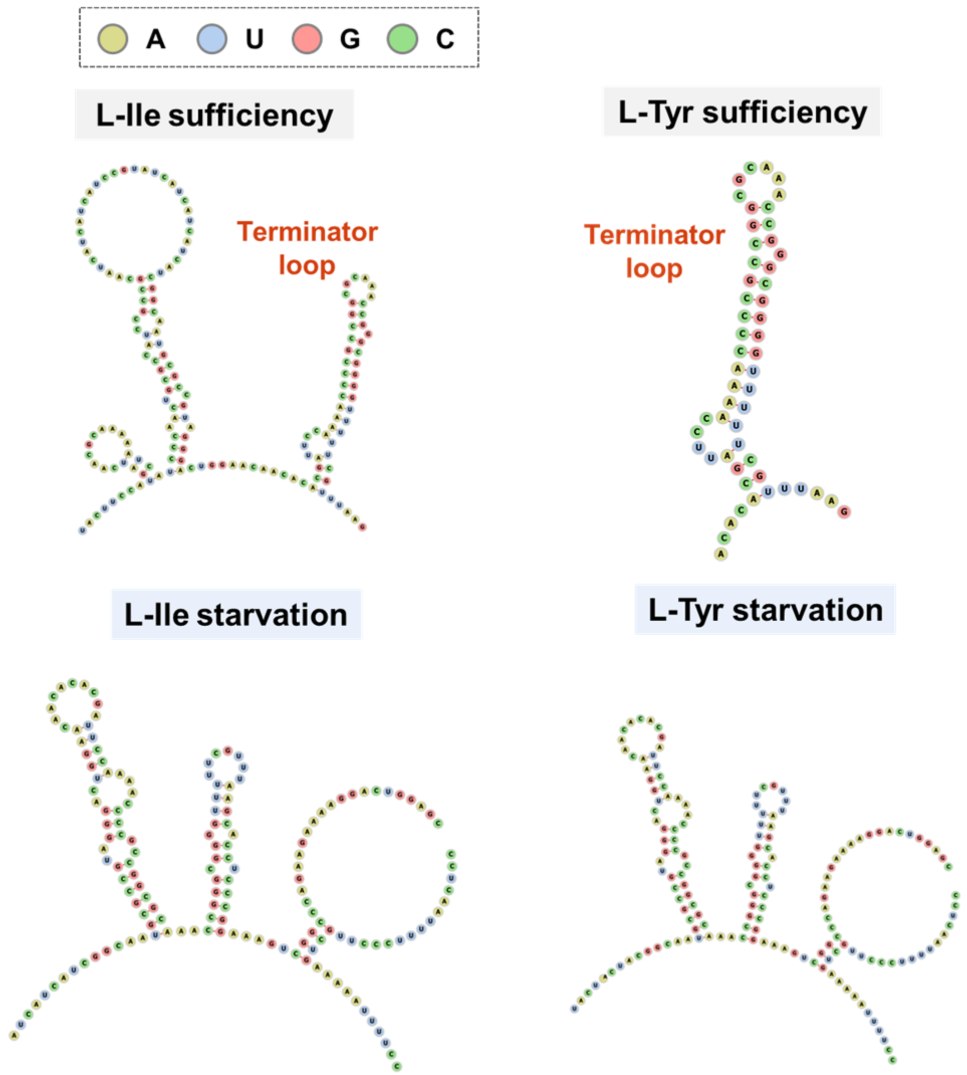

Figure s30. The second structures of *ivbL* mutants whose L-Val and L-Leu codons were replaced with L-Ile or L-Tyr codons. These *ivbL* mutants can form terminator loops under sufficient AA.

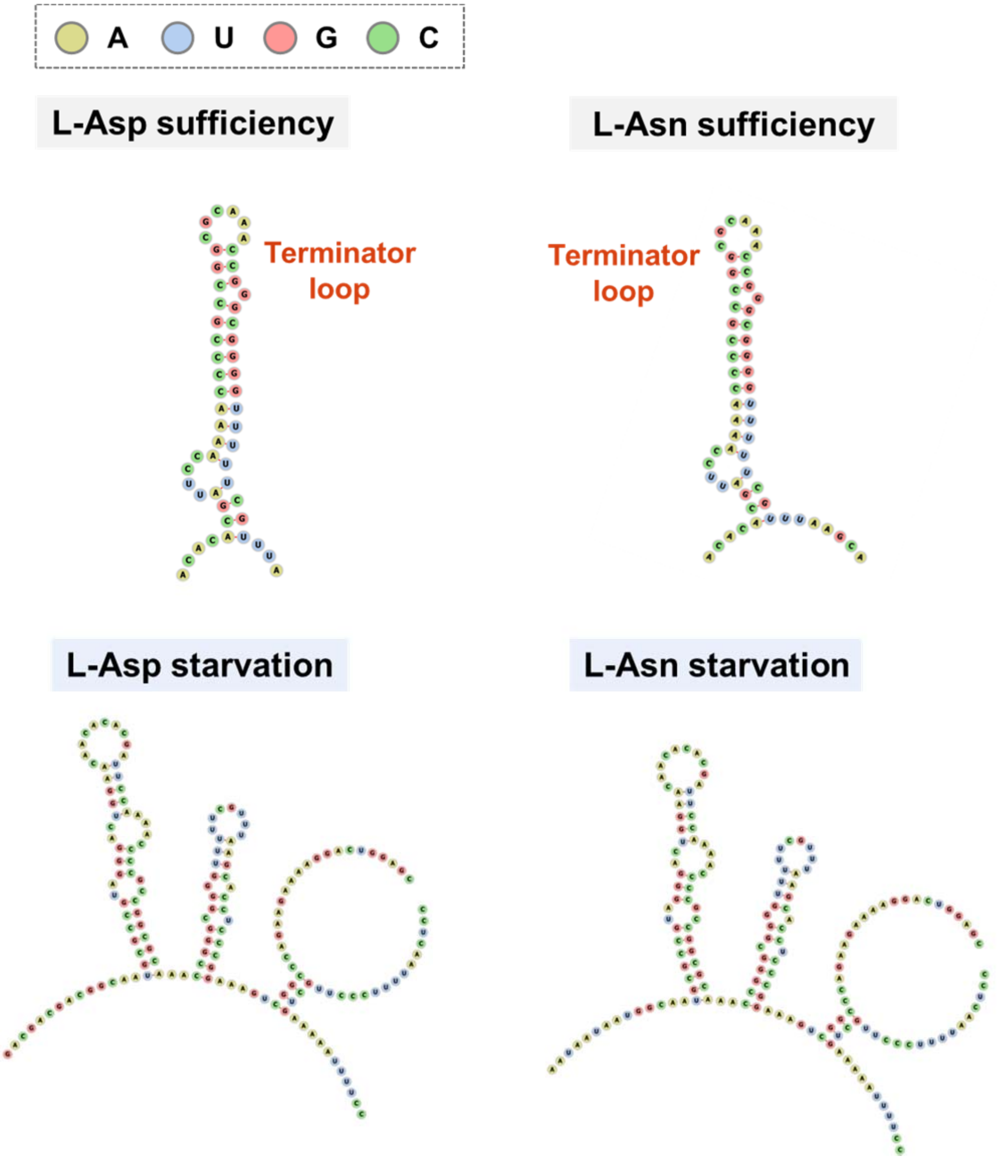

Figure s31. The second structures of *ivbL* mutants whose L-Val and L-Leu codons were replaced with L-Asp or L-Asn codons. These *ivbL* mutants can form terminator loops under sufficient AA.

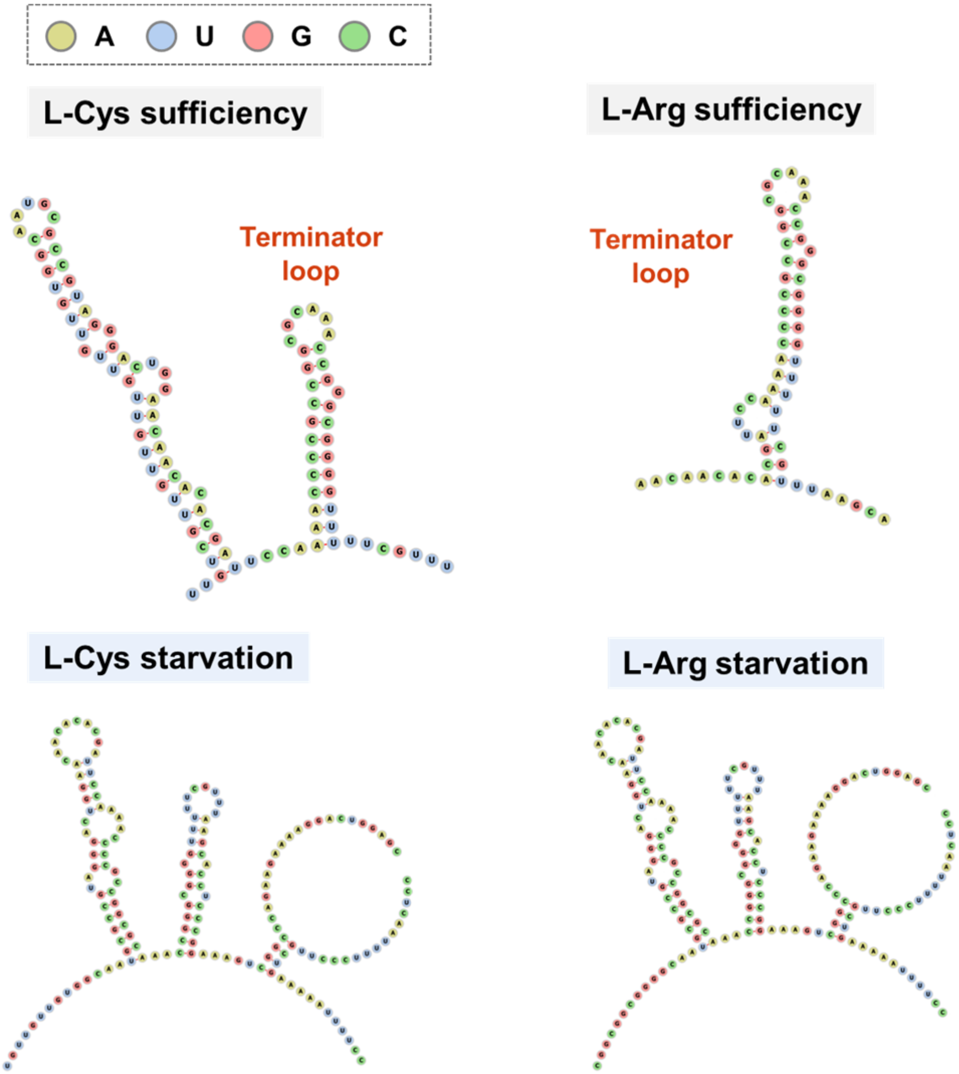

Figure s32. The second structures of *ivbL* mutants whose L-Val and L-Leu codons were replaced with L-Cys or L-Arg codons. These *ivbL* mutants can form terminator loops under sufficient AA.

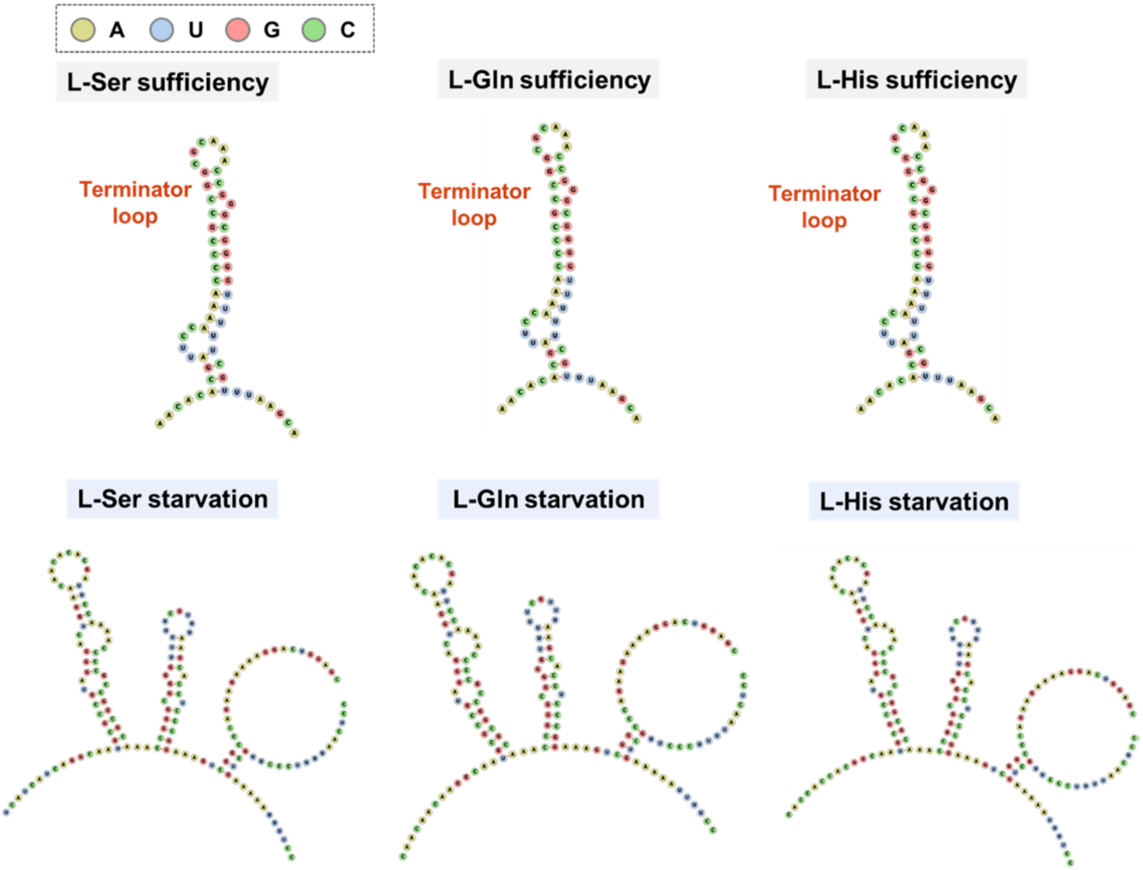

Figure s33. The second structures of *ivbL* mutants whose L-Val and L-Leu codons were replaced with L-Ser, L-Gln or L-His codons. These *ivbL* mutants can form terminator loops under sufficient AA.

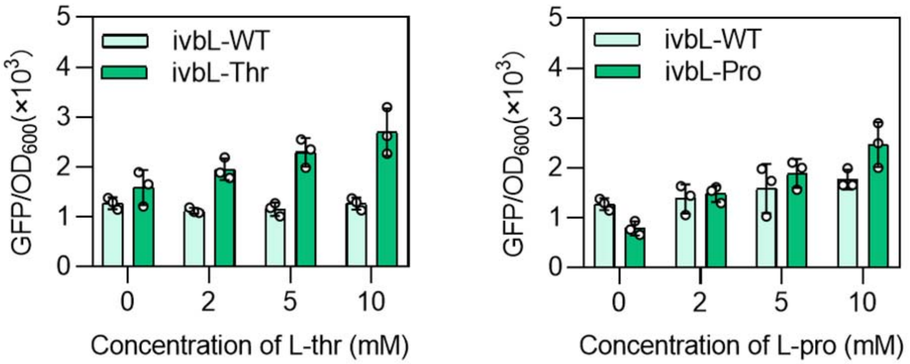

Figure s34. The response effect of inducible cascade regulatory system containing *ivbL* mutants whose L-Val and L-Leu codons were replaced with L-Thr or L-Pro codons. Values and error bars represent mean and s.d. (n = 3), respectively.

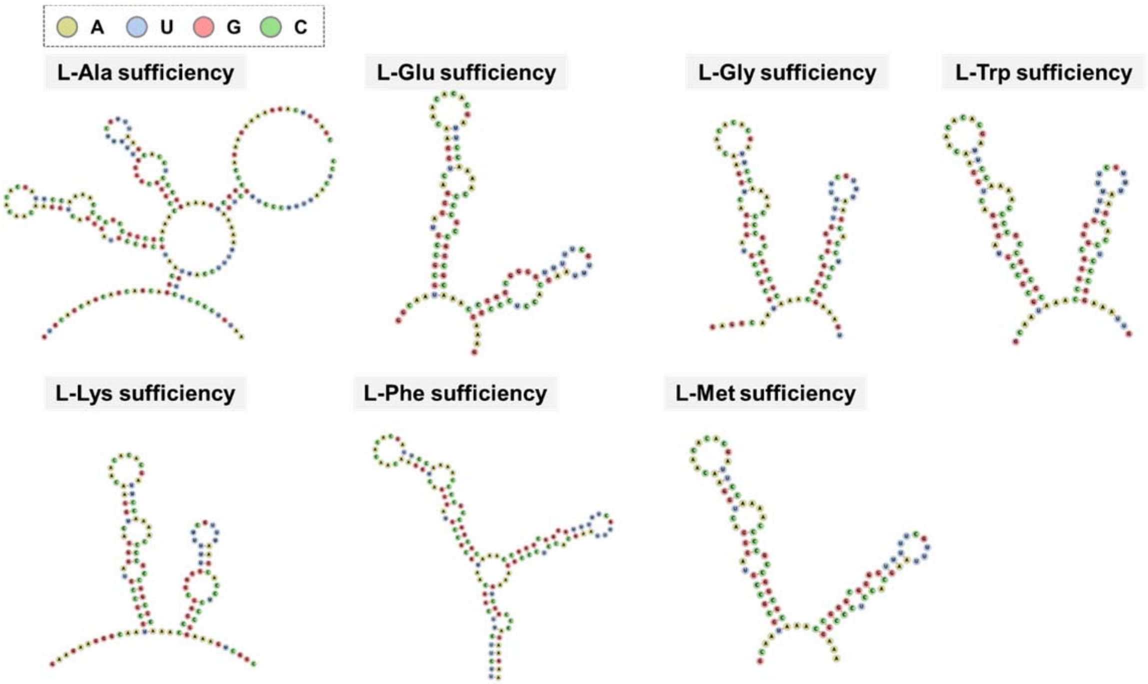

Figure s35. The second structures of *ivbL* mutants whose L-Val and L-Leu codons were replaced with L-Ala, L-Glu, L-Gly, L-Trp, L-Lys, L-Phe or L-Met codons. These *ivbL* mutants cannot form terminator loops under sufficient AA.
